# Microrings as programmable temporal kernels enabling photonic AI beyond 100 Gbaud

**DOI:** 10.1038/s41467-026-77446-8

**Published:** 2026-09-25

**Authors:** Shaojie Liu, Tengji Xu, Benshan Wang, Jiayong Peng, Qiarong Xiao, Li Fan, Hongwei Chen, Chaoran Huang

**Affiliations:** 1 Department of Electronic Engineering, The Chinese University of Hong Kong, Hong Kong SAR, China; 2 Department of Electronic Engineering, Tsinghua University, Beijing, China; 3 Beijing National Research Center for Information Science and Technology (BNRist), Beijing, China

**Keywords:** Integrated optics, Optical physics

## Abstract

The rapid growth of artificial intelligence (AI) demands high-performance hardware accelerators. Photonic computing with microring resonators (MRRs) has attracted significant interest, but conventional architectures use MRRs primarily as scalar weights, with speed constrained by the resonance linewidth of  ~ 10 GHz. Here, we redefine MRRs as programmable temporal convolution kernels by exploiting their impulse responses, enabling computation beyond the resonance linewidth. A single MRR operating at a symbol rate of 128 Gbaud achieves a computing throughput of 5.12 trillion operations per second (TOPS), representing a 160-fold improvement over scalar weighting. By integrating temporal convolution with wavelength- and space-division multiplexing, we realize a multi-channel MRR convolution engine with 120.8 TOPS throughput and reduce the hardware complexity from O(*N*^2^) to O(*N*), achieving a compute density of 48.05 TOPS/mm^2^. The framework is experimentally validated through optical modulation format identification, network anomaly detection, and image classification, establishing a scalable computing primitive for photonic AI accelerators.

## Abstract (web summary)

Artificial intelligence is driving demand for faster and scalable computing hardware. Here, authors transform microring resonators into temporal convolution processors, demonstrating ultrafast and hardware-efficient photonic AI accelerators.

## Introduction

The rapid progress and scaling of artificial intelligence (AI), especially large-scale deep neural networks and large language models, have resulted in an unprecedented growth in computational demand^1^. As conventional electronic hardware approaches fundamental limits in computing power and energy efficiency^2,3^, alternative computing paradigms are being actively explored^4^. Among them, analog photonic computing has emerged as a promising candidate due to its intrinsically high bandwidth, massive parallelism, and low-latency signal processing capability^5–12^.

**Fig. 1:**
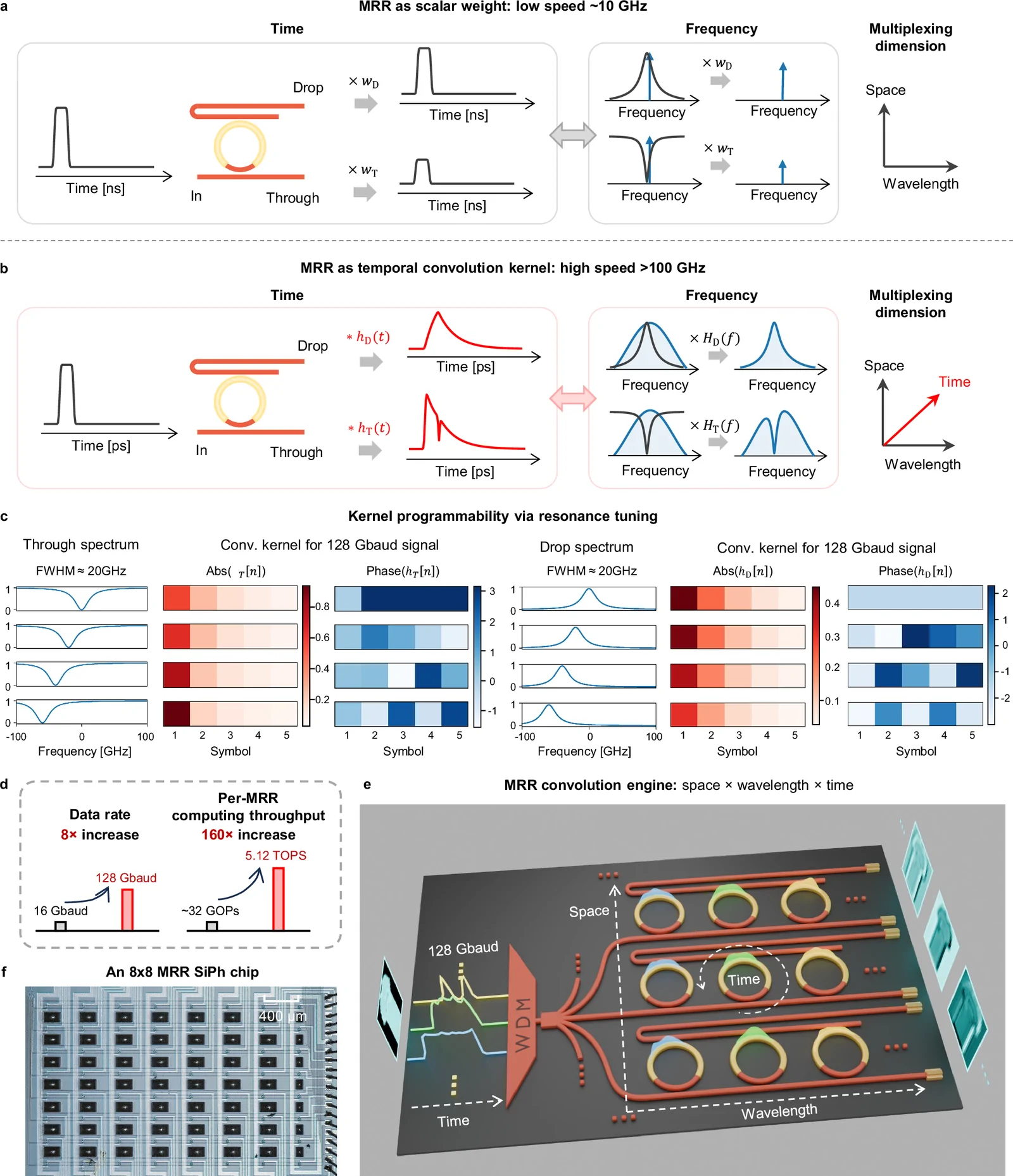
Concept of MRR temporal convolution. **a** Conventional scheme that uses MRRs as scalar weights, where data rate is low and limited by MRR bandwidth to  ~ 10 GHz. **b** The proposed scheme that uses MRRs as temporal convolution kernels, where data rate is high and exceeds MRR bandwidth to over 100 GHz. **c** Representation of the MRR convolution kernel and its programmability. The MRR designed in this work has FWHM of about 20 GHz. For signal at 128 Gbaud, the effective kernel size is 5. The kernel value can be programmed by tuning MRR's resonance frequency. The through and drop ports of the same MRR provide different convolution kernels. **d** The proposed concept offers 8 × increase in data rate and 160 × increase in per-MRR computing throughput. **e** Concepts of the multi-channel and high-dimensional MRR convolution engine that combines temporal convolution, SDM, and WDM. **f** An 8 × 8 MRR convolution engine on a silicon photonic chip.

Microring resonators (MRRs) are widely employed in integrated photonic computing because of their compact footprint, energy-efficient tuning, and natural compatibility with wavelength-division multiplexing (WDM)^5,8,13^. In conventional computing architectures, an MRR is treated as a scalar weighting element, where tuning its resonance simply modulates the transmission of a carrier frequency to implement a single multiplication (Fig. 1a)^14^. This model requires that the signal bandwidth is much smaller than the resonance linewidth. The intrinsic temporal and spectral dynamics of the MRR are largely ignored, and the per-device computing throughput is fundamentally constrained to several giga-operations per second (GOPS)^15–21^.

In this work, we propose a different paradigm: we redefine a single microring resonator as a programmable, complex-valued temporal convolution kernel rather than a scalar weight. When the signal bandwidth exceeds the resonance linewidth, an MRR naturally operates as a linear time-invariant (LTI) filter whose impulse response performs convolution in the time domain (Fig. 1b). By exploiting this property, one MRR is elevated from executing a single multiplication per symbol to implementing a one-dimensional (1D) convolution across multiple symbols, whose convolutional kernel values are continuously programmable through resonance tuning (Fig. 1c). Furthermore, the convolution kernel size is dynamically programmable by adjusting the symbol rate, providing an additional degree of freedom for network optimization.

This paradigm introduces two major advantages. First, it transforms the conventional interpretation of the MRR bandwidth limit. Instead of restricting the signal data rate, the resonance linewidth determines the temporal extent of the impulse response. Consequently, the signal bandwidth can substantially exceed the MRR full-width-at-half-maximum (FWHM) bandwidth without degrading computational correctness. Second, it dramatically increases the per-device computing throughput. In conventional MRR-based architectures, each MRR contributes only one scalar multiplication per symbol. In contrast, when operated as a temporal convolution kernel, a single MRR simultaneously performs multiple complex-valued multiply–accumulate operations over the kernel length for every input symbol. At ultra-high symbol rates, this allows one resonator to reach computing throughput of multiple trillion operations per second (TOPS), representing orders-of-magnitude improvement in computational throughput compared with the traditional weighting scheme. In this work, we experimentally demonstrate a single MRR performing temporal convolution at the symbol rate of 128 Gbaud, providing a computing throughput of 5.12 TOPS. It offers an 8 × higher data rate and a 160 × higher computing throughput compared with a conventional MRR operating as a scalar weight at 16 Gbaud (Fig. 1d), whose performance is limited by its FWHM bandwidth.

Building on this concept, we develop a multi-channel high-dimensional MRR-based photonic convolution engine that combines temporal convolution with space-division-multiplexing (SDM) and WDM, as shown in Fig. 1e. Each MRR performs an independent temporal convolution on a wavelength channel, while photodetection naturally sums the outputs from multiple wavelengths, enabling high-dimensional convolution in a compact and scalable architecture. This architecture reduces the hardware complexity of an *N* × *N* convolution kernel from O(*N*^2^) to O(*N*), leading to a record compute density of 48.05 TOPS/mm^2^.

While some prior works utilize the filtering properties of MRRs for specific computing operations, such as differentiation^22^, integration^23^, and spectrum slicing^24^, this work reinterprets MRRs as programmable and trainable convolution kernels compatible with standard backpropagation-based training, thereby enabling their direct integration into deep photonic neural networks (PNNs). We validate the universality and effectiveness of this convolution paradigm through three representative neural network tasks. In the modulation format identification (MFI) task, we demonstrate that a single MRR convolution kernel enhances the MFI accuracy of optical communication signals from 71.0% to 99.8%. In the network anomaly detection (NAD) task, we show that operating at higher speed can even provide higher accuracy in the proposed computing architecture. The classification accuracy of 20-class network traffic data increases from 93.3% at 1 Gbaud to 96.4% at 128 Gbaud. In the Fashion-MNIST^25^ image classification task, we propose a new paradigm of 2D image convolution with a 4.25-fold reduction in hardware usage, while simultaneously delivering a 27-fold increase in computing throughput to 120.832 TOPS.

By redefining the computational role of an MRR from a scalar weighting element to a programmable temporal convolution kernel, this work establishes a new paradigm for MRR-based photonic computing. It overcomes the conventional bandwidth limitation associated with resonance linewidth, dramatically enhances per-device computing throughput, and enables compact, scalable, and high-dimensional convolution engines for PNNs. This framework opens a new route toward high-performance, energy-efficient, and highly integrated photonic AI accelerators.

## Results

### Ultrafast convolution based on impulse response of MRR

In conventional photonic computing schemes, MRRs are predominantly used as scalar weighting elements, as illustrated in Fig. 1a. By tuning the resonance frequency of an MRR, the transmission at the optical carrier is adjusted, resulting in different weighting coefficients applied to the input signal^14^. This description, however, is only an approximation of the actual MRR response and is valid strictly when the bandwidth of the input signal is much smaller than the FWHM of the resonance. Once the signal bandwidth approaches or exceeds the MRR linewidth, the weighting model breaks down and inter-symbol interference inevitably appears. In this regime, the MRR should no longer be interpreted as a scalar multiplier, but rather as a frequency-domain filter, as shown in Fig. 1b. Correspondingly, in the time domain, this operation is equivalent to a convolution between the input signal and the impulse response of the resonator.

In this work, we explicitly exploit this convolutional nature of the MRR and elevate it from a weighting element to a programmable temporal convolution kernel for PNNs. Instead of avoiding the bandwidth regime where filtering dominates, we intentionally operate the MRR in this regime and utilize its impulse response as a computational resource. This forms the physical basis of our ultrafast MRR convolution engine.

According to coupled-mode theory, the frequency responses of the through and drop ports of an add-drop MRR are given by Eqs. (1)–(2)^26^:

1 $${H}_{{{{\rm{T}}}}}(f)=\frac{{t}_{1}-a{t}_{2}{{{{\rm{e}}}}}^{-j2\pi (f+{f}_{{{{\rm{tune}}}}})\tau }}{1-a{t}_{1}{t}_{2}{{{{\rm{e}}}}}^{-j2\pi (f+{f}_{{{{\rm{tune}}}}})\tau }}$$

2 $${H}_{{{{\rm{D}}}}}(f)=\frac{-j{\kappa }_{1}{\kappa }_{2}\sqrt{a}\,{{{{\rm{e}}}}}^{-j\pi (f+{f}_{{{{\rm{tune}}}}})\tau }}{1-a{t}_{1}{t}_{2}{{{{\rm{e}}}}}^{-j2\pi (f+{f}_{{{{\rm{tune}}}}})\tau }}$$

where *κ*_1_ and *κ*_2_ are the coupling coefficients between the MRR and the through and drop waveguides, respectively, with ${t}_{1}=\sqrt{1-{\kappa }_{1}^{2}}$ and ${t}_{2}=\sqrt{1-{\kappa }_{2}^{2}}$. The parameter *a* denotes the round-trip attenuation factor, and *τ* denotes the round-trip time of the ring. *f*_tune_ denotes the frequency detuning between the MRR resonance and the optical carrier, and we define *f*_tune_ = 0 when the resonance is aligned with the carrier frequency.

Taking the inverse Fourier transform of Eqs. (1)–(2) yields the continuous-time impulse responses:

3 $${h}_{{{{\rm{T}}}}}(t)={t}_{1}\delta (t)+{\sum }_{m=1}^{+\infty }{\left(a{t}_{1}{t}_{2}{{{{\rm{e}}}}}^{-j2\pi {f}_{{{{\rm{tune}}}}}t}\right)}^{m}\left({t}_{1}-\frac{1}{{t}_{1}}\right)\delta (t-m\tau )$$

4 $${h}_{{{{\rm{D}}}}}(t)=-j{\kappa }_{1}{\kappa }_{2}\sqrt{a}\,{{{{\rm{e}}}}}^{-j\pi {f}_{{{{\rm{tune}}}}}\tau }{\sum }_{m=0}^{+\infty }{\left(a{t}_{1}{t}_{2}{{{{\rm{e}}}}}^{-j2\pi {f}_{{{{\rm{tune}}}}}t}\right)}^{m}\delta \left(t-\left(m+\frac{1}{2}\right)\tau \right)$$

For a continuous-time input signal *x*(*t*), the MRR output can be expressed as the convolution *y*(*t*) = *x*(*t*)*h(*t*), indicating that the resonator inherently acts as a convolution operator.

Convolution in digital convolutional neural networks (CNNs) is typically implemented using discrete kernels with finite size. To enable a direct comparison with digital CNNs, the impulse response of an MRR can be represented as an equivalent discrete convolution kernel, $h[n]=\int_{(n-1)/B}^{n/B}h(t)\,{{{\rm{d}}}}t$, where *B* denotes the symbol rate, *n* = 1, 2, …, *N*. As the impulse response of a passive MRR (*at*_1_*t*_2_ < 1) decays exponentially in time, we define the effective kernel size *N* as the largest integer index satisfying ∣*h*[*n*]∣ > 0.1∣*h*[1]∣, because contributions from coefficients beyond this range have minimal impact to the convolution result (see Supplementary Note 2). Consequently, the optical convolution by MRR can be approximately described as: $y[n]={\sum }_{i=1}^{N}x[n-i+1]h[i]$. Increasing the symbol rate will increase *N*, which generally expands the receptive field of the convolutional kernel, allowing the neural network to extract broader contextual features.

After optical convolution, the photodetector performs optical-to-electrical conversion and inherently generates square-law and cross-term nonlinearities through photodetection. These nonlinearities naturally serve as the activation function in PNNs. The detected signal can be expressed as Eq. (5):

5 $${y}_{{{{\rm{PD}}}}}[n]=| y[n]{| }^{2}={\left| {\sum }_{i=1}^{N}x[n-i+1]h[i]\right| }^{2}$$

By combining temporal convolution with WDM, the convolution operation can be extended to higher dimensions. For two-dimensional (2D) image convolution, one dimension is encoded in time while the other is encoded in wavelength, as shown in Fig. 1e. MRRs with different resonance wavelengths coupled to the same bus waveguide independently perform temporal convolution on each wavelength channel. The photodetector then sums the optical power from all wavelengths, yielding

6 $${y}_{{{{\rm{PD}}}},{{{\rm{sum}}}}}[n]=\mathop{\sum }_{k=1}^{K}| {y}_{k}[n]{| }^{2}={\sum }_{k=1}^{K}{\left| {\sum }_{i=1}^{N}x[n-i+1]{h}_{k}[i]\right| }^{2}$$

where *K* is the number of wavelength channels. Figure 1e illustrates the architecture of the proposed MRR convolution engine, which integrates temporal convolution, wavelength multiplexing, and spatial parallelism to achieve high-speed, high-dimensional and multi-channel convolution for PNNs. In this way, the number of required MRRs and wavelength channels for an *N* × *N* 2D convolution kernel is reduced from *N*^2^ to *N*. Although the degrees of freedom (DOF) of the kernel are simultaneously reduced to *N*, trained MRR kernels can achieve performance comparable to fully unconstrained kernels in PNNs.

For experimental demonstrations, we design and fabricate an 8 × 8 MRR convolution engine on a silicon-on-insulator platform, as shown in Fig. 1f. A three-stage cascade of Y-splitters broadcasts the input signal into eight parallel rows. In each row, eight add-drop MRRs with different resonance wavelengths are coupled to a common bus waveguide, enabling simultaneous convolution on eight wavelength channels. Thermal isolation trenches are introduced to suppress thermal crosstalk among adjacent MRRs. The chip is packaged using wire bonding for thermal tuning and a fiber array for optical input and output. Details of the chip characterization are provided in Supplementary Note 1.

### Programming and training of the MRR convolution kernel

The convolution kernel implemented by the MRR is programmable through tuning its resonance frequency, which is physically controlled by the current applied to the thermo-optic phase shifter integrated on the MRR. As shown in Eqs. (3)–(4), the resonance detuning *f*_tune_ appears in the complex exponential terms of the impulse response *h*(*t*). Varying  *f*_tune_ therefore modifies the phase evolution of *h*(*t*). When mapped to the discrete kernel, it results in controllable variations in both the amplitude and phase of the kernel coefficients *h*[*n*]. This provides a direct physical mechanism for programming the convolution kernel implemented by the MRR, as illustrated in Fig. 1c.

Accurate programming of large-scale MRR arrays is challenged by fabrication non-uniformity, thermal crosstalk, and environmental fluctuations. The characterizations of fabrication variations and thermal crosstalk coefficient are provided in Supplementary Notes 12 and 13, respectively. To address these challenges, we employ the chip-in-the-loop^27^ approach for on-chip configuration. This method enables efficient and accurate programming of large-scale MRR arrays to the desired detuning positions while inherently accounting for fabrication imperfections and thermal crosstalk effects. (see Supplementary Note 14 for details).

The MRR convolution engine can be trained by backpropagation^28^, a standard neural network training algorithm, where the resonance frequency of each MRR serves as the trainable parameter. This distinguishes our approach from reservoir computing approaches^29–31^, in which only the readout layer is trainable. During training, we model the MRR in the frequency domain according to Eqs. (1)–(2). The input signal is first transformed into the frequency domain through Fourier transform, filtered by the frequency response of the MRR, and then transformed back into the time domain through inverse Fourier transform. This frequency-domain model naturally accounts for the full response of the MRR without imposing an artificial truncation on the kernel length, thereby providing a more accurate approximation of the real-world physical system.

In addition, we employ the bandwidth-aware training^32^ approach, which incorporates a low-pass filter into the training model to emulate the bandwidth limitation of the physical system. This approach enables PNNs to perform tasks in the presence of bandwidth-induced signal distortions without requiring pre-emphasis, equalization, or other digital signal processing (DSP) techniques. As a result, the proposed system can reliably perform 128 Gbaud convolution using an experimental channel with a bandwidth of only 38 GHz (see Supplementary Note 3). Furthermore, noise injection^33–35^ and sharpness-aware training^36^ are employed to enhance robustness against experimental noise, fabrication imperfections, and residual tuning errors (see Supplementary Note 4 for details).

### Modulation format identification using a single MRR temporal convolution

To demonstrate the fundamental effectiveness of the MRR convolution paradigm, we first consider the most minimal configuration: a single microring resonator acting as a temporal convolution kernel. Here we use it for optical modulation format identification (MFI), which is a key technology for adaptive and flexible optical networks, where modulation formats and symbol rates are dynamically adjusted according to channel conditions^37^. While deep-learning-based MFI has been extensively studied^38,39^, processing ultrahigh-speed optical signals with low latency remains challenging for conventional electronic hardware. Photonic computing provides a natural solution by directly operating in the optical domain.

**Fig. 2:**
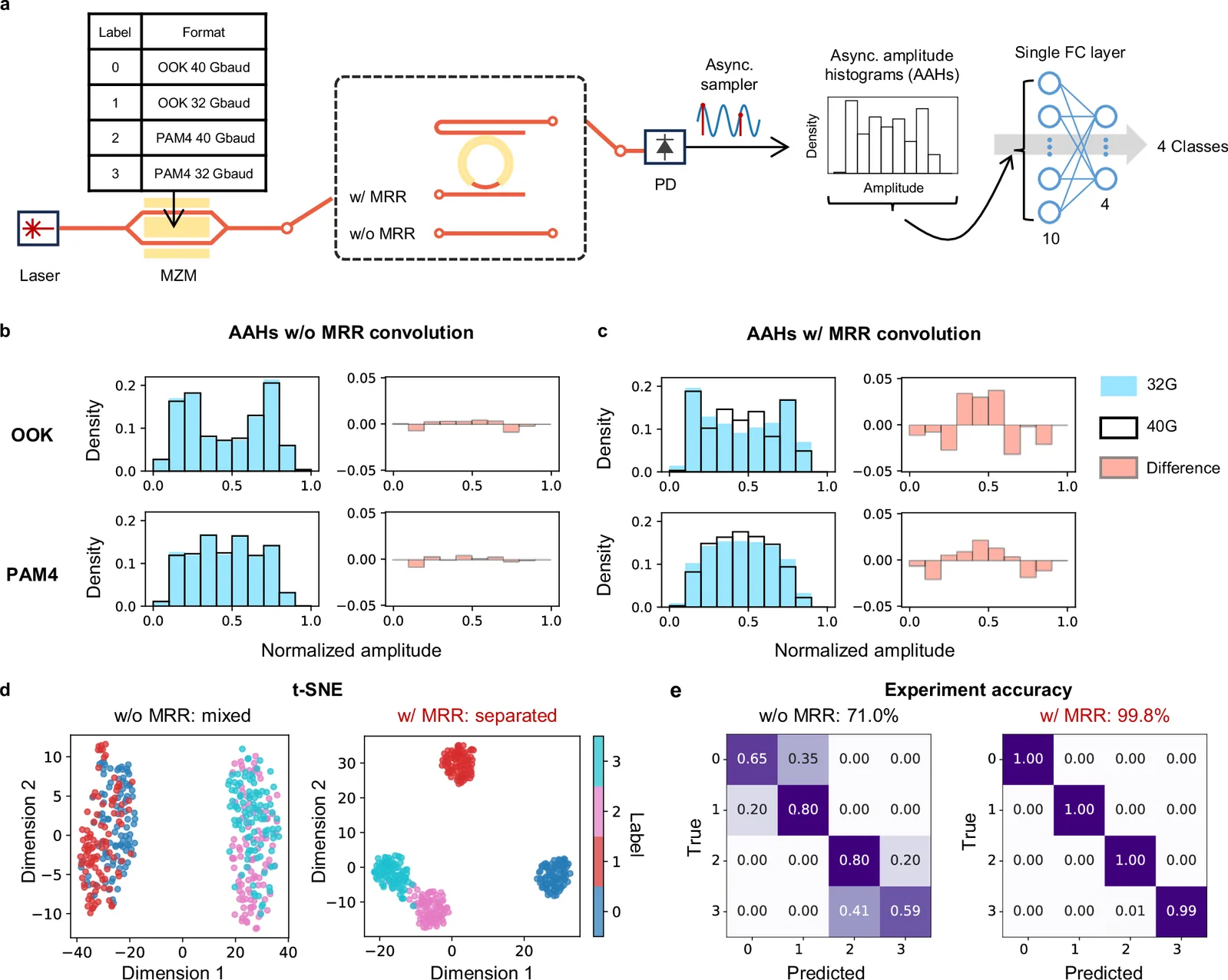
Single MRR temporal convolution for modulation format identification. **a** Concept of modulation format identification with and without MRR. **b** AAHs of different data rates overlap without the MRR. **c** AAHs of different data rates show difference with the MRR convolution. **d** t-SNE with and without MRR. Without the MRR, samples corresponding to different data rates are strongly mixed. With MRR convolution, samples corresponding to different data rates are clearly separated. **e** Classification accuracy of MFI without and with MRR. With MRR convolution, the accuracy increases from 71.0% to 99.8%.

The experimental concept is illustrated in Fig. 2a. Four classes of optical signals are used: OOK (on-off keying) and PAM4 (4-level pulse amplitude modulation) signals at symbol rates of 32 Gbaud and 40 Gbaud. The optical signal is directed to the input port of an MRR in add-drop configuration. The drop port output is detected by a photodetector, after which asynchronous amplitude histograms (AAHs) are constructed from asynchronously sampled waveforms. A single fully connected (FC) layer is then trained to perform modulation format classification (see Methods).

AAHs intrinsically discard temporal ordering and therefore cannot capture time-domain features of the signal. As a result, without any temporal preprocessing, signals with different symbol rates exhibit almost identical AAH distributions and become inseparable by a linear classifier. This limitation is clearly observed in Fig. 2b, where AAHs obtained without the MRR convolution nearly overlap for different data rates, leading to poor classification performance.

By inserting only one MRR before the photodetector, the situation changes fundamentally. Acting as a temporal convolution kernel, the MRR maps time-domain waveform differences into amplitude statistics that become distinguishable in the AAH representation. As shown in Fig. 2c, AAHs after MRR convolution exhibit clearly separated distributions for different modulation formats and symbol rates.

To further visualize the separability of the extracted features, we apply t-distributed stochastic neighbor embedding (t-SNE) to project the ten-dimensional AAH data into a two-dimensional space (Fig. 2d). Without the MRR, samples corresponding to different data rates are strongly mixed. After MRR convolution, not only different modulation formats but also the same format at different symbol rates become well separated, indicating that the single MRR effectively encodes rich temporal information into the statistical domain.

As a result, the classification accuracy is significantly improved, increasing from 71.0% without the MRR to 99.8% with a single MRR (Fig. 2e). This result highlights a central message of our work: even one microring resonator, when reinterpreted as a temporal convolution kernel rather than a scalar weight, can serve as a powerful and compact computational primitive for PNNs.

### Higher speed leads to higher accuracy: ultrafast MRR convolution for network anomaly detection

In conventional photonic computing architectures, higher data rates often result in lower accuracy due to electro-optic modulation distortion and parasitic noise^21^. However, the proposed MRR convolution exhibits a unique advantage: a higher data rate can potentially improve accuracy. This is because higher data rates lead to larger effective kernel size, thereby enlarging the receptive field of the convolution operation and enabling the network to extract broader contextual features. This effect is widely observed in CNNs^40^, although the required receptive fields for optimal feature extraction can vary from task to task. We demonstrate this advantage in a network anomaly detection (NAD) task, which is a critical application for network management and cybersecurity^41^.

**Fig. 3:**
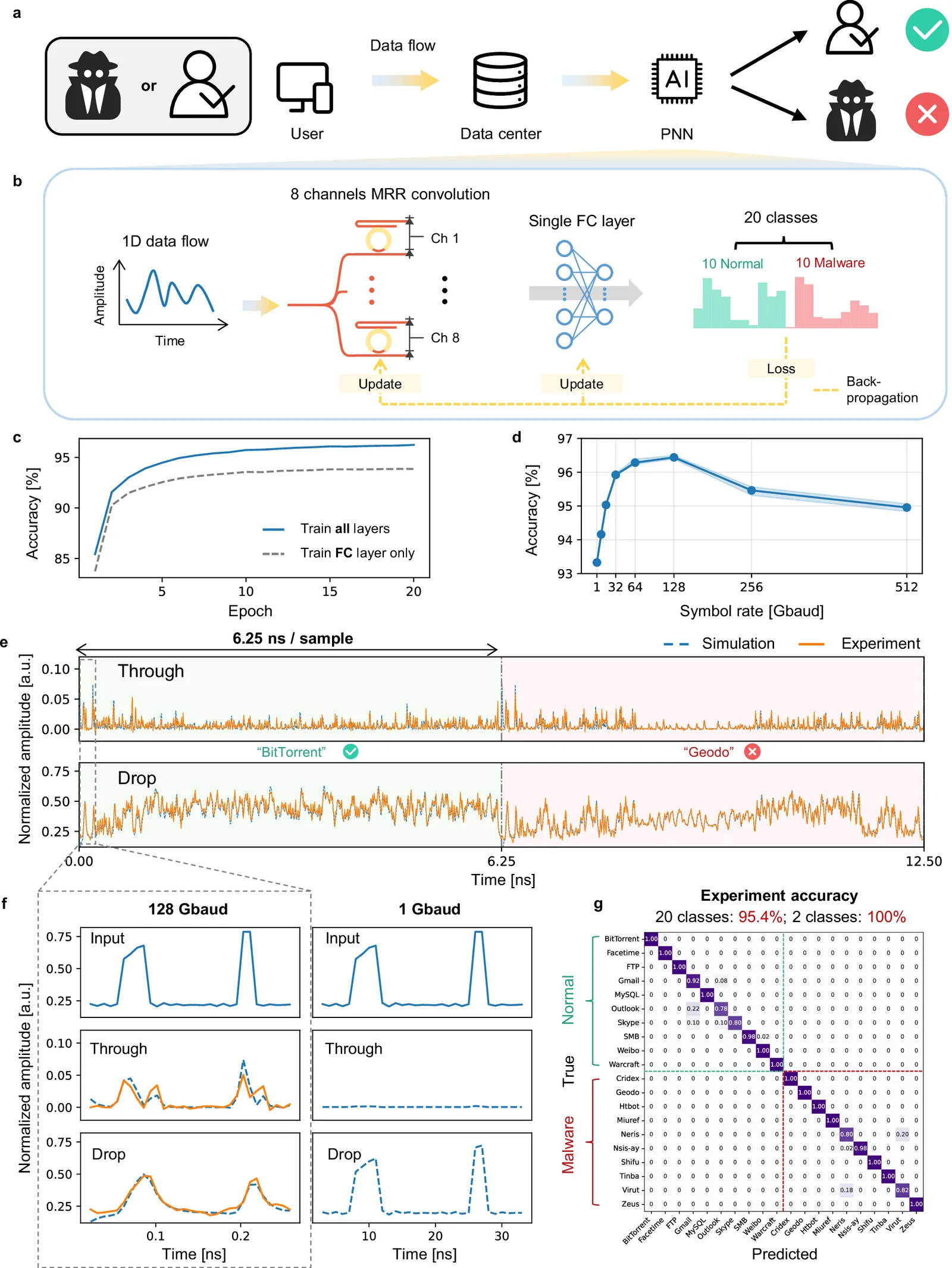
MRR convolution for network anomaly detection. **a** Concept of network anomaly detection. The objective is to classify whether network traffic originates from normal software or malware, enabling real-time intrusion detection in data centers. **b** Neural network design for USTC-TFC2016 dataset. It consists of an 8-channel MRR 1D convolution layer and an FC layer, both of which are trainable using backpropagation. **c** Joint training of all layers achieves higher accuracy than training the FC layer only. **d** Comparison of testing accuracy when training the neural network at different symbol rates in simulations. The accuracy increases from 93.3% at 1 Gbaud to 96.4% at 128 Gbaud, then decreases to 94.9% at 512 Gbaud. The shaded region indicates the maximum-minimum range of accuracies over five independent tests with noise injection. **e** Experimental results of 2 samples at 128 Gbaud, where each sample takes only 6.25 ns. **f** Zoomed-in waveforms of the signal after MRR, showing that through and drop ports extract distinct temporal features at high speed (128 Gbaud), while at low speed (1 Gbaud) they degenerate to simple weighting operations. **g** Experimental confusion matrix and testing accuracy at 128 Gbaud. The 20-class classification accuracy is 95.4% and the 2-class classification accuracy is 100%.

The concept of NAD is shown in Fig. 3a. The objective is to classify whether network traffic originates from normal software or malware, enabling real-time intrusion detection in data centers. In this work, we implement a PNN to classify USTC-TFC2016 dataset^42^, which contains 10 classes of normal traffic and 10 classes of malware traffic. The PNN architecture is illustrated in Fig. 3b, which consists of an 8-channel photonic 1D convolution layer implemented by MRRs, followed by a digital FC layer. The input data stream is broadcast to eight channels and convolved by eight independent MRRs. For each MRR, the through and drop ports extract different temporal features from the same signal. The photodetector provides the intrinsic square-law nonlinearity. No additional digital activation function is used. Each channel output is defined as *y*_out_ = *y*_*T*_ − *y*_*D*_, where *y*_*T*_ and *y*_*D*_ are the through and drop port output, respectively. This can be implemented using a balanced photodetector, while it is performed digitally in our experiments. The eight output channels are then down-sampled by a factor of 3, flattened, and fed into a digital FC layer that outputs 20-class classification results (see Methods). The entire PNN, including both photonic convolution and digital FC layers, is trained end-to-end using backpropagation in PyTorch^43^. As shown in Fig. 3c, joint training of all layers significantly outperforms training only the FC layer.

Unlike conventional optical computing schemes, higher data rates in our architecture can lead to higher classification accuracy. We train and test the PNN at different data rates. As shown in Fig. 3d, when the symbol rate increases from 1 Gbaud to 128 Gbaud, the average testing accuracy improves from 93.3% to 96.4%. Further increasing the rate to 512 Gbaud causes a slight drop to 94.9%, indicating an optimal operating point around 128 Gbaud for this task.

Benefiting from the ultrahigh data rate, the MRR convolution engine can process approximately 160 million traffic samples per second. Fig. 3e shows experimental convolution results for 2 samples at 128 Gbaud, where each sample takes only 6.25 ns. The normalized root-mean-square error (NRMSE) between experimental data and the ground truth is 0.03 (see Supplementary Note 5). The zoomed waveforms in Fig. 3f reveal that the through and drop ports extract distinct temporal features at high speed, while at low speed (1 Gbaud) they degenerate to simple weighting operations. With 128 Gbaud operation, we experimentally achieve 95.4% accuracy for 20-class classification and 100% accuracy for binary (normal vs. malware) classification, demonstrating both high throughput and high accuracy. The confusion matrix is shown in Fig. 3g.

This result highlights a key conceptual advantage of our approach: in MRR-based temporal convolution, higher speed is not a burden but can instead become a benefit, directly translating into improved computational performance. This advantage is not limited to temporal sequence tasks such as NAD. We further show that an MRR-based PNN achieves higher accuracy at higher symbol rates in an MNIST^44^ image classification task. The details are provided in Supplementary Note 11.

### From O(*N*^2^) to O(*N*): hardware-efficient and high-throughput image convolution

By jointly exploiting SDM and WDM, the proposed MRR convolution engine supports multi-channel and high-dimensional convolution for image processing. In this section, we demonstrate that the MRR convolution engine can achieve higher computing throughput while requiring significantly fewer photonic devices for image classification.

**Fig. 4:**
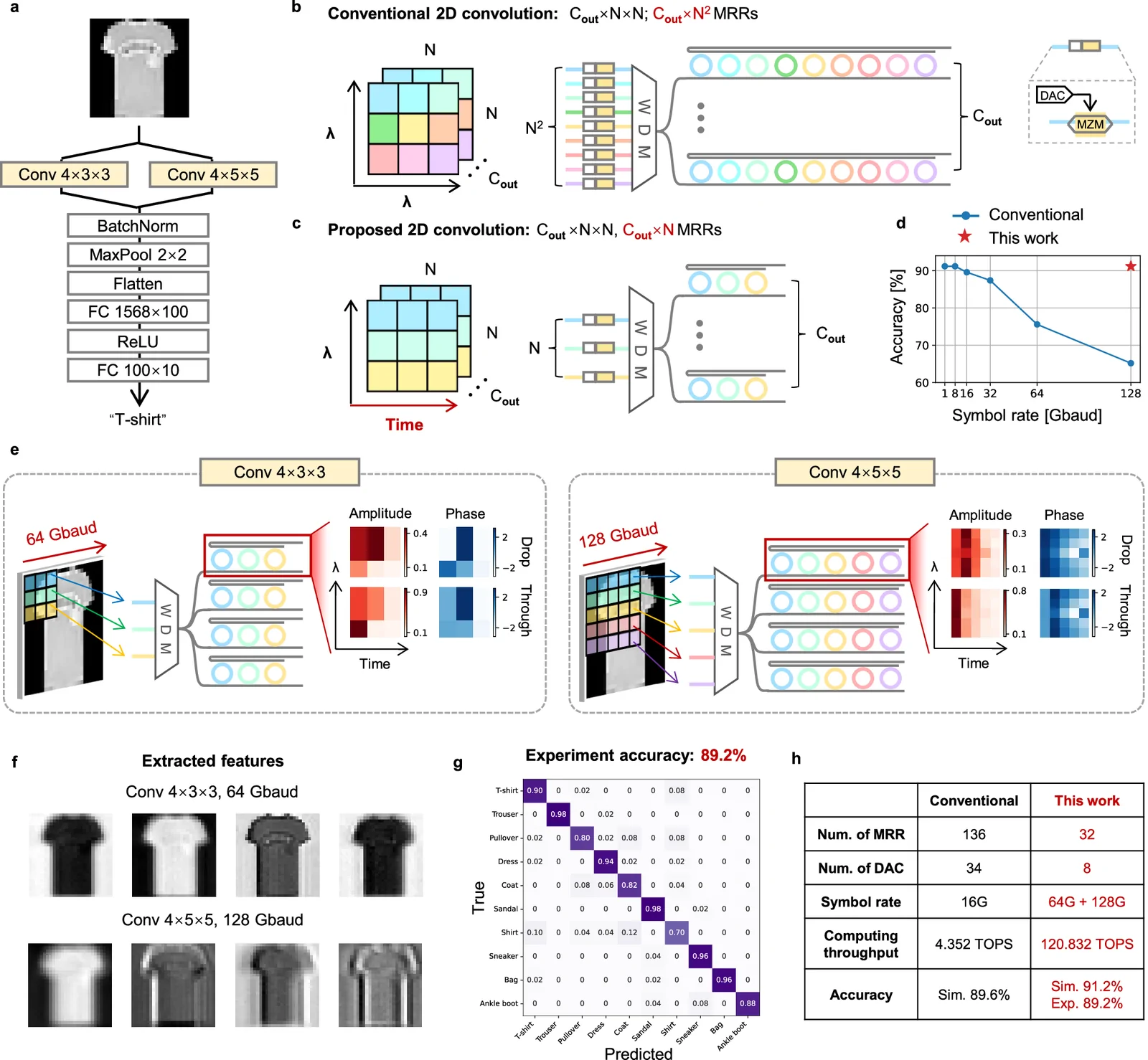
MRR 2D convolution for Fashion-MNIST classification. **a** Neural network design for Fashion-MNIST classification. It has 1 convolution layer and 2 FC layers. The convolution layer contains two types of kernels with different receptive fields, 3 × 3 and 5 × 5, each taking one input channel and producing four output channels. **b** Conventional scheme for 2D convolution, where each pixel of the convolution kernel is realized by an independent MRR for weighting. The hardware complexity for a *N* × *N* kernel is O(*N*^2^). **c** Proposed scheme for 2D convolution, which combines MRR temporal convolution and WDM. The hardware complexity for a *N* × *N* kernel is O(*N*). **d** Comparison of the classification accuracy of conventional scheme and the proposed scheme at different data rates in simulations. In conventional scheme, accuracy decreases from above 90% at 1 Gbaud to below 70% at 128 Gbaud. In the proposed scheme, the accuracy is 91.2% at 128 Gbaud. **e** Different kernel sizes are implemented by different symbol rates. A symbol rate of 64 Gbaud corresponds to an effective kernel size of 3, while 128 Gbaud corresponds to an effective kernel size of 5. **f** Examples of experimentally extracted feature maps. **g** Experimental results of testing accuracy (89.2%) and confusion matrix. **h** Comparison of the hardware requirements and computing performance of the conventional scheme and the proposed scheme. Our scheme provides a 27-fold increase in computing throughput together with a 4.25-fold reduction in hardware resources.

We implement a PNN for 10-class Fashion-MNIST^25^ image classification, as shown in Fig. 4a. The convolution layer contains two types of kernels with different receptive fields, 3 × 3 and 5 × 5, each taking one input channel and producing four output channels. These kernel configurations are designed in analogy to Inception-like architectures^45,46^, where multi-scale receptive fields improve feature extraction.

In conventional MRR-based photonic implementations of 2D convolution, each element in an *N* × *N* kernel is realized by an independent weighting element^20,27^. As illustrated in Fig. 4b, this requires *N*^2^ MRRs, together with *N*^2^ wavelength channels, modulators, drivers, and DACs. Such quadratic scaling leads to a heavy hardware burden and high energy consumption. Moreover, because each MRR is operated as a scalar weight, the symbol rate is constrained by the resonance linewidth. As a result, increasing the data rate significantly degrades the classification accuracy. This limitation is clearly shown in Fig. 4d, where the accuracy drops from above 90% to below 70% when the symbol rate increases from 1 Gbaud to 128 Gbaud in simulations. Consequently, both the achievable computing throughput and scalability are fundamentally limited.

In our scheme, one convolution dimension is mapped to the time domain and the other to the wavelength domain. Therefore, an *N* × *N* 2D convolution requires only *N* MRRs and *N* wavelengths, together with *N* modulators, drivers, and DACs, as shown in Fig. 4c. This reduces the hardware complexity of a convolution kernel from O(*N*^2^) to O(*N*). At the same time, the architecture supports ultrahigh symbol rates up to 128 Gbaud, enabling a dramatic increase in computing throughput. As shown in Fig. 4d, the proposed PNN achieves a classification accuracy of 91.2% at 128 Gbaud in simulation, demonstrating that the hardware complexity reduction and high-speed operation do not degrade performance but coexist with high accuracy. The proposed architecture can be further extended to higher dimensional convolution, as discussed in Supplementary Note 6.

Figure 4e illustrates how different kernel sizes are implemented in our system. Because the impulse response of an MRR decays in time, the effective length of the temporal convolution kernel increases with the symbol rate. For the MRRs used in our chip, a symbol rate of 64 Gbaud per MRR corresponds to an effective kernel size of 3, while 128 Gbaud corresponds to an effective kernel size of 5 (see Supplementary Note 2). In this way, the receptive field of the convolution kernel can be programmed directly by adjusting the data rate, without modifying any physical hardware. Each MRR generates two feature maps through its through and drop ports. In our implementation, the output of each row is defined as *y* = *w*_T_*y*_T_ + *w*_D_*y*_D_, where *y*_T_ and *y*_D_ are the signals from the through and drop ports, and *w*_T_ and *w*_D_ are trainable weights. This operation can be implemented digitally, as in our experiment, or optically by inserting a Mach–Zehnder interferometer (MZI) before the balanced photodetector to independently control the amplitudes of the two ports (see Supplementary Note 7 for details).

Examples of experimentally extracted feature maps are shown in Fig. 4f. We achieve an experimental classification accuracy of 89.2%, slightly lower than the simulated accuracy of 91.2%, mainly due to experimental noise. The corresponding confusion matrix is shown in Fig. 4g.

Finally, we compare the hardware requirements and computing performance of our method with conventional MRR-based convolution schemes. As summarized in Fig. 4h, the conventional approach requires 136 MRRs and 34 DACs. To maintain classification accuracy of around 90% in simulation (Fig. 4d), we assume it can operate at 16 Gbaud, which provides a computing throughput of 4.352 TOPS. In contrast, our architecture uses only 32 MRRs and 8 DACs, supports symbol rates up to 128 Gbaud, and achieves a computing throughput of 120.832 TOPS (see Methods). This corresponds to a 27-fold increase in computing throughput together with a 4.25-fold reduction in hardware resources, without sacrificing classification accuracy.

These results demonstrate that by combining the MRR temporal convolution kernel with WDM and SDM, the proposed MRR convolution architecture simultaneously achieves high computational throughput, low hardware complexity, and flexible kernel reconfigurability, making it highly attractive for large-scale PNNs.

## Discussion

### Computational role of MRR temporal kernels

By operating the system at a symbol rate exceeding the MRR resonance linewidth, we intentionally introduce temporal overlaps of neighboring symbols in the signals. In optical communication systems and conventional weight-based photonic computing systems, such overlap is known as inter-symbol interference, which is typically uncontrollable and regarded as an unwanted impairment that degrades symbol-level fidelity. However, in the proposed architecture, this effect becomes programmable and trainable computational operation induced by the MRR responses. It functions as a convolution operation in the PNNs, enabling the extraction of task-relevant features and contributing substantially to the network performance after training. Further discussion is detailed in Supplementary Note 10.

To evaluate the effectiveness of MRR temporal kernels, we compare the classification accuracy of the proposed PNNs with that of several digital neural networks, as detailed in Supplementary Note 9. The results show that the PNNs with MRR-based convolutional layers consistently outperform both the FC-only network and the parameter-matched digital CNN, in which the number of trainable convolutional parameters is matched to the number of MRRs, across all demonstrated tasks. Moreover, for the MFI and Fashion-MNIST classification tasks, the PNNs achieve accuracy comparable to that of significantly larger digital CNNs, indicating that the MRR-based convolutional layers can perform convolution operations with high parameter efficiency.

In addition, the MRR temporal kernels themselves can accomplish certain neural network tasks without the FC layers. A proof-of-concept demonstration using only the MRR convolution layer for image classification is provided in Supplementary Note 19.

### Compute density and energy efficiency

The proposed MRR convolution architecture achieves high computing throughput and high compute density not only by supporting ultrafast data rates, but more fundamentally because each MRR functions as a convolution kernel rather than a single scalar weight. At a data rate of 128 Gbaud, one MRR works as a complex-valued 1 × 5 convolution kernel, providing a computing throughput of 5.12 TOPS. In this work, an 8 × 8 MRR array has a footprint of 3.1 × 2.2 mm^2^, yielding a compute density of 48.05 TOPS/mm^2^ at 128 Gbaud. The current chip area is dominated by electrical routes and pads rather than by the photonic devices themselves. These overheads can be significantly reduced by adopting advanced packaging techniques. Using a recently demonstrated 3D photonic integration technique^47^, the compute density can be improved to 2730.67 TOPS/mm^2^ (see Methods). The scale of the MRR array can be further extended. A 16 × 16 MRR array has been demonstrated in previous work^48^. When evaluated using our convolution-based paradigm, it can provide a total computing throughput of 1310.72 TOPS.

The power consumption of high-speed optical computing systems is usually dominated by high-speed DACs and ADCs. The proposed convolution architecture achieves higher energy efficiency than conventional schemes because it performs more computing operations per DAC. Considering an *N* × *N* convolution, the conventional scheme requires *N*^2^ DACs. The total number of computing operations within one DAC conversion time step is 2*N*^2^ (including *N*^2^ real-value multiplications and *N*^2^ summations). Therefore, the number of computing operations per DAC conversion is 2 in the conventional scheme. In the proposed scheme, an *N* × *N* convolution requires only *N* DACs. Additionally, our scheme performs complex-valued convolution at both through and drop ports, resulting in a total of 8*N*^2^ computing operations within one DAC conversion time step. Therefore, the number of computing operations per DAC conversion is 8*N* in this work. Taking *N* = 5, our scheme shows 20 × improvement in computing operations per DAC conversion.

With the demonstrated computing throughput of 120.832 TOPS in the Fashion-MNIST classification task, the system-level energy efficiency is about 26.7 TOPS/W, where the power consumption of on-chip heaters, lasers, modulators, photodetectors, DACs, and ADCs are all included (see Supplementary Note 8 for details). The energy efficiency can be further improved by scaling up the MRR arrays, because for an *N* × *N* MRR array, the computing throughput scales as O(*N*^2^) while the number of DACs and ADCs scales as only O(*N*). A 16 × 16 MRR array evaluated using the proposed computing paradigm is estimated to achieve an energy efficiency of 152.8 TOPS/W.

Table 1 compares our approach with representative MRR-based computing architectures. Thanks to the combination of ultrahigh symbol rate, kernel-level computation of each MRR, and multiple computing operations per DAC conversion, our architecture achieves orders-of-magnitude improvement in computing throughput, compute density, and energy efficiency.

**Table 1:** Comparison with representative MRR computing architectures

References	Function of MRR	Symbol rate (Gbaud)	Per-MRR computing throughput (TOPS)	Total computing throughput (TOPS)	Compute density (TOPS/mm^2^)	Energy efficiency (TOPS/W)
Ref. ^15^	Weight	20	0.04	0.48	0.588	/
Ref. ^16^	Weight	17	0.034	0.136	1.04	0.2
Ref. ^17^	Weight	22.38	0.045	0.537	1.37	/
Ref. ^18^	Weight	10	0.08	1.28	/	/
Ref. ^13^	Weight	30.67	0.245	0.245	34.04^a^	/
Ref. ^19^	Weight	5	0.01	0.04	/	/
Ref. ^20^	Modulation and weight	9.83	0.0196	0.196	4.67	/
Ref. ^21^	Weight	30	0.06	0.96	0.0245	0.3
This work	Convolution	128	5.12	120.832	48.05	26.7
This work projected	Convolution	128	5.12	1310.72	2730.67	152.8

^a^Only accounts for the footprint of one MRR.

### Robustness and stability

Photonic integrated circuits face practical challenges arising from fabrication variations and thermal crosstalk effects. Nevertheless, by employing the chip-in-the-loop method, all MRRs can be programmed to their target resonance detuning states with high precision, achieving a residual frequency offset of about 0.1 GHz (see Supplementary Note 14). Discrepancies may still exist between the actual MRR impulse responses and their digital models due to fabrication-induced variations in FWHM bandwidth and residual tuning errors. To address this problem, we employ sharpness-aware training^36^ to improve the robustness of PNNs against these imperfections. For the three tasks demonstrated, the PNNs maintain their accuracy under fabrication variations and detuning errors that are twice as large as those observed experimentally (see Supplementary Note 15). In addition, by incorporating noise and timing errors during training, the PNNs can work accurately and stably in the presence of noise and timing jitter in the high-speed analog computing system (see Supplementary Notes 16 and 17).

Stability is another important metric in analog computing systems. To evaluate long-term operational accuracy and stability, we continuously performed the NAD task over a period of 5 h. The classification accuracy remained stable at approximately 95% throughout the experiment, without any noticeable performance degradation (see Supplementary Note 1^8^).

### Versatile application expansion

The proposed MRR temporal convolution kernels serve as a versatile computational primitive for photonic computing. Beyond the classification tasks, we further investigate their applicability to optical communication signal processing and nonlinear time-series prediction through numerical simulations.

For optical communication signal processing, we demonstrate using an MRR-based photonic equalizer for 120 Gbaud PAM4 signal across 80-km single-mode fiber transmission at C-band. Using 2 MRR temporal kernels and 2 × 161-tap feed-forward equalizer (FFE), the bit error rate (BER) is 8.09 × 10^−4^, far below the hard-decision forward-error-correction (HD-FEC) threshold of 3.8 × 10^−3^. For comparison, a digital convolutional neural network containing over 38 × trainable parameters achieves a BER of 9.21 × 10^−2^, substantially above the HD-FEC limit. The superior performance of the MRR-based equalizer arises from its ability to perform complex-valued convolution directly on complex optical fields prior to photodetection^49^, thereby preserving phase information that is inaccessible to conventional intensity-domain DSP approaches. More details are provided in Supplementary Note 20.

We further evaluate the capability of the MRR temporal kernel for nonlinear time-series prediction. For predicting Santa Fe Laser data^50^, PNN based on the MRR temporal kernels achieves a normalized mean-square error (NMSE) of 0.012 when operating at 128 Gbaud. In contrast, when operating at 1 Gbaud, MRRs degenerate to scalar weighting elements and result in NMSE of 0.069. More details are provided in Supplementary Note 21. These results highlight the strong temporal feature-extraction capability of the proposed MRR kernels and demonstrate their effectiveness beyond classification tasks.

### Degrees of freedom and future extensions

In the proposed MRR convolution architecture, though the number of trainable parameters is smaller than the DOF of fully arbitrary convolution kernels, the trained kernels achieve classification accuracy comparable to that obtained using arbitrary kernels. This situation is conceptually similar to model compression and structured parameterization in neural networks, where a reduced parameter space is intentionally imposed to improve hardware efficiency without sacrificing performance^51–53^. In our case, although each MRR kernel is parameterized by a limited set of physical variables (primarily the resonance frequency), the end-to-end training enables the network to adapt to this constrained kernel space and extract task-relevant features efficiently.

The DOF of the MRR convolution engine can be further increased by reconfiguring the resonant line shape, such as the FWHM and the extinction ratio. Physically, this can be achieved by introducing tunable coupling coefficients between the MRR and the bus waveguides, such as using MZI-based couplers^54–56^. Such extensions would enable more flexible kernel shaping while still preserving its intrinsic advantages.

In conclusion, we have proposed and experimentally demonstrated ultrafast photonic convolution neural networks based on MRRs, where each MRR acts as a temporal convolution kernel rather than a scalar weight. This paradigm fundamentally enhances computing throughput and compute density by providing ultrahigh data rates and multiple multiplication-accumulation computing capacity in single devices. We experimentally validated the effectiveness of this architecture through three representative tasks: MFI requiring only one MRR, NAD showing that higher speed leads to higher accuracy, and Fashion-MNIST image classification demonstrating high computing throughput with significantly reduced hardware resources. These results indicate that MRR-based temporal convolution offers a promising pathway toward scalable, energy-efficient, and high-performance PNNs for next-generation artificial intelligence.

## Methods

### Device fabrication

The photonic chip is fabricated on the silicon-on-insulator (SOI) platform with a 220-nm-thick silicon device layer and 2.2 μm-thick oxide cladding. The MRR array consists of 64 MRRs arranged as 8 rows. Each row has 8 MRRs with radii ranging from 15 to 15.08 μm, coupled with 2 bus waveguides in an add/drop configuration. The gap between MRRs and bus waveguides is 0.1 μm. The Q factor of MRR is about 10^4^. Light is coupled into and out of the chip through TE-mode grating couplers, with around −7dB coupling loss each side. Titanium-tungsten (TiW) alloy heater is built on top of each MRR for thermal tuning. The heater’s resistance is around 200 ohm. The tuning efficiency of MRR is about 0.056 nm/mW. The chip is packaged with an optical fiber array, electrical wire-bonding, and a thermoelectric cooler (TEC).

### Experimental setup

A tunable laser (TLS, Keysight N7778C) is used as optical source. For MFI and NAD, the working wavelength is 1550 nm. For Fashion-MNIST image classification, the wavelength ranges from 1550 to 1553.2 nm with interval of 0.8 nm. High-speed signals are generated by an arbitrary waveform generator (AWG, Keysight M8199A) at 256 GSa/s, driven by an electrical amplifier (EA, SHF 804C), and modulated by a 90-GHz commercial TFLN intensity modulator (Liobate LN-90). A polarization controller is used to align the polarization and minimize coupling loss before coupling light into the on-chip waveguide. All on-chip heaters are programmed by a multi-channel source-meter (Silicon Extreme MSVS6400-A). A TEC control module is used to stabilize temperature at 22 °C. After the chip, an erbium-doped fiber amplifier (EDFA) is used to amplify the optical signal, followed by a tunable optical band-pass-filter with 100 GHz bandwidth to reduce out-of-band amplified spontaneous emission noise. After a variable optical attenuator (VOA) to control the received optical power, the optical signal is detected by a commercial 70-GHz photodetector (Coherent XPDV3120R). The received electrical signal is recorded by a real-time oscilloscope (RTO, Keysight UXR0702AP) at 256 GSa/s.

### Dataset preparation and neural network design

#### Modulation format identification

The modulation format identification task involves 4 classes: OOK at 32 Gbaud, OOK at 40 Gbaud, PAM4 at 32 Gbaud, and PAM4 at 40 Gbaud. The dataset is generated using Python, with each class containing 100 training samples and 100 testing samples. Each sample consists of 4096 randomly generated symbols, which are shaped by a root-raised-cosine (RRC) filter with a roll-off factor of 0.1 and 64 taps. The signals are then upsampled to 256 GSa/s to match the sampling rate of the AWG. The experimentally acquired data from the RTO are downsampled to 50 GSa/s without synchronization in order to emulate asynchronous sampling conditions. After normalizing the signal amplitudes to the range [0, 1], a 10-bin AAH is constructed for each sample. Finally, an FC layer of size 10  × 4 is employed to produce the classification outputs.

#### Network anomaly detection

For the network anomaly detection task, we use the USTC-TFC2016^42^ dataset, which contains 20 classes of one-dimensional network traffic data. Among these, 10 classes correspond to benign software, while the remaining 10 classes represent malware. The dataset comprises 245,437 training samples and 27,271 testing samples. The entire training set is used. A subset of 1000 samples is randomly selected from the testing set for experimental inference, with 50 samples per class. Each original sample has a length of 784. To mitigate inter-sample interference caused by temporal convolution effects, eight zeros are padded at both the beginning and the end of each sample, resulting in a total length of 800. At a data rate of 128 Gbaud, each sample occupies 6.25 ns in the time domain. We design a CNN consisting of an eight-channel MRR convolution layer followed by a single FC layer for classification. The input signals are first processed by the photonic chip and then captured by the RTO. For each channel, the zero-padding is removed, and the signal is downsampled by a factor of three, yielding a length of 261 per channel. After flattening all eight channels, the resulting feature vector has a total length of 2088. A 2088  × 20 FC layer is then applied to generate the final classification results.

#### Fashion-MNIST image classification

The Fashion-MNIST^25^ dataset consists of 70,000 grayscale images of fashion products, each with a resolution of 28 × 28 pixels, categorized into 10 classes. The dataset includes 60,000 training images and 10,000 test images. The full training set is used, while a subset of 500 images is randomly selected from the testing set for experimental inference, with 50 samples per class. The neural network architecture comprises one convolutional layer followed by two FC layers, as illustrated in Fig. 4(a).

### Neural network training

All neural networks are trained in silico using PyTorch^43^. A differentiable model of the MRR is required for backpropagation during training. In this work, the MRR is modeled in the frequency domain using Eqs. (1) and (2), with parameters such as the coupling coefficient and round-trip attenuation factor extracted from the experimentally measured transmission spectrum.

Within the convolution layer, the input signal is first transformed into the frequency domain via the Fast Fourier transform (FFT), filtered using the frequency response of the MRR, and then converted back to the time domain using the inverse FFT (IFFT). To emulate noise present in the physical analog system, noise-injection is applied during the forward pass of training. In addition, bandwidth limitations are incorporated into the model to reduce the discrepancy between the digital simulation and the physical system.

Furthermore, sharpness-aware training (SAT)^36^ is employed to enhance the robustness of the PNN. The SAT optimization objective is defined as minimizing the following loss function *L*_1_ with respect to the trainable parameter vector ***θ***:

7 $${L}_{1}=L({{{\bf{y}}}},{{{{\bf{y}}}}}_{{{{\rm{target}}}}};{{{\boldsymbol{\theta }}}})+\alpha {\left\Vert \frac{\partial L({{{\bf{y}}}},{{{{\bf{y}}}}}_{{{{\rm{target}}}}};{{{\boldsymbol{\theta }}}})}{\partial {{{\boldsymbol{\theta }}}}}\right\Vert }_{2}.$$

where **y** denotes the system output vector, **y**_target_ is the target output vector, and *α* is a weighting factor. The trainable parameter vector is denoted by ***θ***. The first term seeks to minimize the empirical loss, while the second term penalizes large gradients, thereby reducing the sensitivity of the loss function to parameter perturbations and improving robustness against physical imperfections.

### Performance evaluation

#### Computing throughput

In the conventional scheme where MRRs work as scalar weights, the computing throughput of each MRR is calculated as Comp_weight_ = 2 × *B*. For a signal rate of 16 Gbaud, Comp_weight_ = 32 GOPS. In our scheme, the computing throughput of each MRR convolution kernel is calculated as Comp_conv_ = *K* × *B* × 2 × 2 × 2, where *K* is the kernel size, *B* is the baud rate, and the three factors of 2 account for complex-valued signals, multiply–accumulate operations, and through/drop ports, respectively. For signals operating at 128 (64) Gbaud, the effective kernel sizes are estimated to be 5 (3), resulting in a computing throughput of 5.12 (1.536) TOPS per MRR. Therefore, the computing throughput of each MRR is increased by up to 160 × . In the Fashion-MNIST image classification task, 20 MRRs operate at 128 Gbaud and 12 MRRs operate at 64 Gbaud, yielding a total computing throughput of 120.832 TOPS. Using the same estimation method, we project that a 16 × 16 MRR array operating at 128 Gbaud could achieve a computing throughput of 1310.72 TOPS.

#### Compute density

Compute density is defined as computing throughput divided by chip footprint. The computing throughput of an MRR is 5.12 TOPS at 128 Gbaud. For the chip used in this work, an 8 × 8 MRR array has a footprint of 3.1 × 2.2 mm^2^, yielding a compute density of 48.05 TOPS/mm^2^ at 128 Gbaud. Recently, a 3D-packaged photonic chip integrating 80 MRRs within a footprint of 0.15 mm^2^ has been demonstrated^47^. Based on this level of integration, we project that a 3D-packaged implementation could achieve a compute density as high as 2730.67 TOPS/mm^2^ at 128 Gbaud.

#### Energy efficiency

Energy efficiency is defined as computing throughput divided by power consumption. The system power consumption is calculated as *P*_system_ = *P*_heater_ + *P*_laser_ + *P*_modulator_ + *P*_PD_ + *P*_DAC_ + *P*_ADC_. In the Fashion-MNIST classification task, the system employs 32 MRRs, 8 lasers, 8 intensity modulators, 8 photodetectors, 8 DACs, and 8 ADCs, leading to total power consumption of 4528 mW. With a computing throughput of 120.832 TOPS, the resulting energy efficiency is approximately 26.7 TOPS/W. We project a 16 × 16 MRR array working at 128 Gbaud can achieve an energy efficiency of 152.8 TOPS/W. The details are provided in Supplementary Note 8.

## Supplementary information

Transparent Peer Review file

Supplementary information

## Data Availability

The data that generates figures in this paper is available on Zenodo^57^.

## References

[1] Sevilla, J. et al. Can AI scaling continue through 2030? https://epoch.ai/blog/can-ai-scaling-continue-through-2030/ (2024).

[2] Waldrop, M. M. The chips are down for Moore’s law. *Nat. N.* **530**, 144 10.1038/530144a (2016). 26863965

[3] Mehonic, A. & Kenyon, A. J. Brain-inspired computing needs a master plan. *Nature* **604**, 255–260 10.1038/s41586-021-04362-w (2022). 35418630

[4] Wang, S. et al. Technology roadmap of bioinspired computing hardware. *ACS Nano*10.1021/acsnano.5c17087 (2026). 41771048

[5] Tait, A. N. et al. Neuromorphic photonic networks using silicon photonic weight banks. *Sci. Rep.* **7**, 7430 10.1038/s41598-017-07754-z (2017). PMC5547135 28784997

[6] Shen, Y. et al. Deep learning with coherent nanophotonic circuits. *Nat. Photonics* **11**, 441–446 10.1038/nphoton.2017.93 (2017).

[7] Feldmann, J. et al. Parallel convolutional processing using an integrated photonic tensor core. *Nature* **589**, 52–58 10.1038/s41586-020-03070-1 (2021). 33408373

[8] Huang, C. et al. A silicon photonic–electronic neural network for fibre nonlinearity compensation. *Nat. Electron.* **4**, 837–844 10.1038/s41928-021-00661-2 (2021).

[9] Huang, C. et al. Prospects and applications of photonic neural networks. *Adv. Phys. X* **7**, 1981155 10.1080/23746149.2021.1981155 (2022).

[10] Zhou, H. et al. Photonic matrix multiplication lights up photonic accelerator and beyond. *Light Sci. Appl.* **11**, 30 10.1038/s41377-022-00717-8 (2022). PMC8814250 35115497

[11] Xu, Z. et al. Large-scale photonic chiplet Taichi empowers 160-TOPS/W artificial general intelligence. *Science* **384**, 202–209 10.1126/science.adl1203 (2024). 38603505

[12] Feng, H. et al. Integrated lithium niobate microwave photonic processing engine. *Nature* **627**, 80–87 10.1038/s41586-024-07078-9 (2024). 38418888

[13] Meng, X. et al. High-integrated photonic tensor core utilizing high-dimensional lightwave and microwave multidomain multiplexing. *Light Sci. Appl.* **14**, 27 10.1038/s41377-024-01706-9 (2025). PMC11697043 39746909

[14] Tait, A. N. et al. Microring weight banks. *IEEE J. Sel. Top. Quantum Electron.* **22**, 312–325 10.1109/JSTQE.2016.2573583 (2016).

[15] Xu, S., Wang, J., Yi, S. & Zou, W. High-order tensor flow processing using integrated photonic circuits. *Nat. Commun.* **13**, 7970 10.1038/s41467-022-35723-2 (2022). PMC9797566 36577748

[16] Bai, B. et al. Microcomb-based integrated photonic processing unit. *Nat. Commun.* **14**, 66 10.1038/s41467-022-35506-9 (2023). PMC9814295 36604409

[17] Yin, R. et al. Integrated WDM-compatible optical mode division multiplexing neural network accelerator. *Optica* **10**, 1709–1718 10.1364/OPTICA.500523 (2023).

[18] Jiang, Y. et al. Photonic 4D-convolution tensor core based on micro-ring mesh architecture. In: Optical Fiber Communication Conference (OFC) 2025 (2025), Paper W3D.3, pp. 3–3. Optica Publishing Group 10.1364/OFC.2025.W3D.3 (2025).

[19] Khaled, A., Aadhi, A., Huang, C., Tait, A. N. & Shastri, B. J. Fully integrated hybrid multimode-multiwavelength photonic processor with picosecond latency. *Nat. Commun.* **17**, 28 10.1038/s41467-025-66561-7 (2025). PMC12764859 41413380

[20] Zhang, W., Wang, Y., Lederman, J. C., Shastri, B. J. & Prucnal, P. R. Compact, reconfigurable, and scalable photonic neurons by modulation-and-weighting microring resonators. *eLight* **6**, 6 10.1186/s43593-026-00122-3 (2026). PMC12886301 41675197

[21] Wu, Y. et al. Integrated photonic 3D tensor processing engine. *Light Sci. Appl.* **15**, 154 10.1038/s41377-026-02183-y (2026). PMC12966298 41792110

[22] Liu, F. et al. Compact optical temporal differentiator based on silicon microring resonator. *Opt. Express* **16**, 15880 10.1364/OE.16.015880 (2008). 18825224

[23] Ferrera, M. et al. On-chip CMOS-compatible all-optical integrator. *Nat. Commun.* **1**, 29 10.1038/ncomms1028 (2010). PMC2982162 20975692

[24] Tsirigotis, A. et al. Photonic neuromorphic accelerator for convolutional neural networks based on an integrated reconfigurable mesh. *Commun. Eng.* **4**, 80 10.1038/s44172-025-00416-3 (2025). PMC12038015 40295731

[25] Xiao, H., Rasul, K. & Vollgraf, R. Fashion-MNIST: a novel image dataset for benchmarking machine learning algorithms. Preprint at 10.48550/arXiv.1708.07747 (2017).

[26] Van, V. *Optical microring resonators: theory, techniques, and applications* 10.1201/9781315303512 (CRC Press, 2016).

[27] Liu, S. et al. Calibration-free and precise programming of large-scale ring resonator circuits. *Optica* **12**, 1113–1121 10.1364/OPTICA.557415 (2025).

[28] Rumelhart, D. E., Hinton, G. E. & Williams, R. J. Learning representations by back-propagating errors. *Nature* **323**, 533–536 10.1038/323533a0 (1986).

[29] Appeltant, L. et al. Information processing using a single dynamical node as complex system. *Nat. Commun.* **2**, 468 10.1038/ncomms1476 (2011). PMC3195233 21915110

[30] Coarer, F. D.-L. et al. All-optical reservoir computing on a photonic chip using silicon-based ring resonators. *IEEE J. Sel. Top. Quantum Electron.* **24**, 1–8 10.1109/JSTQE.2018.2836985 (2018).

[31] Wang, D., Nie, Y., Hu, G., Tsang, H. K. & Huang, C. Ultrafast silicon photonic reservoir computing engine delivering over 200 TOPS. *Nat. Commun.* **15**, 10841 10.1038/s41467-024-55172-3 (2024). PMC11686264 39738199

[32] Moralis-Pegios, M. et al. Neuromorphic silicon photonics and hardware-aware deep learning for high-speed inference. *J. Lightwave Technol.* **40**, 3243–3254 (2022).

[33] Kariyappa, S. et al. Noise-resilient DNN: tolerating noise in PCM-based AI accelerators via noise-aware training. *IEEE Trans. Electron Devices* **68**, 4356–4362 10.1109/TED.2021.3089987 (2021).

[34] Mourgias-Alexandris, G. et al. Noise-resilient and high-speed deep learning with coherent silicon photonics. *Nat. Commun.* **13**, 5572 10.1038/s41467-022-33259-z (2022). PMC9508134 36151214

[35] Wu, C. et al. Harnessing optoelectronic noises in a photonic generative network. *Sci. Adv.* **8**, 2956 10.1126/sciadv.abm2956 (2022). PMC8782447 35061531

[36] Xu, T. et al. Physical neural networks using sharpness-aware training. *Nat. Commun.* **17**, 1766 10.1038/s41467-026-68470-9 (2026). PMC12917175 41554739

[37] Saif, W. S. et al. Machine learning techniques for optical performance monitoring and modulation format identification: a survey. *IEEE Commun. Surv. Tutor.* **22**, 2839–2882 10.1109/COMST.2020.3018494 (2020).

[38] Khan, F. N., Zhou, Y., Lau, A. P. T. & Lu, C. Modulation format identification in heterogeneous fiber-optic networks using artificial neural networks. *Opt. Express* **20**, 12422–12431 10.1364/OE.20.012422 (2012). 22714229

[39] Wang, D. et al. Intelligent constellation diagram analyzer using convolutional neural network-based deep learning. *Opt. Express* **25**, 17150–17166 10.1364/OE.25.017150 (2017). 28789210

[40] Krizhevsky, A., Sutskever, I. & Hinton, G.E. ImageNet classification with deep convolutional neural networks. In *Advances in Neural Information Processing Systems* Vol. 25 (Curran Associates, Inc, 2012).

[41] Ahmed, M., Naser Mahmood, A. & Hu, J. A survey of network anomaly detection techniques. *J. Netw. Comput. Appl.* **60**, 19–31 10.1016/j.jnca.2015.11.016 (2016).

[42] Wang, W., Zhu, M., Zeng, X., Ye, X. & Sheng, Y. Malware traffic classification using convolutional neural network for representation learning. In *2017 International Conference on Information Networking (ICOIN)*, 712–717 10.1109/ICOIN.2017.7899588 (2012).

[43] Paszke, A. et al. PyTorch: an imperative style, high-performance deep learning library. In *Advances in Neural Information Processing Systems*, Vol. 32 (Curran Associates Inc, 2019)

[44] Lecun, Y., Bottou, L., Bengio, Y. & Haffner, P. Gradient-based learning applied to document recognition. *Proc. IEEE* **86**, 2278–2324 10.1109/5.726791 (1998).

[45] Szegedy, C. et al. Going deeper with convolutions. In *Proc. IEEE Conference on Computer Vision and Pattern Recognition*, pp. 1–9 (IEEE, 2015)

[46] Cai, X. et al. Poly kernel inception network for remote sensing detection. In *Proc. IEEE/CVF Conference on Computer Vision and Pattern Recognition* 27706–27716 (IEEE, 2024)

[47] Daudlin, S. et al. Three-dimensional photonic integration for ultra-low-energy, high-bandwidth interchip data links. *Nat. Photonics* **19**, 502–509 10.1038/s41566-025-01633-0 (2025).

[48] Jayatilleka, H., Shoman, H., Chrostowski, L. & Shekhar, S. Photoconductive heaters enable control of large-scale silicon photonic ring resonator circuits. *Optica* **6**, 84–91 10.1364/OPTICA.6.000084 (2019).

[49] Wang, B. et al. An all-optical signal processor enabling terabit-per-second real-time equalization. *Science* **392**, 5344 10.1126/science.ady5344 (2026). 42275501

[50] Weigend, A.S. & Gershenfeld, N.A. Results of the time series prediction competition at the Santa Fe Institute. In *IEEE International Conference on Neural Networks* 1786–17933 10.1109/ICNN.1993.298828 (IEEE, 1993).

[51] Jaderberg, M., Vedaldi, A. & Zisserman, A. Speeding up convolutional neural networks with low rank expansions. Preprint at 10.48550/arXiv.1405.3866 (2014).

[52] Ding, C. et al. CirCNN: accelerating and compressing deep neural networks using block-circulant weight matrices. In *Proc. 50th Annual IEEE/ACM International Symposium on Microarchitecture* 395–408. Association for Computing Machinery 10.1145/3123939.3124552 (IEEE, 2017).

[53] Cheng, Y., Wang, D., Zhou, P. & Zhang, T. A survey of model compression and acceleration for deep neural networks. Preprint at 10.48550/arXiv.1710.09282 (2020).

[54] Shen, H. et al. Eight-channel reconfigurable microring filters with tunable frequency, extinction ratio and bandwidth. *Opt. Express* **18**, 18067–18076 10.1364/OE.18.018067 (2010). 20721194

[55] Zhao, L., Li, S., Ben, D. & Pan, S. Tuning of transmission responses of an optical microring with negligible wavelength shift. *IEEE Photonics Technol. Lett.* **30**, 227–230 10.1109/LPT.2017.2778342 (2018).

[56] Sun, Y. et al. Field-programmable ring array employing AMZI-assisted-MRR structure for photonic signal processor. *APL Photonics* **9** 10.1063/5.0209603 (2024).

[57] Liu, S. et al. Data for: “Microrings as programmable temporal kernels enabling photonic AI beyond 100 Gbaud”. Zenodo. 10.5281/zenodo.21801209 (2026). PMC13696697 42928223

