# Supplementary material for: Microrings as programmable temporal kernels enabling photonic AI beyond 100 Gbaud
Source: Nat Commun. 2026 Sep 25;17:11107. doi: 10.1038/s41467-026-77446-8 (PMC13696697; doi:10.1038/s41467-026-77446-8)
Supplement: Supplementary file 1 [file 41467_2026_77446_MOESM1_ESM.pdf]

nature portfolio

# Peer Review File

# Microrings as programmable temporal kernels enabling photonic AI beyond 100 Gbaud

Corresponding Author: Professor Chaoran Huang

**This file contains all reviewer reports in order by version, followed by all author rebuttals in order by version.**

Version 0:

Reviewer comments:

Reviewer #1

(Remarks to the Author)
In the manuscript, the authors introduce a regime to use MRR as a temporal convolutional kernel instead of a scalar weighting element, achieving much faster operating throughput. The idea is straightforward and the implementation is simple without major revisions to the traditional MRR-based photonic matrix-vector-multipliers. Solid experimental results are also well presented to proof the effectiveness of this approach. With that said, I would make any recommendation after those comments in the below are addressed.

1) Although strict and detailed mathematically expression of the MRR's temporal response are given, there lack experimental measurements of the actual temporal responses of those MRRs on the PIC. It looks like the kernel depends on both the detuning and the FWHM of the MRR. The detuning can be off over by the temperature drift and the FWHMs can also be off from the designed value by both environmental drift and fabrication error. So I would like to see the actual measured kernels and their comparison with the authors' presented theoretical analysis.

2) All the three presented tasks utilize not only the convolution layers that implemented on the PIC but also, more or fewer, some FC layers and nonlinear functions that implemented not on the PIC (maybe on a computer). I wonder how much does the PIC contribute to the overall network performance? If possible, the authors can compare the results of the networks with those convolutional layers implemented digitally and that on implemented PIC. In such a way, we can accuracy can the PIC carry out the convolutional layers.

3) Is it possible to show a task that can be fully implemented on the PIC only without any use of other digital layers?

Reviewer #2

(Remarks to the Author)
This manuscript presents a paradigm shift in photonic computing by redefining microring resonators as programmable temporal convolution kernels rather than scalar weights. The core innovation—exploiting the LTI filter characteristics of MRRs when signal bandwidth exceeds resonance linewidth (~20 GHz) to perform time-domain convolution—is theoretically sound and experimentally validated across three tasks (MFI, NAD, and Fashion-MNIST). The approach elegantly transforms the traditional "bandwidth limitation" into a computational resource.
However, a critical clarification is required regarding the manuscript's central claim of operation "beyond 100 GHz." Supplementary Figure S3c indicates the experimental system has a 3-dB bandwidth of only ~38 GHz, while the MRR linewidth is ~20 GHz. The claimed "128 Gbaud" operation refers to the symbol rate (enabled by training-based compensation of severe inter-symbol interference in the band-limited channel), not analog bandwidth exceeding 100 GHz. This distinction is crucial for scientific accuracy and must be explicitly addressed to avoid misleading the photonics and signal processing communities.
With revisions clarifying the bandwidth constraints, transparent discussion of electronic I/O dominance in energy consumption, and additional characterization of thermal effects, this work represents a significant contribution to photonic AI accelerators suitable for Nature Communications:
1. When comparing with electronic accelerators or other photonic systems, the system-level energy (including DAC/ADC) must be used, not just the photonic chip consumption. Comparison with other works may be unfair if those works use different DAC/ADC schemes. Please state clearly in the main text that DAC/ADC dominate power consumption and discuss

implications for practical deployment.
2. The quantitative thermal crosstalk data for the 8×8 array is absent. Resonance shift of MRR i vs. heating power applied to MRR j (thermal crosstalk matrix) must be provided to validate scalability claims to 16×16 arrays. Besides, long-term (hours) classification accuracy drift due to thermal accumulation should be characterized.
3. The main text lacks physical explanation of how training enables high-speed operation in a band-limited channel. Readers may mistakenly assume the hardware itself supports distortion-free 128 Gbaud transmission. Supplementary Note 4 shows the training model includes a 38 GHz LPF, but this critical parameter is insufficiently disclosed in the main text Methods. The manuscript does not specify whether pre-emphasis or partial response signaling is used to transmit 128 Gbaud within the 38 GHz channel. The authors should explain how the training algorithm compensates for inter-symbol interference (ISI) caused by band-limitation.
4. The text does not provide performance of "conventional $O(N^2)$ architecture under the same bandwidth constraints and training conditions," making it impossible to isolate the superiority of the "temporal convolution kernel" architecture itself. Comparing $O(N^2)$ vs. $O(N)$ architectures at matched symbol rates (e.g., 16 Gbaud) to isolate the architectural benefit from the speed benefit is welcome.
5. The discretization of continuous-time impulse response h(t) into kernel h[n] (Eq. 5) assumes precise symbol synchronization. At 128 Gbaud, the symbol period is ~7.8 ps. The manuscript lacks analysis of how timing jitter (even at the sub-picosecond level) affects convolution accuracy. Given that photonic systems typically face thermal drift and mechanical vibrations, a quantitative analysis of jitter tolerance (e.g., classification accuracy vs. timing error) is essential.
6. The definition of effective kernel size N (indices where $|h[n]| > 0.1|h[0]|$) requires justification. Why 0.1? The energy truncation at this threshold may introduce non-negligible approximation errors. A sensitivity analysis showing accuracy vs. kernel truncation level would strengthen the methodology.
7. The network anomaly detection results (Fig. 3d) show accuracy increasing from 93.1% at 1 Gbaud to 96.4% at 128 Gbaud, with a subsequent drop at 512 Gbaud. The authors attribute this to larger effective kernel sizes at higher speeds extracting more temporal features. While physically plausible, alternative explanations must be ruled out: Task-specific temporal structure——Network traffic data (USTC-TFC2016) inherently contains burst patterns and temporal correlations. At 1 Gbaud, the effective kernel size (~1-2 symbols) may be insufficient to capture these correlations, effectively reducing the system to a simple scalar weight with limited feature extraction. At 128 Gbaud, the kernel spans more relevant temporal features. However, this suggests the phenomenon may be task-dependent rather than universal. Would the same trend hold for temporally uncorrelated data?

Reviewer #3

(Remarks to the Author)
General Comments:
The manuscript introduces a photonic neural network scheme utilizing Micro-ring Resonators (MRRs) for time-domain convolution. While the 120 Gbaud data rate is technically ambitious, the physical feasibility of this architecture—especially under high-order modulation formats—requires much more rigorous justification. The core concern is the interplay between the MRR’s narrow spectral linewidth and the actual occupied bandwidth of the 120 Gbaud signal. The authors must address whether the "convolution" claimed is indistinguishable from uncontrolled signal degradation and Inter-Symbol Interference (ISI).

Specific Comments:
#1. High-Order Modulation and Actual Signal Bandwidth
The authors report a 120 Gbaud rate, which presumably involves high-order modulation (e.g., PAM4 or QAM) to achieve such throughput.

Critical Requirement: The authors must provide the actual occupied bandwidth of the modulated signal and compare it directly with the MRR’s resonance linewidth. If these two values are comparable, the filtering effect will induce severe phase and amplitude distortion. The authors should clarify how high-order symbols can be recovered with sufficient fidelity when the filter’s edge-roll-off significantly clips the signal’s high-frequency components.

#2. Latency and Efficiency of the Training Phase
A major hidden bottleneck in MRR-based systems is the weight-tuning/training latency.

Operational Concern: To reach the "optimal state" for convolution, precise adjustment of the optical frequency (via thermal or carrier-injection detuning) is required. This is a time-consuming process, particularly when high-order modulation demands extreme precision to maintain a viable Bit Error Rate (BER). The authors should discuss the effective training overhead: if the time required to stabilize the resonance detuning is orders of magnitude longer than the inference time, the touted high-speed advantage is significantly compromised.

#3. Determinism vs. Stochastic Distortion
In this scheme, "convolution" appears to rely on the temporal broadening of symbols. However, fabricated MRRs suffer from inherent process variations and thermal crosstalk. The authors must explain how they ensure this distortion remains deterministic and reproducible. Without a mechanism to compensate for individual device differences, the symbol overlapping becomes unpredictable, making it nearly impossible to transfer a trained model to different hardware instances or even to maintain stability during long-term operation.

#4. Noise Analysis and SINAD in Low-Precision Regimes
The manuscript estimates a 5-bit precision, but lacks a formal SINAD (Signal-to-Noise and Distortion Ratio) analysis. High-

speed operation combined with the sensitivity of high-order modulation makes the system extremely vulnerable to:

Thermal-induced resonance drift during long-term running;

Accumulated noise from optical amplification (ASE) and high-speed electronic backends.
The authors should provide experimental or simulation data on the long-term stability of the SINAD, particularly how the system maintains its claimed accuracy under continuous operational heating.

#5. Practical Benchmarking for Convolutional Advantages
The MNIST task is insufficient to demonstrate the utility of 120 Gbaud time-domain convolution. The community requires evidence that this scheme can handle tasks where temporal correlation is essential, such as high-order nonlinear impairment compensation in fiber-optic links or complex time-series forecasting. The authors need to demonstrate that this hardware can outperform conventional digital or weighting-based photonic processors in tasks that specifically demand high-speed temporal integration.

Version 1:

Reviewer comments:

Reviewer #1

(Remarks to the Author)
All my previous comments are addressed. I have no further comments and would like to recommend this article for publication.

Reviewer #2

(Remarks to the Author)
The authors have addressed most of my concerns. However, I still suggest the authors change the phrase 'beyond 100 GHz clock rate' in the title to be 'beyond 100 Gbaud' to avoid ambiguity since they actually demonstrated a computing speed of 128 Gbaud and using GHz to represent speed is not very standardized. By the way, some words 'Gbaud' were incorrectly written as 'Gbuad'.

Reviewer #3

(Remarks to the Author)
All my concerns have been revised. Accept as is.

**Open Access** This Peer Review File is licensed under a Creative Commons Attribution 4.0 International License, which permits use, sharing, adaptation, distribution and reproduction in any medium or format, as long as you give appropriate credit to the original author(s) and the source, provide a link to the Creative Commons license, and indicate if changes were made.

In cases where reviewers are anonymous, credit should be given to 'Anonymous Referee' and the source.
The images or other third party material in this Peer Review File are included in the article's Creative Commons license, unless indicated otherwise in a credit line to the material. If material is not included in the article's Creative Commons license and your intended use is not permitted by statutory regulation or exceeds the permitted use, you will need to obtain permission directly from the copyright holder.
To view a copy of this license, visit https://creativecommons.org/licenses/by/4.0/

Dear reviewers,

Thank you for taking the time to review our manuscript NCOMMS-26-024440. We deeply appreciate your valuable comments and constructive suggestions. We have carefully revised the manuscript and the supplementary notes accordingly and highlighted the modifications in blue.

Detailed point-by-point responses and revisions are provided below:

# Response to Reviewer #1

## Comment 1:

In the manuscript, the authors introduce a regime to use MRR as a temporal convolutional kernel instead of a scalar weighting element, achieving much faster operating throughput. The idea is straightforward and the implementation is simple without major revisions to the traditional MRR-based photonic matrix-vector-multipliers. Solid experimental results are also well presented to proof the effectiveness of this approach. With that said, I would make any recommendation after those comments in the below are addressed.

## Response 1:

Thank you for your thoughtful review and positive comments. We believe the response and the revision provided below will address all your comments.

## Comment 2:

1) Although strict and detailed mathematically expression of the MRR's temporal response are given, there lack experimental measurements of the actual temporal responses of those MRRs on the PIC. It looks like the kernel depends on both the detuning and the FWHM of the MRR. The detuning can be off over by the temperature drift and the FWHMs can also be off from the designed value by both environmental drift and fabrication error. So I would like to see the actual measured kernels and their comparison with the authors' presented theoretical analysis.

## Response 2:

Thank you for your insightful comments regarding the discrepancy between the theoretical and actual temporal responses of the MRRs. As you pointed out, the temporal kernels are determined by both the resonance frequency detuning and the linewidth (FWHM) of the MRRs, which may deviate from the designed values due to detuning errors and fabrication variations. To address this concern, we experimentally characterized the actual responses of the fabricated MRR array and compared them with the theoretical kernels derived from our model. **The results show that the measured kernels show good agreement with theoretical predictions, with a root-mean-square error (RMSE) of 0.013. We further conducted simulations to show that the accuracy of the proposed photonic neural network (PNN) remains largely unchanged at such and *even larger kernel errors*.** The details are as follows.

Fig. R1.1 presents the theoretical and experimental kernels of both the through and drop ports for an $8 \times 1$ MRR array, where the 8 MRRs are configured with randomly chosen frequency detuning. The theoretical kernels are calculated using the MRR model. The experimental kernels are estimated by fitting the measured MRR transmission spectra with the MRR model and reconstructing the corresponding kernels. The RMSE between the theoretical and experimental values are 0.013.

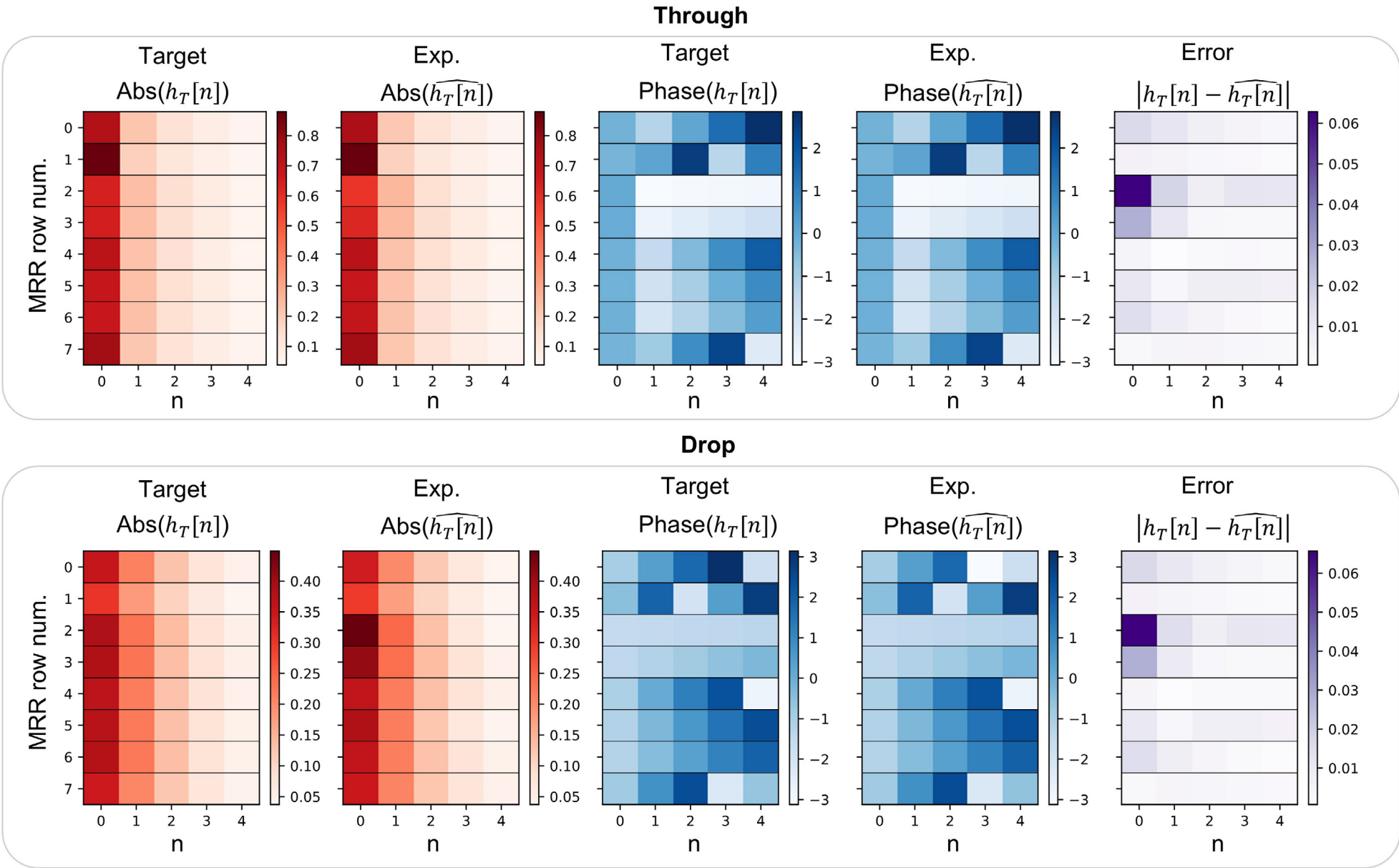


**Fig R1.1** Comparison of theoretical and experimental kernels for an 8 × 1 MRR array.

The small kernel error benefits from the accurate programming of the MRR array. We can program the MRR array precisely and efficiently in the presence of thermal crosstalk and temperature drift using a method called **"chip-in-the-loop" (ChiL)**, which is developed in our previous work [1]. ChiL is an in-situ chip programming method and thus can overcome the discrepancy between the model and device gap. The detailed procedure is provided in Supplementary Note 14. Fig. R1.2a shows the errors of programming the 8×8 MRR array simultaneously. The frequency detuning errors exhibited a standard deviation of 0.138 GHz and a maximum value of 0.430 GHz. To validate that the tuning error would not degrade the accuracy of PNN, we conduct simulations with frequency offsets up to ±1 GHz, which is more than 2× the maximum detuning error observed in our system. As shown in Fig. R1.2b, the accuracy remains largely unchanged across all three tasks demonstrated in the manuscript.

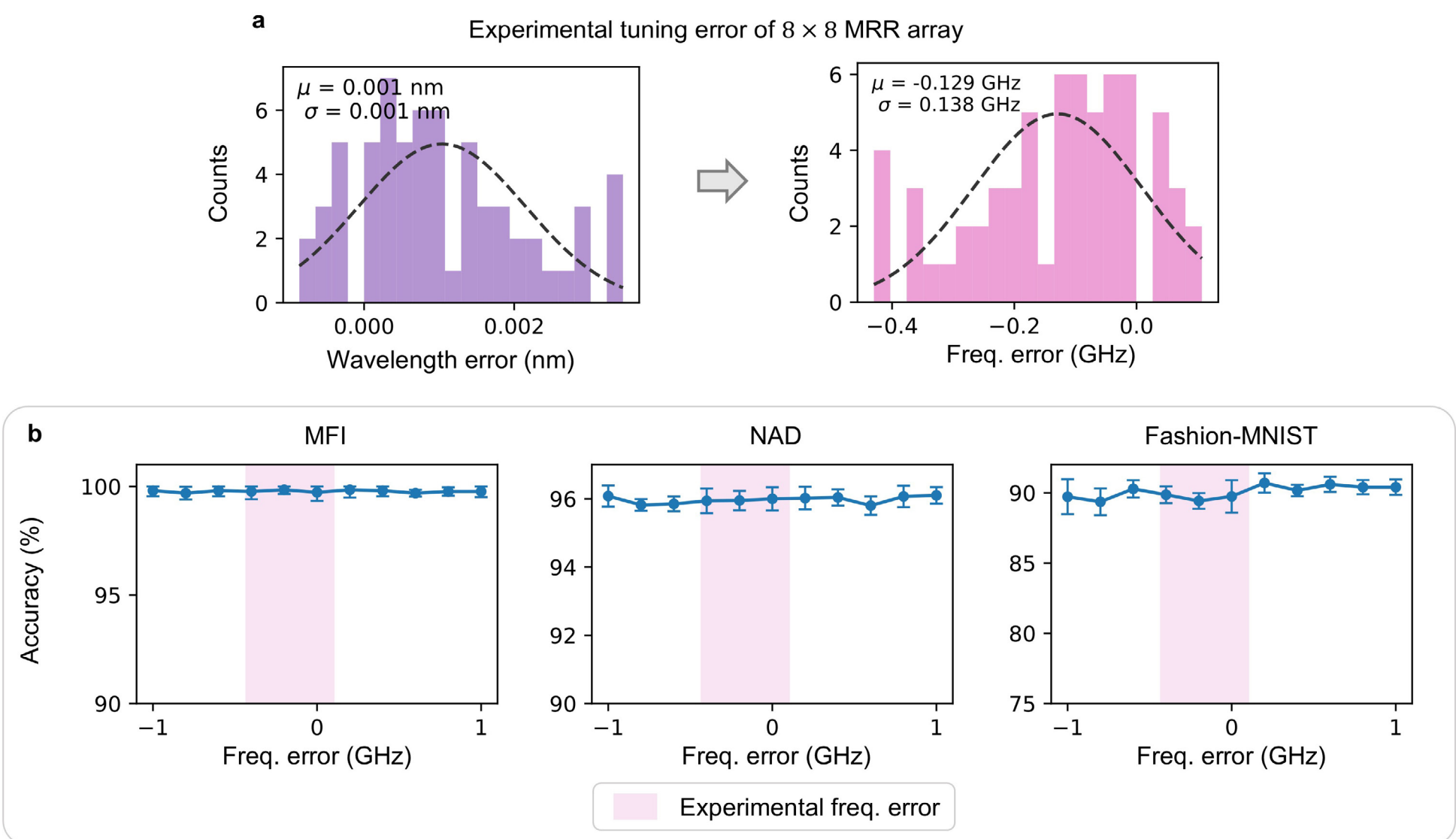


**Fig R1.2 a** Experimental wavelength and frequency tuning error of the 8 × 8 MRR array. **b** Simulation results of classification accuracy of PNNs vs. frequency tuning error.

Besides detuning errors, fabrication-induced FWHM and extinction ratio variations may result

in kernel errors. To validate that the fabrication variations would not degrade the accuracy of PNN, we first extract the statistical distributions of the measured FWHM and extinction ratio of the 8×8 MRR array, as shown in Fig. R1.3a. Based on these, we evaluate the accuracy of the PNNs with standard deviation of fabrication variations up to 2×. As shown in Fig. R1.3b, the accuracy remains largely unchanged across all three tasks demonstrated in the manuscript.

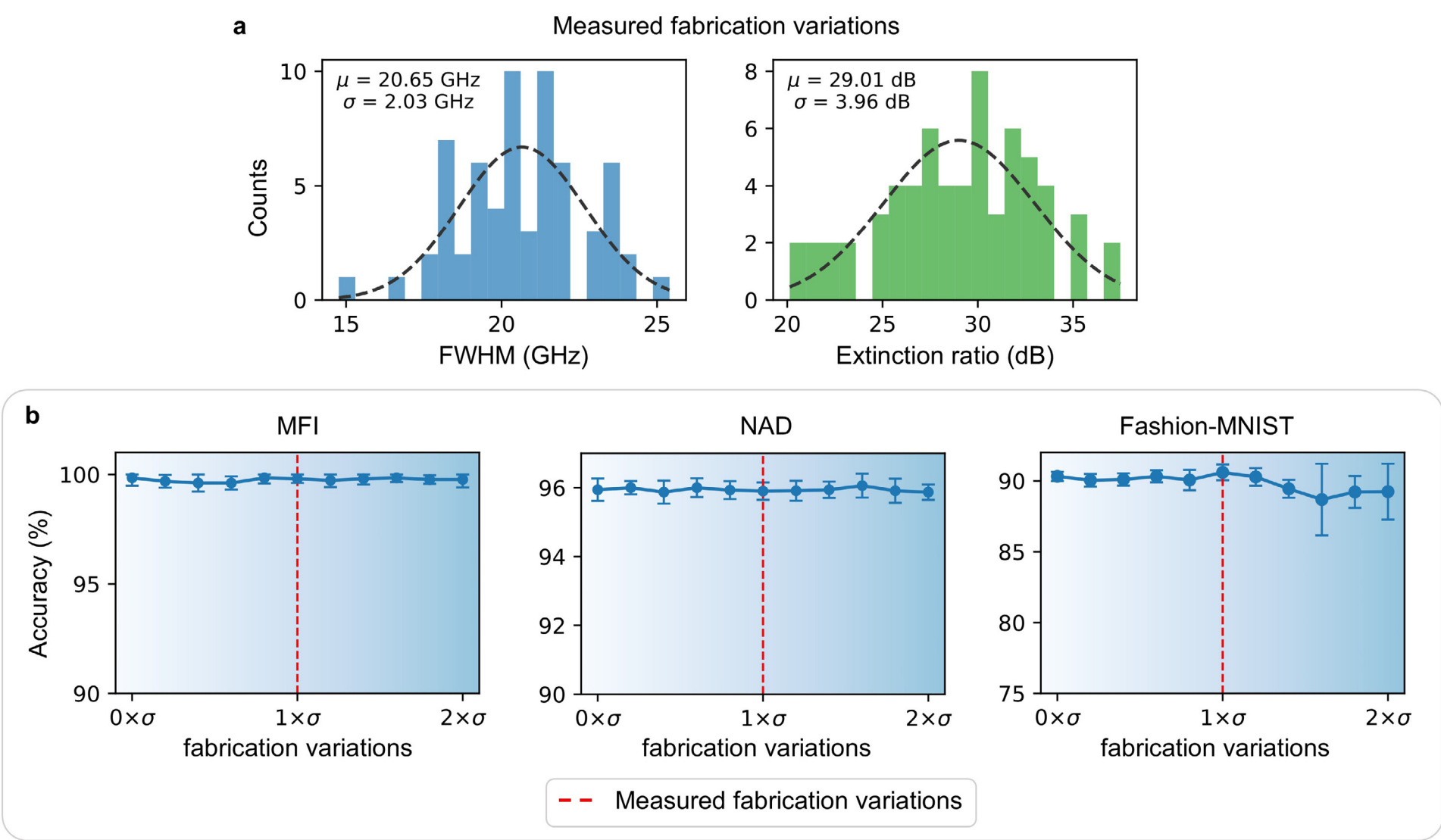


**Fig R1.3** **a** Measured fabrication variations of the 8 × 8 MRR array. **b** Simulation results of classification accuracy of PNNs vs. levels of fabrication variations.

The above experimental and simulation results confirm that the actual kernels of the fabricated MRR array closely match the theoretical analysis, and that the proposed system is robust against kernel errors resulting from resonance detuning errors and fabrication variations. The robustness of PNNs mainly benefits from the sharpness-aware training [2] method we used.

## Revision 2:

1. In section “2.2 Programming and training of the MRR convolution kernel” line 195 to 202, we mention using the chip-in-the-loop method to program the MRR array precisely in the presence of system imperfections:

“Accurate programming of large-scale MRR arrays is challenged by fabrication non-uniformity, thermal crosstalk, and environmental fluctuations. The characterizations of fabrication variations and thermal crosstalk coefficient are provided in Supplementary Note 12 and 13, respectively. To address these challenges, we employ the chip-in-the-loop approach for on-chip configuration. This method enables efficient and accurate programming of large-scale MRR arrays to the desired detuning positions while inherently accounting for fabrication imperfections and thermal crosstalk effects. (see Supplementary Note 14 for details).”

2. In section “3 Discussion” line 449 to 459, we discuss the robustness of the proposed PNN against fabrication variations and detuning errors:

“Photonic integrated circuits face practical challenges arising from fabrication variations and thermal crosstalk effects. Nevertheless, by employing the chip-in-the-loop method, all MRRs can be programmed to their target resonance detuning states with high precision, achieving a residual frequency offset of about 0.1 GHz (see Supplementary Note 14). Discrepancies may still exist between the actual MRR impulse responses and their digital models due to fabrication-induced variations in FWHM bandwidth and residual tuning errors. To address this

problem, we employ sharpness-aware training to improve the robustness of PNNs against these imperfections. For the three tasks demonstrated, the PNNs maintain their accuracy under fabrication variations and detuning errors that are twice as large as those observed experimentally (see Supplementary Note 15)"

3. We provide the details of using chip-in-the-loop method for programming MRR array in Supplementary Note 14.

4. We provide analysis of robustness against detuning errors and fabrication variations in Supplementary Note 15.

**Comment 3:**

> 2) All the three presented tasks utilize not only the convolution layers that implemented on the PIC but also, more or fewer, some FC layers and nonlinear functions that implemented not on the PIC (maybe on a computer). I wonder how much does the PIC contribute to the overall network performance? If possible, the authors can compare the results of the networks with those convolutional layers implemented digitally and that on implemented PIC. In such a way, we can accuracy can the PIC carry out the convolutional layers.

**Response 3:**

Thank you for your valuable suggestions. The MRR-based convolution layers contribute substantially to the network performance. To clarify this point, we compare the proposed PNNs with several digital neural network baselines, as shown in Fig. R1.4.

For each task, we compare four different network architectures:

1. Proposed architecture (**MRR-NN, blue**): convolution operations are implemented by MRR temporal kernels on the PIC, while the fully connected (FC) layers remain digital.
2. FC-only digital network (**FC, orange**): all convolutional layers are removed, leaving only digital FC layers.
3. Parameter-matched digital CNN (**PM-CNN, green**): convolution operations are implemented digitally, with the same number of convolutional parameters matched to the number of MRRs. The convolution operations in this case are conventional ones in which a kernel scans the input feature map and performs local weighted summation to extract spatial features.
4. Larger digital CNN (**L-CNN, red**): convolution operations are implemented digitally with increased model capacity; the convolutional layers contain N-times more parameters, where N is the kernel size. The convolution operations are also conventional ones.

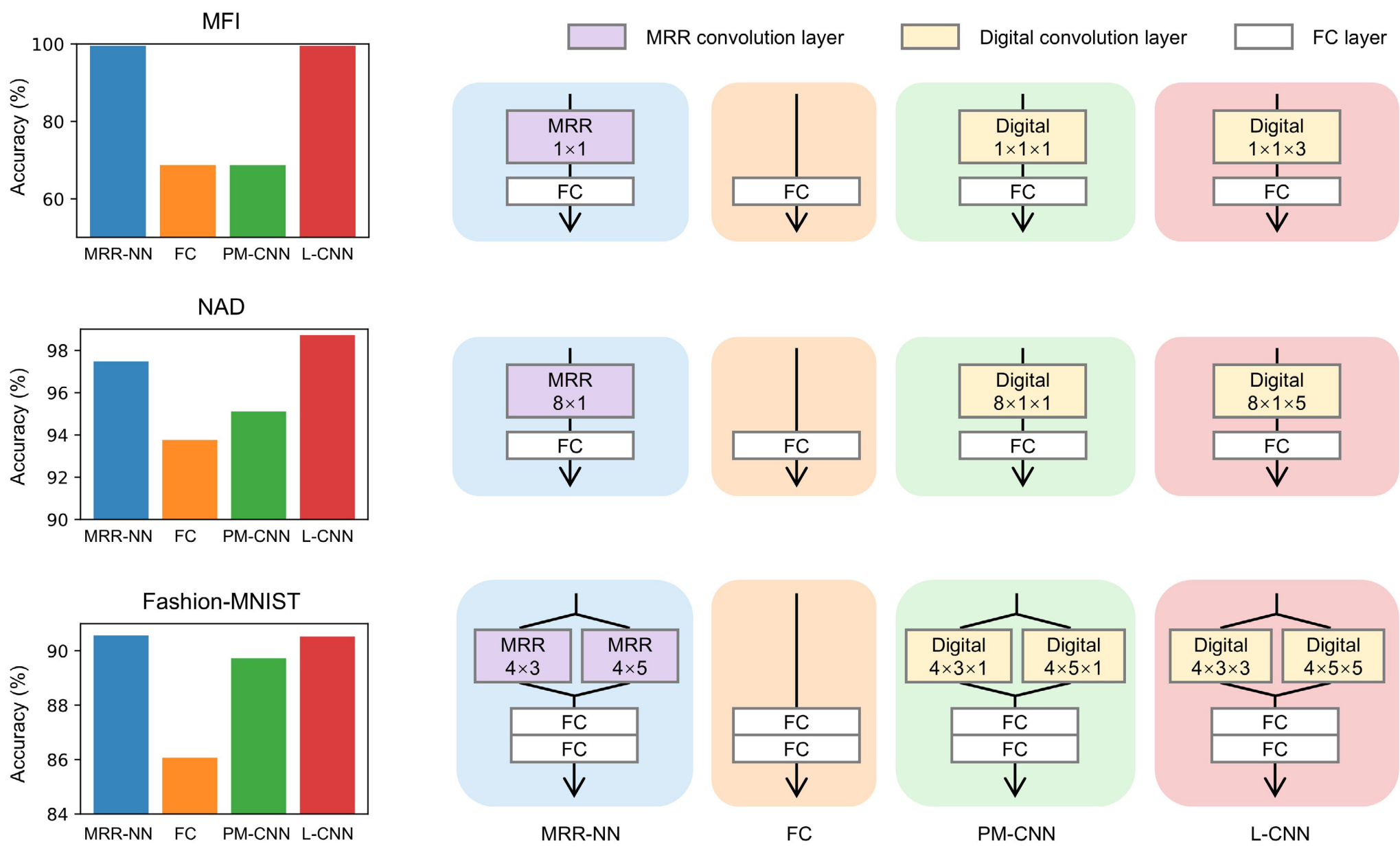


**Fig R1.4** Comparison of the proposed PNNs based on MRR temporal convolution kernels (MRR-NN) and several digital neural networks (FC, PM-CNN, L-CNN).

The comparison results show that the MRR-based convolution layers contribute substantially to the network performance: PNNs with photonic convolution layers (blue) consistently outperform both the FC-only networks (orange) and the digital convolution networks with the same parameter count (green) across all three tasks. Moreover, for the MFI and Fashion-MNIST classification tasks, the PNNs achieve accuracy comparable to that of significantly larger digital convolution networks (red), whose convolution layers contain N-times more parameters, where N is the kernel size. This result indicates that the MRR-based convolution layers implemented on the PIC can perform convolution operations with high parameter efficiency.

These comparisons confirm that the MRR-based temporal convolution kernel performs the core convolution operations effectively and contributes significantly to the neural network performance.

## Revision 3:

1. In section "3 Discussion", line 394 to 403, we discuss the comparison between proposed PNNs and digital neural networks. We provided the details in Supplementary Note 9.

"To evaluate the effectiveness of MRR temporal kernels, we compare the classification accuracy of the proposed PNNs with that of several digital neural networks, as detailed in Supplementary Note 9. The results show that the PNNs with MRR-based convolutional layers consistently outperform both the FC-only network and the parameter-matched digital CNN, in which the number of trainable convolutional parameters is matched to the number of MRRs, across all demonstrated tasks. Moreover, for the MFI and Fashion-MNIST classification tasks, the PNNs achieve accuracy comparable to that of significantly larger digital CNNs, indicating that the MRR-based convolutional layers can perform convolution operations with high parameter efficiency."

## Comment 4:

3) Is it possible to show a task that can be fully implemented on the PIC only without any use of other digital layers?

## Response 4:

Thank you for your insightful comments. The answer is yes. In this work, we mainly focus on the implementation of the MRR-based temporal convolutional layer. Therefore, the fully connected (FC) layers are implemented digitally for simplicity. **However, the FC layers can also be implemented entirely on PICs using existing photonic matrix-vector multiplication architectures**, such as Mach–Zehnder interferometer (MZI) meshes [3], photonic attenuator arrays [4], phase-change-material crossbars [5], and high-speed electro-optic modulator arrays [6]. These approaches have been widely demonstrated for high-speed photonic linear computing. By combining such photonic FC layers with the proposed MRR-based temporal convolution kernels, a fully photonic neural network can be realized without relying on digital FC layers.

**In addition, the proposed MRR temporal convolution can itself directly accomplish certain tasks without any additional digital layers**. To clarify this point, we provide a proof-of-concept fully photonic classification example.

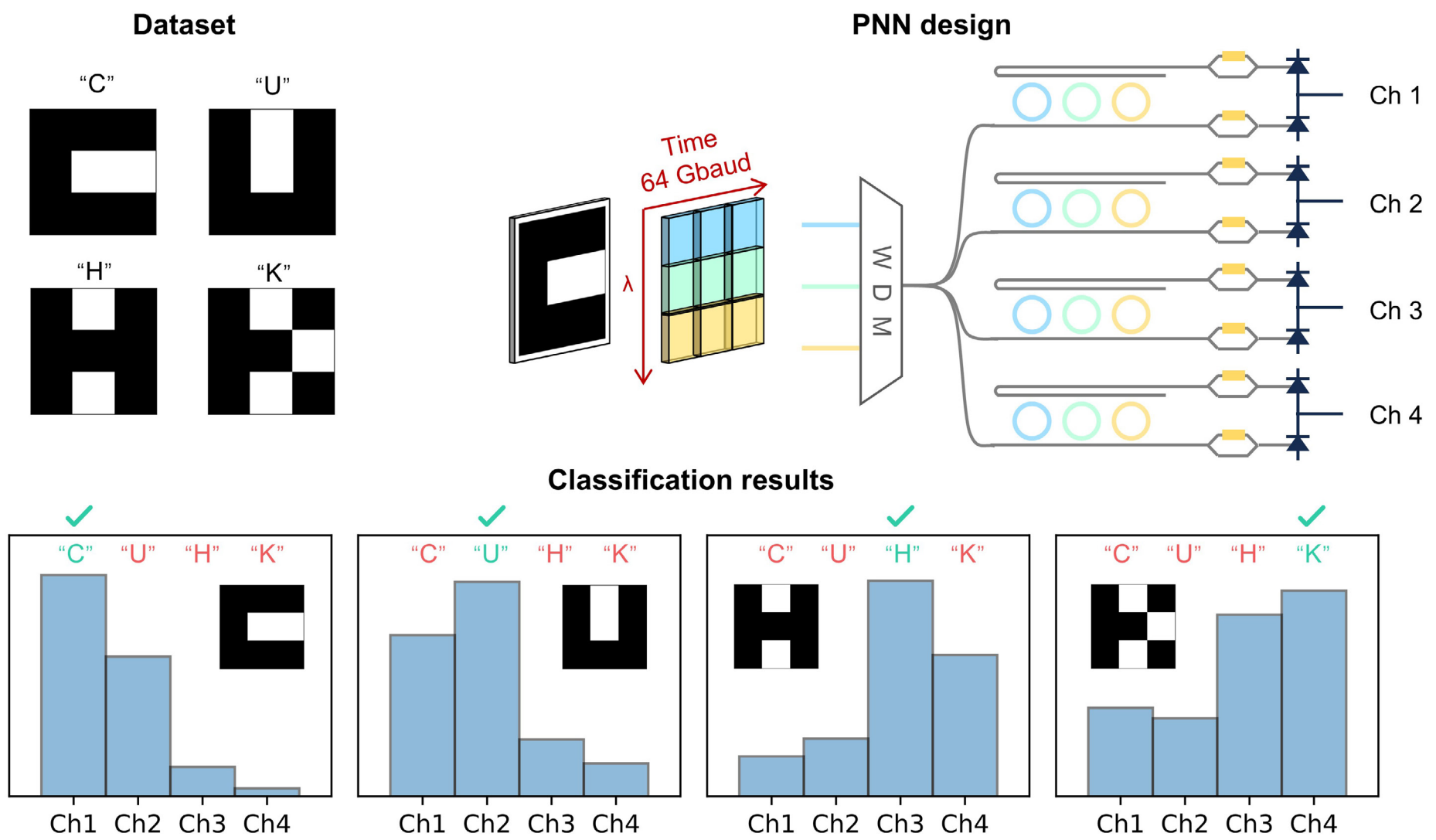


**Fig. R1.5** A MRR temporal kernel array for image classification without any additional digital layers.

As shown in Fig. R1.5, a $4 \times 3$ MRR array is used to classify four $3 \times 3$ binary images corresponding to the letters "C", "U", "H", and "K". In this scheme, the pixel values along the vertical dimension are encoded onto 3 different optical wavelengths, while the horizontal dimension is encoded temporally. The $3 \times 3$ image requires a temporal effective kernel size of 3, therefore the data rate is set as 64 Gbaud according to the impulse response of the MRR on our chip. The MRR array functions as a $4 \times 3 \times 3$ temporal convolution kernel and generates 4 outputs, where each output channel corresponds to one target class.

After training, the classification result can be directly obtained from the photocurrents of the four photodetectors, without requiring any digital FC layers or digital post-processing. The detector corresponding to the correct class produces the highest response for each input image, demonstrating that the MRR-based temporal convolution alone can perform complete classification functionality on-chip.

## Revision 4：

In section "3 Discussion", line 404 to 406, we discuss using only the MRRs for image classification and provide the details in Supplementary Note 19.

"In addition, the MRR temporal kernels themselves can accomplish certain neural network tasks without the FC layers. A proof-of-concept demonstration using only the MRR convolution layer for image classification is provided in Supplementary Note 19."

## Reference:


1. Liu, S. *et al.* Calibration-free and precise programming of large-scale ring resonator circuits. *Optica, OPTICA* **12**, 1113–1121 (2025).
2. Xu, T. *et al.* Physical neural networks using sharpness-aware training. *Nat Commun* **17**, 1766 (2026).
3. Shen, Y. *et al.* Deep learning with coherent nanophotonic circuits. *Nature Photon* **11**, 441–446 (2017).
4. Ashtiani, F., Geers, A. J. & Aflatouni, F. An on-chip photonic deep neural network for image classification. *Nature* **606**, 501–506 (2022).
5. Feldmann, J. *et al.* Parallel convolutional processing using an integrated photonic tensor core. *Nature* **589**, 52–58 (2021).
6. Lin, Z. *et al.* 120 GOPS Photonic tensor core in thin-film lithium niobate for inference and in situ training. *Nat Commun* **15**, 9081 (2024).

# Response to Reviewer #2

## Comment 1:

> This manuscript presents a paradigm shift in photonic computing by redefining microring resonators as programmable temporal convolution kernels rather than scalar weights. The core innovation—exploiting the LTI filter characteristics of MRRs when signal bandwidth exceeds resonance linewidth (~20 GHz) to perform time-domain convolution—is theoretically sound and experimentally validated across three tasks (MFI, NAD, and Fashion-MNIST). The approach elegantly transforms the traditional "bandwidth limitation" into a computational resource.
>
> However, a critical clarification is required regarding the manuscript's central claim of operation "beyond 100 GHz." Supplementary Figure S3c indicates the experimental system has a 3-dB bandwidth of only ~38 GHz, while the MRR linewidth is ~20 GHz. The claimed "128 Gbaud" operation refers to the symbol rate (enabled by training-based compensation of severe inter-symbol interference in the band-limited channel), not analog bandwidth exceeding 100 GHz. This distinction is crucial for scientific accuracy and must be explicitly addressed to avoid misleading the photonics and signal processing communities.
>
> With revisions clarifying the bandwidth constraints, transparent discussion of electronic I/O dominance in energy consumption, and additional characterization of thermal effects, this work represents a significant contribution to photonic AI accelerators suitable for Nature Communications:

## Response 1:

Thank you for your thoughtful comments and valuable suggestions. We appreciate you pointing out the important distinction between analog bandwidth and symbol rate. The reported "exceeding 100 GHz" refers to the symbol rate, rather than an analog bandwidth in this work. To avoid possible misunderstanding and improve scientific clarity, we will revise the title to:

"Microrings as programmable temporal kernels enabling photonic AI beyond 100 GHz clock rate"

The term "clock rate" more accurately reflects the operating speed of the proposed temporal computing architecture and distinguishes it from analog bandwidth. Similar terminology has also been adopted in prior photonic computing papers, such as "High-clockrate free-space optical in-memory computing" [1] and "All-optical computing with beyond 100-GHz clock rates" [2].

## Revision 1:

We change the title of this paper to: "Microrings as programmable temporal kernels enabling photonic AI beyond 100 GHz clock rate".

## Comment 2:

1. When comparing with electronic accelerators or other photonic systems, the system-level energy (including DAC/ADC) must be used, not just the photonic chip consumption. Comparison with other works may be unfair if those works use different DAC/ADC schemes. Please state clearly in the main text that DAC/ADC dominate power consumption and discuss implications for practical deployment.

## Response 2:

Thanks for your comments on the system-level energy consumption. We strongly agree with your comments that DACs and ADCs dominate the system-level power consumption in current photonic AI accelerators.

In this work, the reported system energy efficiency already includes the power consumption of DACs and ADCs. Our approach has higher energy efficiency than others' work because our approach **performs more computing operations per DAC**. The details are as follows.

Considering an $N \times N$ convolution with one output channel, the conventional scheme requires $N^2$ DACs and one ADC, as shown in Fig R2.1a. The total number of computing operations within one DAC conversion time step is $2N^2$ (including $N^2$ real-value multiplications and $N^2$ summations). **Therefore, the number of computing operations per DAC conversion is 2 in the conventional scheme.**

In the proposed scheme, an $N \times N$ convolution requires only $N$ DACs, because one of the convolution dimensions is encoded in the time domain, as shown in Fig R2.1b. Additionally, our scheme performs complex-value convolution at both through and drop ports, resulting in a total of $8N^2$ computing operations within one DAC conversion time step. **Therefore, the number of computing operations per each DAC conversion is 8N in this work.**

Taking $N = 5$, our scheme shows 20× improvement in computing operations per DAC conversion. This leads to a much higher energy efficiency in photonic computing systems where the energy consumption is dominated by DACs and ADCs.

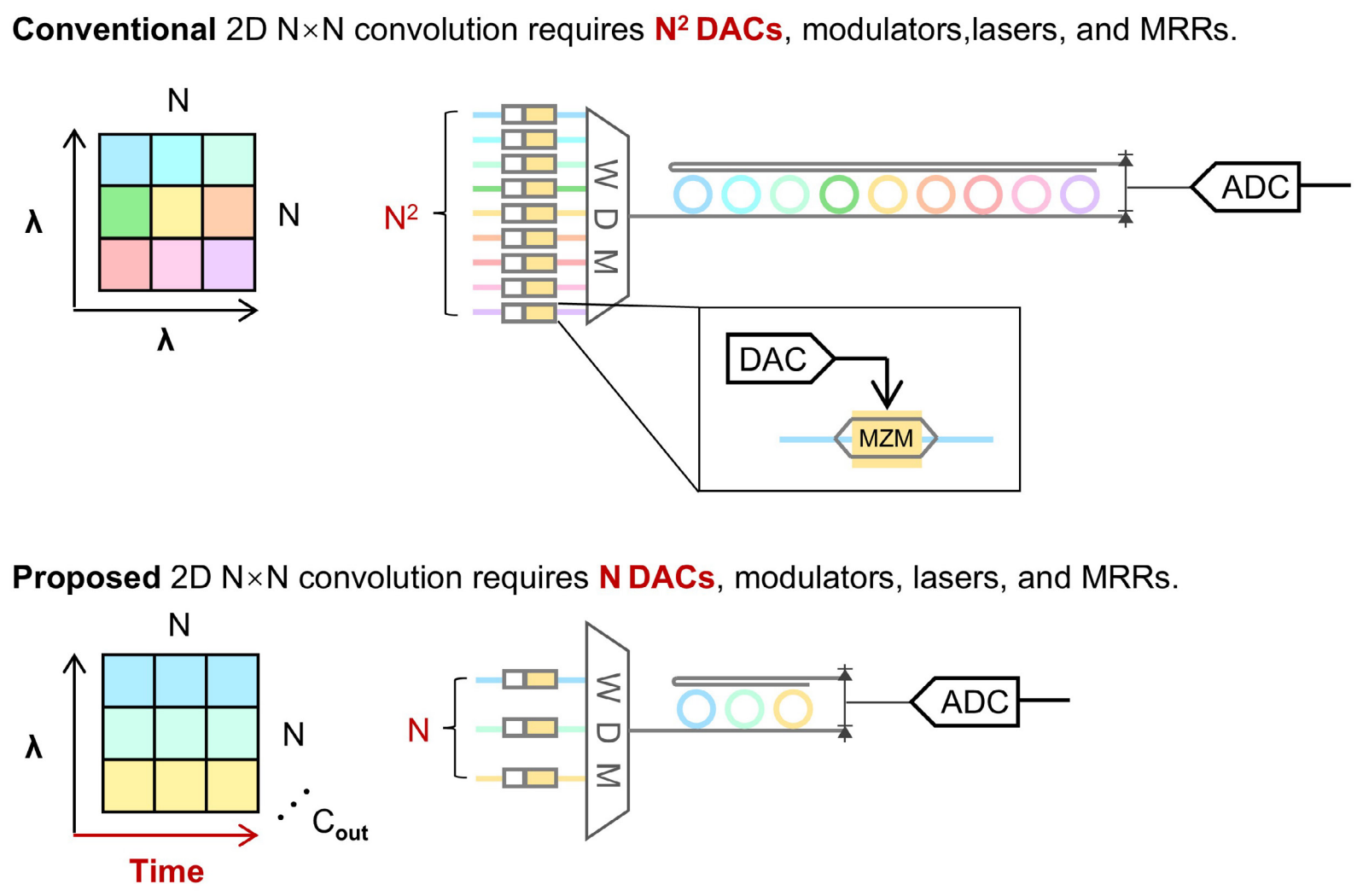


**Fig. R2.1** Comparison of the conventional convolution scheme and the proposed scheme.

For practical deployment, energy efficiency can be further improved by scaling up the MRR arrays. For an $N \times N$ MRR array with $N$ input and $N$ output channels, the computing throughput scales as $O(N^2)$, while the number of DAC and ADC scales as $O(N)$. Therefore,

when DAC/ADC power dominates, the overall energy efficiency scales as $O(N)$. Based on this scaling analysis, a $16 \times 16$ MRR array is estimated to achieve an energy efficiency of 152.8 TOPS/W (see Supplementary Note 8), which is more than 5 times higher than the current experimental implementation.

To ensure a fair comparison with previous works, we now adopt the same estimation methodology adopted in prior photonic computing works[5,6], corresponding to Refs. 16 and 21 in the manuscript, where the DAC/ADC power is calculated as the product of symbol rate and energy consumption per conversion. Then we compare the power consumption and energy efficiency of our work with those of previous works, as shown in Table R2.1. Other works listed in manuscript Table 1 are not listed here, because they either do not calculate power consumption (manuscript Ref 13,15,18, and 19) or only calculate the power consumption of on-chip heaters (manuscript Ref 17 and 20). Table R2.1 shows that the DAC and ADC power consumption in this work is higher than that in previous work due to the higher operating symbol rate. Nevertheless, this work achieves much higher energy efficiency because the proposed temporal convolution architecture substantially increases the computing throughput.

**Table R2.1** comparison of power consumption and energy efficiency

| Ref num. in the main text | Symbol rate (Gbaud) | $P_{\text{DAC}}$ (mW) | $P_{\text{ADC}}$ (mW) | $P_{\text{Total}}$ (mW) | Computing throughput (TOPS) | Energy efficiency (TOPS/W) |
|---|---|---|---|---|---|---|
| 16 [5] | 17 | 46.24 | 34 | 678.74 | 0.136 | 0.2 |
| 21 [6] | 30 | 30 | 84 | 3248 | 0.96 | 0.3 |
| This work | 128 | 235.5 | 281.6 | 4528.0 | 120.832 | 26.7 |

In detail, the power consumption of DAC and ADC is estimated as follows: (1) For the DAC, Ref. [3] reports a 224-Gb/s PAM4 transmitter with an energy efficiency of 0.92 pJ/bit, corresponding to 1.84 pJ per conversion. At the 128-Gbaud symbol rate, the estimated DAC power consumption is therefore: $P_{\text{DAC}} = 128 \times 10^{9} \times 1.84 \times 10^{-12} \times 10^{3} = 235.5$ mW. (2) For the ADC, Ref. [4] reports a 212.5-Gb/s PAM4 receiver with an energy efficiency of 1.1 pJ/bit, corresponding to 2.2 pJ per conversion. Therefore, at 128 Gbaud, the estimated ADC power consumption is: $P_{\text{ADC}} = 128 \times 10^{9} \times 2.2 \times 10^{-12} \times 10^{3} = 281.6$ mW.

According to your suggestion, the revised manuscript now explicitly states the dominance of electronic I/O power consumption and discusses the practical deployment of large-scale photonic AI accelerators.

## Revision 2:

1. We revised Fig. 4b and Fig. 4c to show the reduction of DACs from $O(N^2)$ to $O(N)$ more clearly.

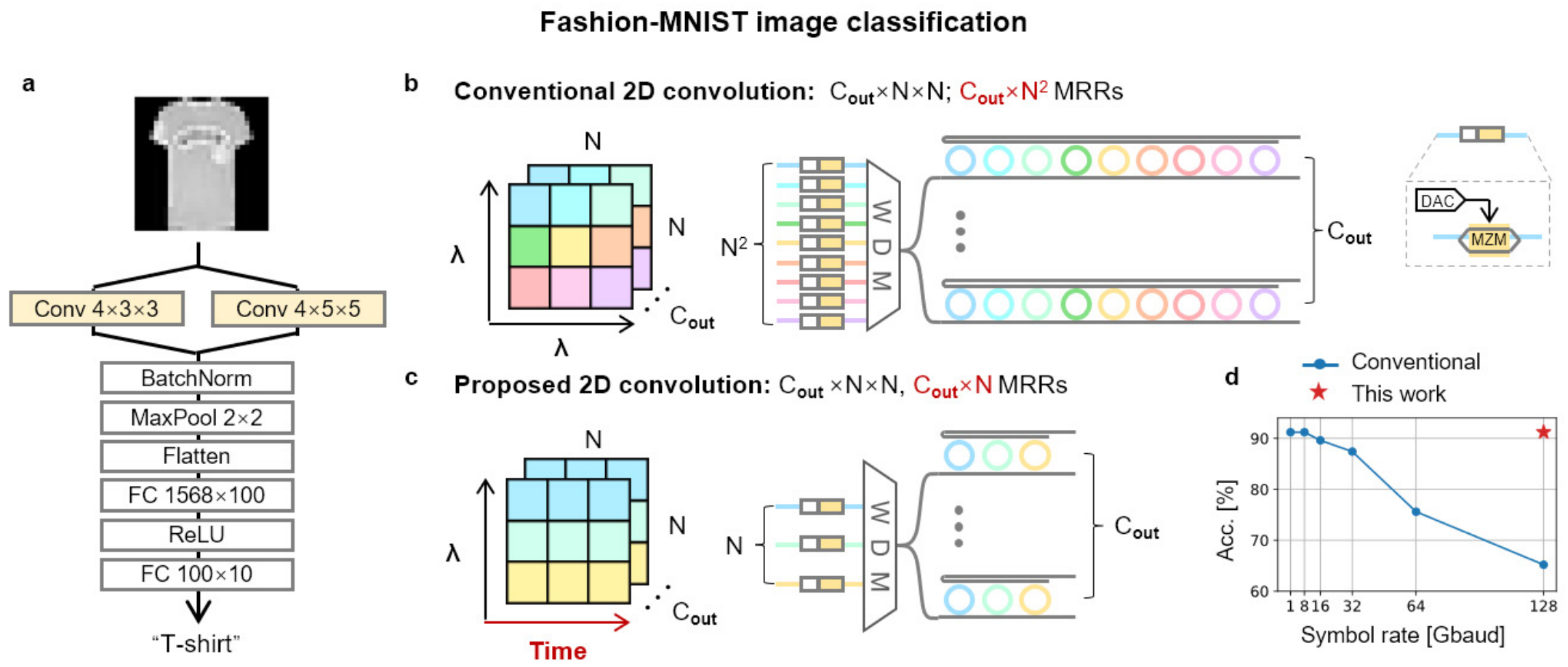


Revised Fig. 4 a-d.

2. In section "3 Discussion" line 422 to 442, we explicitly state the dominance of electronic I/O power consumption. Then we discuss the advantage of the proposed computing architecture in computing operations per DAC conversion. Finally, we discuss the practical deployment of large-scale photonic AI accelerators:

"The power consumption of high-speed optical computing systems is usually dominated by high-speed DACs and ADCs. The proposed convolution architecture achieves higher energy efficiency than conventional schemes because it performs more computing operations per DAC. Considering an $N \times N$ convolution, the conventional scheme requires $N^2$ DACs. The total number of computing operations within one DAC conversion time step is $2N^2$ (including $N^2$ real-value multiplications and $N^2$ summations). Therefore, the number of computing operations per DAC conversion is 2 in the conventional scheme. In the proposed scheme, an $N \times N$ convolution requires only $N$ DACs. Additionally, our scheme performs complex-valued convolution at both through and drop ports, resulting in a total of $8N^2$ computing operations within one DAC conversion time step. Therefore, the number of computing operations per DAC conversion is $8N$ in this work. Taking $N = 5$, our scheme shows $20\times$ improvement in computing operations per DAC conversion.

With the demonstrated computing throughput of 120.832 TOPS in the Fashion-MNIST classification task, the system-level energy efficiency is about 26.7 TOPS/W, where the power consumption of on-chip heaters, lasers, modulators, photodetectors, DACs, and ADCs are all included (see Supplementary Note 8 for details). The energy efficiency can be further improved by scaling up the MRR arrays, because for an $N \times N$ MRR array, the computing throughput scales as $O(N^2)$ while the number of DACs and ADCs scales as only $O(N)$. A $16 \times 16$ MRR array evaluated using the proposed computing paradigm is estimated to achieve an energy efficiency of 152.8 TOPS/W."

3. In Table 1 and Supplementary Note 8, we revise the method for calculating DAC/ADC power consumption and recalculate the energy efficiency.

Table 1: Comparison with representative MRR computing architectures

| Ref | Function of MRR | Data rate (Gbaud) | Per-MRR computing throughput (TOPS) | Total computing throughput (TOPS) | Compute density ($TOPS/mm^2$) | Energy efficiency (TOPS/W) |
|---|---|---|---|---|---|---|
| [15] | Weight | 20 | 0.04 | 0.48 | 0.588 | / |
| [16] | Weight | 17 | 0.034 | 0.136 | 1.04 | 0.2 |
| [17] | Weight | 22.38 | 0.045 | 0.537 | 1.37 | / |
| [18] | Weight | 10 | 0.08 | 1.28 | / | / |
| [13] | Weight | 30.67 | 0.245 | 0.245 | 34.04[a] | / |

| | | | | | | |
|---|---|---|---|---|---|---|
| [19] | Weight | 5 | 0.01 | 0.04 | / | / |
| [20] | Modulation and Weight | 9.83 | 0.0196 | 0.196 | 4.67 | / |
| [21] | Weight | 30 | 0.06 | 0.96 | 0.0245 | 0.3 |
| **This work** | **Convolution** | **128** | **5.12** | **120.832** | **48.05** | **26.7** |
| **This work projected** | **Convolution** | **128** | **5.12** | **1310.72** | **2730.67** | **152.8** |

4. In "Method -> Performance evaluation", line 624 to 632, we explain how to calculate the energy efficiency.

"**Energy efficiency.** Energy efficiency is defined as computing throughput divided by power consumption. The system power consumption is calculated as $P_{\text{total}} = n_{\text{MRR}}P_{\text{MRR}} + n_{\text{laser}}P_{\text{laser}} + n_{\text{IM}}P_{\text{IM}} + n_{\text{PD}}P_{\text{PD}} + n_{\text{DAC}}P_{\text{DAC}} + n_{\text{ADC}}P_{\text{ADC}}$ . In the Fashion-MNIST classification task, the system employs 32 MRRs, 8 lasers, 8 intensity modulators, 8 photodetectors, 8 DACs, and 8 ADCs, leading to total power consumption of 4528 mW. With a computing throughput of 120.832 TOPS, the resulting energy efficiency is approximately 26.7 TOPS/W. We project a 16 × 16 MRR array working at 128 Gbaud can achieve an energy efficiency of 152.8 TOPS/W. The details are provided in Supplementary Note 8."

## Comment 3:

2. The quantitative thermal crosstalk data for the 8×8 array is absent. Resonance shift of MRR i vs. heating power applied to MRR j (thermal crosstalk matrix) must be provided to validate scalability claims to 16×16 arrays. Besides, long-term (hours) classification accuracy drift due to thermal accumulation should be characterized.

## Response 3:

Thank you for your valuable comments regarding thermal crosstalk and long-term thermal stability. We agree that quantitative characterization of thermal effects is important for validating the scalability of large-scale MRR arrays. **To address your concern on thermal crosstalk, (1) we experimentally characterize the thermal crosstalk matrix of the 8 × 8 MRR array and experimentally show that we can program all MRRs precisely to the target resonate state under the thermal crosstalk effect. (2) We conduct further simulations showing that we can program a 16 × 16 MRR array precisely in the presence of stronger thermal crosstalk. (3) To address your concern on long-term classification accuracy, we experimentally measure it and show that the experimental accuracy maintains over 5 hours.** The details are as follows.

The thermal crosstalk data is shown in Fig R2.2. We define the thermal crosstalk coefficient ($c_{i,j}$) between the MRR $[m, n]$ to MRR $[p, q]$ as the ratio between the resonance wavelength shift induced in MRR $[m, n]$ and MRR $[p, q]$ when electrical power is applied only to MRR $[p, q]$, where $i = |m - p|$ and $j = |n - q|$ represent the row and column distances between two MRRs.

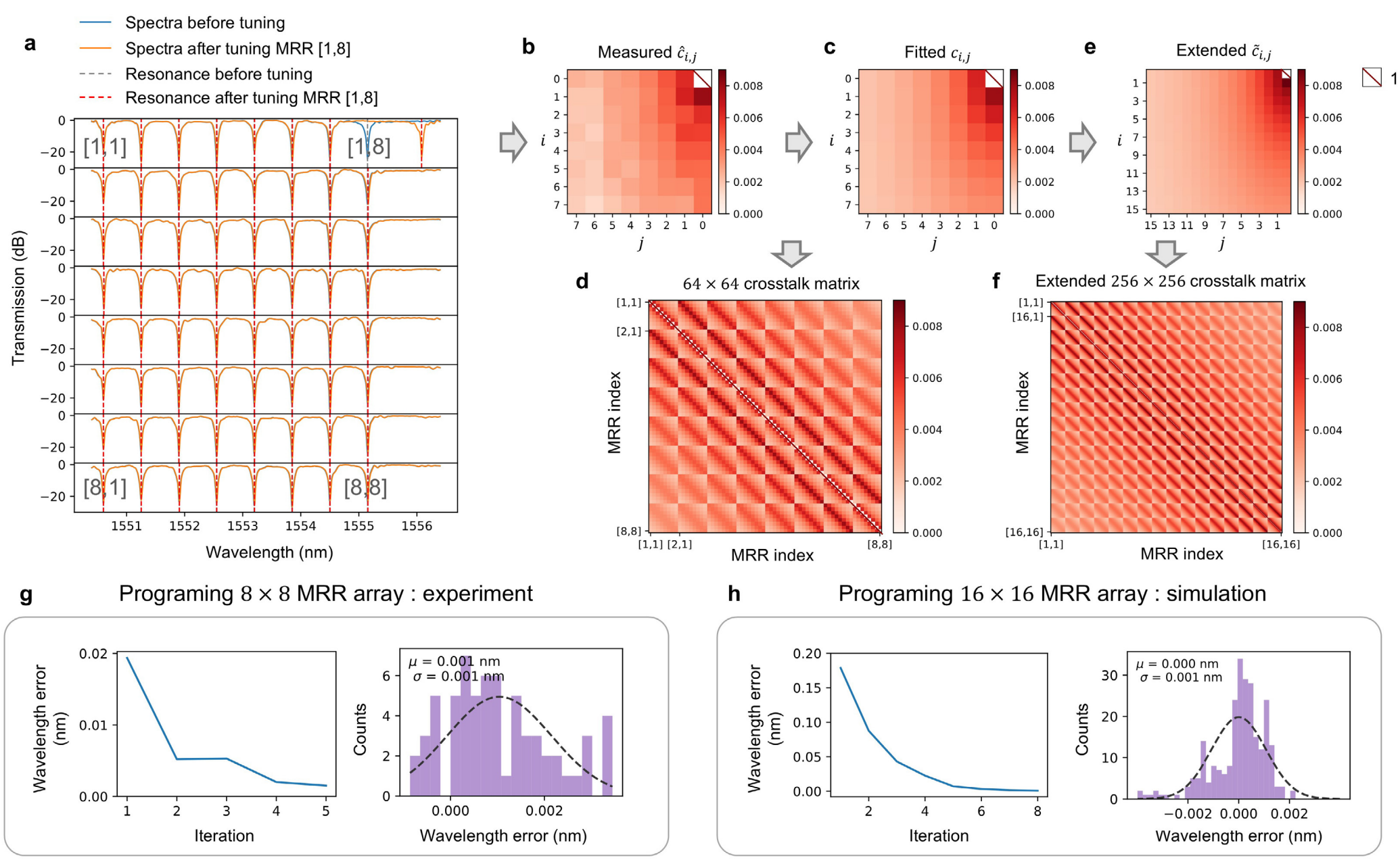


**Fig R.2.2 a.** Spectra of the 8 × 8 MRR array before and after thermally tuning MRR [1,8]. **b**. Experimentally measured thermal crosstalk coefficient $\hat{c}_{i,j}$. **c.** Fitted thermal crosstalk coefficient $c_{i,j}$. **d.** Thermal crosstalk matrix of the 8 × 8 MRR array **e.** Extended thermal crosstalk coefficient $\tilde{c}_{i,j}$ for a 16 × 16 MRR array with the same footprint. **f.** Thermal crosstalk matrix of the 16 × 16 MRR array. **g.** Errors of programing the 8 × 8 MRR array in experiment. **h.** Errors of programing the 16 × 16 MRR array in simulation.

The procedure of characterizing $c_{i,j}$ and thermal crosstalk matrix $M$ is as follows:

1. A picked MRR (MRR [1,8] in our experiment) in the 8 × 8 array is thermally tuned, and the transmission spectra before and after tuning are measured, as shown in Fig. R2.2a. The gray and red dashed lines in Fig. R2.2a indicate the resonance positions before and after thermal tuning, respectively.
2. The experimentally measured $\hat{c}_{i,j}$ is calculated, where $i \in [0,7]$ and $j \in [0,7]$, as shown in Fig. R2.2b.
3. Generally, $c_{i,j}$ decreases as row ($i$) and column ($j$) distance because of the principle of heat conduction. To eliminate the effect of experimental measurement error, a mathematical model is built to fit $\hat{c}_{i,j}$ while ensuring the decreasing property, as described in Eq. R2-1 and shown in Fig R2.2c. The asymmetric coefficients associated with $i$ and $j$ originate from the asymmetric physical spacing and deep-trench structures along the row and column directions of the fabricated chip.

$$c_{i,j} = \begin{cases} \dfrac{1}{14.60\sqrt{3.06i^2 + 15.12j^2} + 99.55}, & (i,j) \neq (0,0) \\ 1, & (i,j) = (0,0) \end{cases} \qquad (\text{R2}-1)$$

4. Based on Eq. R2-1, the 64 × 64 thermal crosstalk matrix of the 8 × 8 MRR array is constructed, as shown in Fig. R2.2d. The element of the matrix is the thermal crosstalk coefficient between any two MRR.

To address possible concerns on the detuning accuracy in such thermal crosstalk effect, we perform experiments on programming the 8 × 8 MRR simultaneously. Here we use a programing method called **chip-in-the-loop [7]**, which enables us to program large-scale MRR array in the presence of thermal crosstalk, developed in our previous work. The experimental results are shown in Fig R2.2g. All are tuned to the target detuning position within 5 iterations, with wavelength error of only about 0.001 nm.

To further evaluate the scalability, we simulate a $16 \times 16$ MRR array, assuming it has the same chip footprint as the current $8 \times 8$ implementation. In this case, the spacing between adjacent MRRs is reduced by half, leading to stronger thermal crosstalk. The corresponding extended thermal crosstalk coefficient $\tilde{c}_{i,j}$ are as described in Eq. R2-2 and shown in Fig R2.2c. The corresponding $256 \times 256$ thermal crosstalk matrix is shown in Fig. R2.2f.

$$\tilde{c}_{i,j} = \begin{cases} \dfrac{1}{14.60\sqrt{0.77i^2 + 3.78j^2} + 99.55}, & (i,j) \neq (0,0) \\ 1, & (i,j) = (0,0) \end{cases} \qquad (R2-2)$$

Using the chip-in-the-loop method, simulations show that the $16 \times 16$ array can still be successfully programmed to the target resonance positions with about 0.001 nm error after 8 optimization iterations, as shown in Fig. R2.2h. These results support the scalability of the proposed architecture to larger MRR arrays.

In addition, to address your concern on long-term (hours) classification accuracy **we continuously run the network anomaly detection (NAD) experiment for 5 hours**. The classification accuracy remains stable at around 95% without observable degradation, as shown in Fig. R2.3. This stability is mainly attributed to 2 factors:

1. During operation, the heat generation and dissipation on the chip reach thermal equilibrium. Continuous heating of the MRRs doesn't lead to significant temperature increasement.

2. We use sharpness-aware-training [8] to train our PNNs, making it robust to environmental fluctuations.

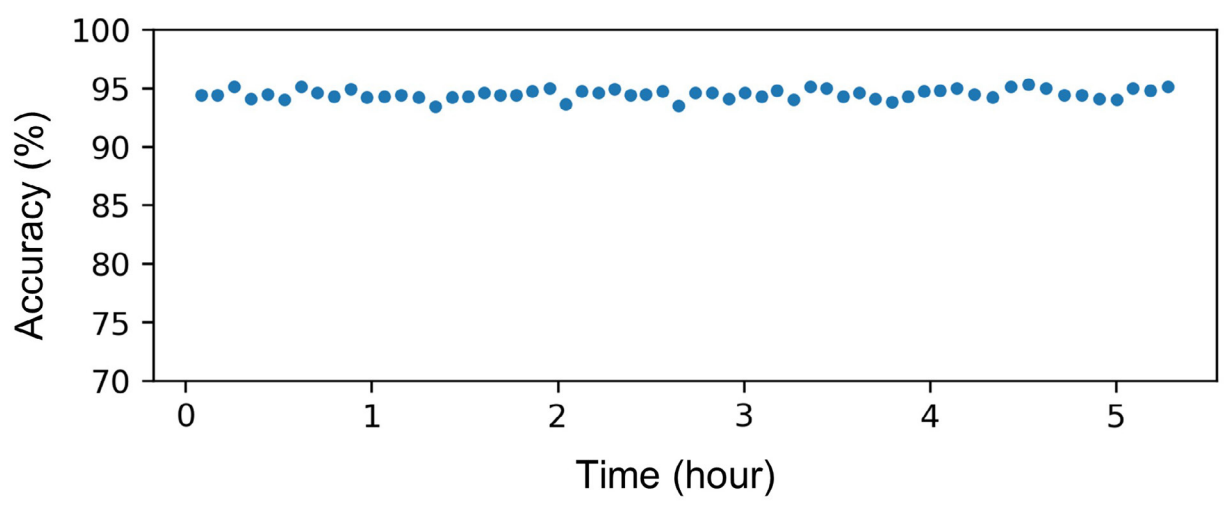


**Fig. R2.3** Long-term accuracy of the NAD task in experiment.

These additional experimental and simulation results have been added to the revised manuscript and supplement to quantitatively clarify the thermal crosstalk behavior, validate the scalability to larger arrays, and demonstrate long-term operational stability.

## Revision 3:

1. In section "2.2 Programming and training of the MRR convolution kernel" line 195 to 202, we mention using the chip-in-the-loop method for MRR configuration in the presence of thermal crosstalk effect:

"Accurate programming of large-scale MRR arrays is challenged by fabrication non-uniformity, thermal crosstalk, and environmental fluctuations. The characterizations of fabrication variations and thermal crosstalk coefficient are provided in Supplementary Note 12 and 13, respectively. To address these challenges, we employ the chip-in-the-loop approach for on-chip configuration. This method enables efficient and accurate programming of large-scale MRR arrays to the desired detuning positions while inherently accounting for fabrication imperfections and thermal crosstalk effects. (see Supplementary Note 14 for details)."

2. In section “3. Discussion” line 462 to 466, we discuss the long-term operation accuracy and provide the experiment results in Fig S18.

“Stability is another important metric in analog computing systems. To evaluate long-term operational accuracy and stability, we continuously performed the NAD task over a period of 5 hours. The classification accuracy remained stable at approximately 95% throughout the experiment, without any noticeable performance degradation (see Fig. S19).”

3. We provide the quantitative thermal crosstalk data of our chip in Supplementary Note 13.

**Comment 4:**

3. The main text lacks physical explanation of how training enables high-speed operation in a band-limited channel. Readers may mistakenly assume the hardware itself supports distortion-free 128 Gbaud transmission. Supplementary Note 4 shows the training model includes a 38 GHz LPF, but this critical parameter is insufficiently disclosed in the main text Methods. The manuscript does not specify whether pre-emphasis or partial response signaling is used to transmit 128 Gbaud within the 38 GHz channel. The authors should explain how the training algorithm compensates for inter-symbol interference (ISI) caused by band-limitation.

**Response 4:**

Thank you for your comments regarding the bandwidth limit of the experimental system. We explain how the training algorithm compensates for inter-symbol interference (ISI) caused by band-limitation in the revised manuscript. The ISI caused by bandwidth limitation is not ignored; it is explicitly incorporated into the forward model and compensated through bandwidth-aware training.

In our system, the experimentally measured electro-optic 3-dB bandwidth is approximately 38 GHz, introducing inter-symbol interference (ISI) for 128 Gbaud signal. We did not use pre-emphasis, equalization, or partial-response signaling to compensate for the ISI during transmission, because they would introduce additional digital signal processing (DSP) overhead during both training and inference. Instead, the bandwidth limitation was treated as an intrinsic response of the analog photonic computing system. During neural network training, a Butterworth low-pass-filter with 3dB bandwidth at 35 GHz is incorporated into the forward and backward propagation model. This approach does not explicitly eliminate ISI. Rather, it considers bandwidth as a part of the PNN and learns to perform the target task in the presence of bandwidth-induced ISI. This training strategy is commonly referred to as bandwidth-aware training [9,10].

To demonstrate the effectiveness of our training method, we perform simulations to compare the performance of the NAD task with and without bandwidth-aware training. As shown in Fig. R2.4, with bandwidth-aware training, the inference accuracy remains above 95% when the system bandwidth varies from 30 GHz to 40 GHz. In contrast, without bandwidth-aware training, the inference accuracy decreases to below 90% when the bandwidth is lower than 40 GHz.

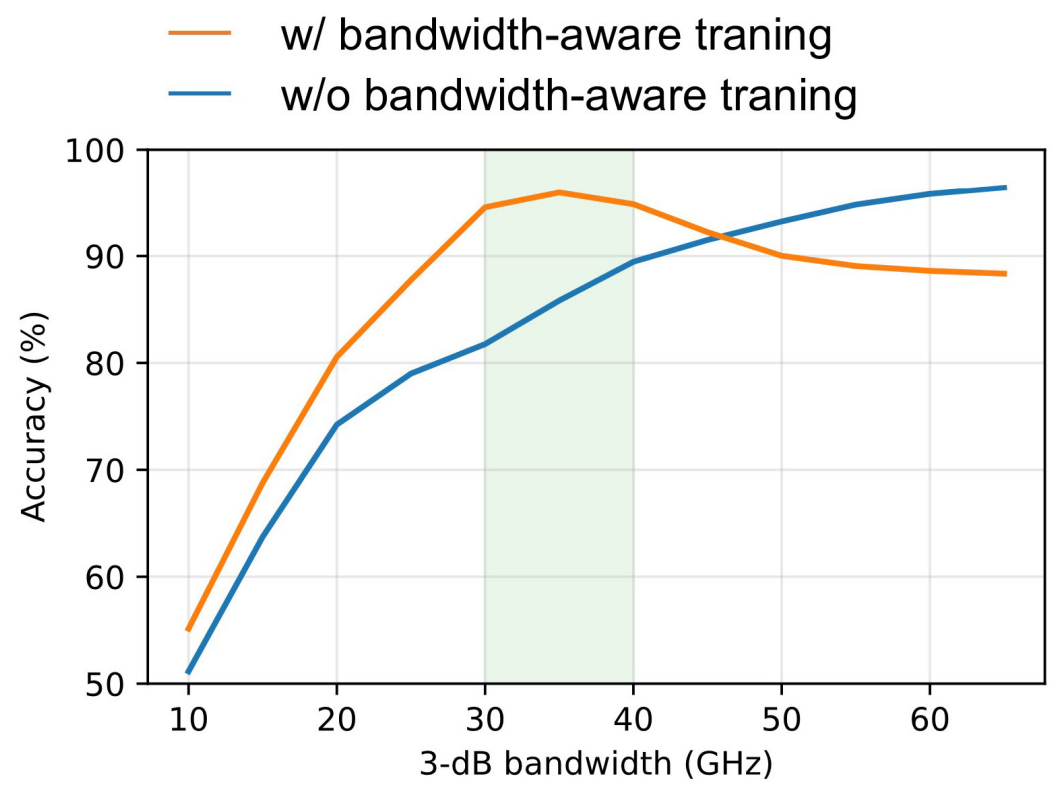


**Fig R2.4** Classification accuracy of NAD with and without bandwidth-aware training

## Revision 4:

1. In section "2.2 Programming and training of the MRR convolution kernel", line 214 to 223, we clarify the bandwidth limit in the experiment system and explain how our training algorithm makes the PNNs work well under the bandwidth limitation:

"In addition, we employ the bandwidth-aware training approach, which incorporates a low-pass filter into the training model to emulate the bandwidth limitation of the physical system. This approach enables PNNs to perform tasks in the presence of bandwidth-induced signal distortions without requiring pre-emphasis, equalization, or other digital signal processing (DSP) techniques. As a result, the proposed system can reliably perform 128 Gbaud convolution using an experimental channel with a bandwidth of only 38 GHz (see Supplementary Note 3). Furthermore, noise-injection and sharpness-aware training are employed to enhance robustness against experimental noise, fabrication imperfections, and residual tuning errors (see Supplementary Note 4 for details).".

2. We provide the results of classification accuracy with and without bandwidth-aware training in Supplement Note 4.

## Comment 5:

4. The text does not provide performance of "conventional O(N²) architecture under the same bandwidth constraints and training conditions," making it impossible to isolate the superiority of the "temporal convolution kernel" architecture itself. Comparing O(N²) vs. O(N) architectures at matched symbol rates (e.g., 16 Gbaud) to isolate the architectural benefit from the speed benefit is welcome.

## Response 5:

Thanks for your comments on the relationship between "architectural benefit" and "speed benefit". In the following response, we first explain why we can reduce the hardware complexity from $O(N^2)$ to $O(N)$. Then we compare the accuracy of these two architectures at matched symbol rates (16 Gbaud and 128 Gbaud) with the same bandwidth constraints and training conditions according to your advice.

In conventional implementations of 2D convolution using MRR arrays, each MRR functions as a scalar weighting element for low-speed signal. Therefore, every element in an $N \times N$

convolution kernel requires an independent wavelength and MRR, resulting in $O(N^2)$ hardware complexity.

In contrast, in the proposed scheme, when the signal symbol rate exceeds the MRR linewidth, each MRR functions as a temporal convolution kernel due to its linear time-invariant (LTI) impulse response. As a result, for 2D convolution, one dimension of the convolution kernel can be mapped into the time domain, while the other dimension mapped onto the wavelength domain. Therefore, an $N \times N$ convolution requires only $N$ MRRs operating at $N$ resonance wavelengths, reducing the hardware complexity to $O(N)$.

Consequently, the reduction of hardware complexity from $O(N^2)$ to $O(N)$ in our architecture **is fundamentally enabled by high-speed temporal processing beyond the MRR linewidth**. In this work, we turn this "speed limit" in conventional $O(N^2)$MRR weighting architecture to "seed benefit" in the proposed $O(N)$ MRR convolution architecture.

According to your suggestions, we compare the $O(N^2)$ and $O(N)$ architectures at the same symbol rates (16 Gbaud and 128 Gbaud) with the same bandwidth constraints and training conditions to further illustrate the principle and advantages of our $O(N)$ architecture. Here we use a neural network consisting of 1 convolutional layer and 1 FC layer for Fashion-MNIST image classification, as shown in Fig. R2.5a. The convolutional layer has the 5×5 kernel size and 4 output channels. In conventional weighting architecture, it requires 25 wavelengths and 100 MRRs, as shown in Fig. R2.5b. In the proposed architecture, by operating at 128 Gbaud and performing time-domain convolution in one of the dimensions, it requires only 5 wavelengths and 25 MRRs, reducing the hardware complexity by 5×, as shown in Fig. R2.5c.

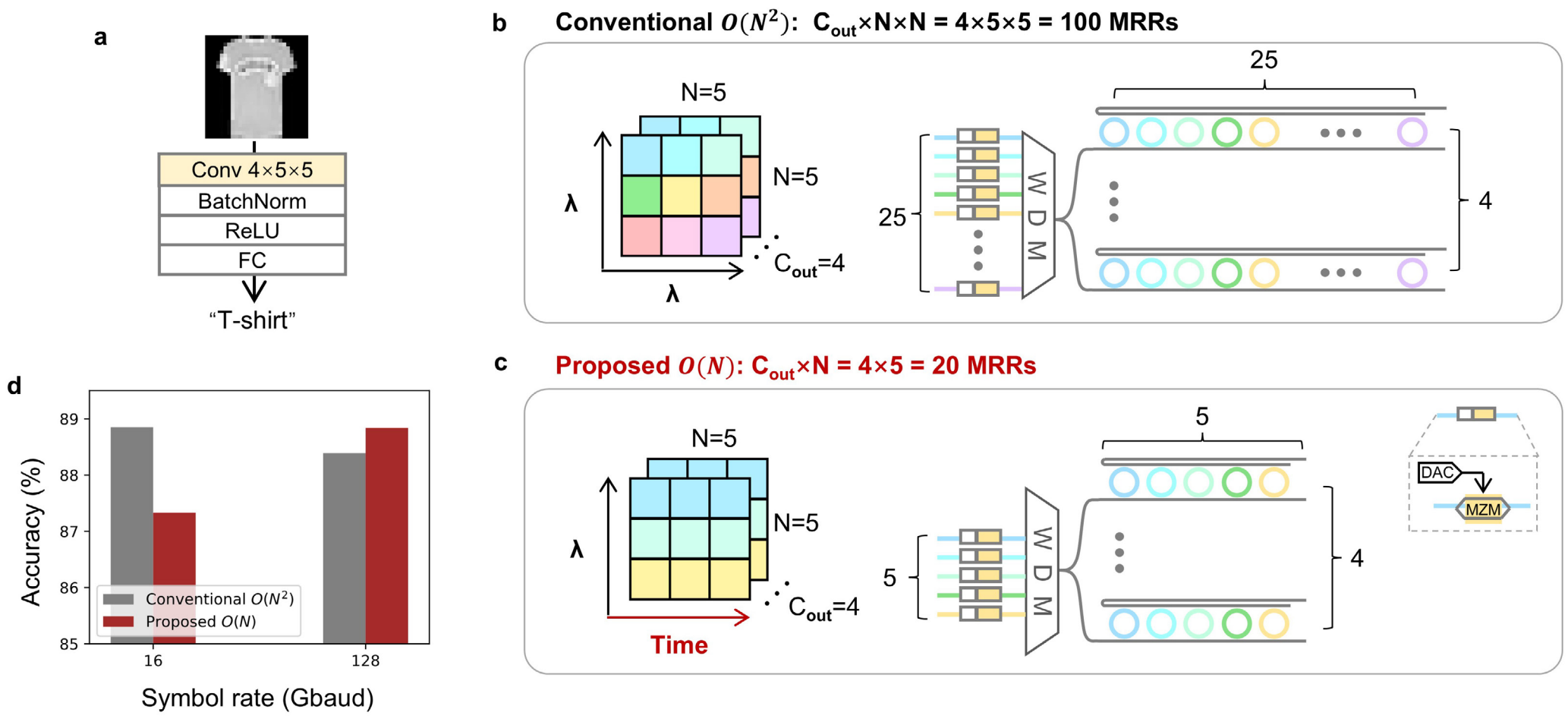


**Fig. R2.5 Comparison of the conventional $O(N^2)$ and the proposed $O(N)$ architecture**

The classification accuracy of both architecture in matched symbol rate is shown in Fig. R2.5d. When operating at 16 Gbaud, MRRs function as scalar weights in both architectures because the symbol rate is smaller than MRR resonance linewidth. Therefore, the $O(N^2)$ architecture performs 2D convolution while the $O(N)$ architecture only performs 1D convolution in wavelength domain. As a result, the accuracy of the $O(N^2)$ architecture (88.85%) is higher than that of the $O(N)$ architecture (87.33%). When operating at 128 Gbaud, for the $O(N^2)$ architecture, though we consider the MRR filter effect during training, the accuracy still slightly decreases from 88.85% to 88.39% because of the computing error caused by unwanted inter-symbol interference. However, for the $O(N)$ architecture, the time-domain convolution effect emerges because the symbol rate exceeds MRR resonance linewidth. Therefore, it can perform 2D convolution in both wavelength and time domain, increasing the accuracy from 87.33% to

88.84%, which is even higher than that of the $O(N^2)$ architecture.

In conclusion, the high symbol rate exceeding MRR linewidth can elevate the performance of the $O(N)$ architecture while degenerating the performance of $O(N^2)$ architecture. One of the core novelties of this work is turning the "speed limit" in conventional architecture into "speed benefit" in the proposed architecture, so that we can get higher computing throughput with less hardware complexity. Therefore, we think it is not necessary to separate the ""temporal convolution kernel architectural benefit" from the "speed benefit," since both arise from the same physical mechanism.

## Comment 6:

5. The discretization of continuous-time impulse response h(t) into kernel h[n] (Eq. 5) assumes precise symbol synchronization. At 128 Gbaud, the symbol period is ~7.8 ps. The manuscript lacks analysis of how timing jitter (even at the sub-picosecond level) affects convolution accuracy. Given that photonic systems typically face thermal drift and mechanical vibrations, a quantitative analysis of jitter tolerance (e.g., classification accuracy vs. timing error) is essential.

## Response 6:

Thanks for your insightful comments regarding the tolerance of timing jitter of the high-speed PNN. To address your concerns, we conduct simulations to test the classification accuracy with different timing errors, concluding that the effect of timing error in the experiments can be fully addressed. The details are as follows.

For the MFI task, we use asynchronous amplitude histograms (AAHs) as classification features. Since AAHs represent the statistical distribution of waveform amplitudes rather than precise temporal samples, they are inherently insensitive to timing errors. As shown in Fig. R2.6b, the classification accuracy remains nearly unchanged over a timing error range of ±1 symbol period for the target 32 Gbaud signal.

For the NAD and Fashion-MNIST tasks, the symbol rate reaches 128 Gbaud. The experimental sampling rate is 256 GSa/s, corresponding to a sampling interval of 3.91 ps. Therefore, the maximum temporal mismatch between an ideal sampling instant and the nearest acquired sample is ±1.95 ps (half a sampling interval), as shown in Fig. R2.6a.

Under such a temporal mismatch, for Fashion-MNIST classification, the accuracy is largely unchanged within twice the maximum timing error, as shown in Fig. R2.6d. This robustness arises because timing error mainly induces small spatial shifts in the images without significantly altering their semantic content. As a result, the trained neural network can still correctly recognize the shifted images.

For the NAD task, the accuracy remains above 94% within the maximum timing error range of ±1.95 ps, and drops below 90% at +3.9 ps. After incorporating timing jitter during training, the PNN becomes significantly more robust against timing error. As shown in Fig. R2.6c, the accuracy remains above 95% within the maximum timing error range and over 94% even at twice the maximum timing error range.

These results demonstrate that the proposed PNN exhibits strong tolerance to timing error.

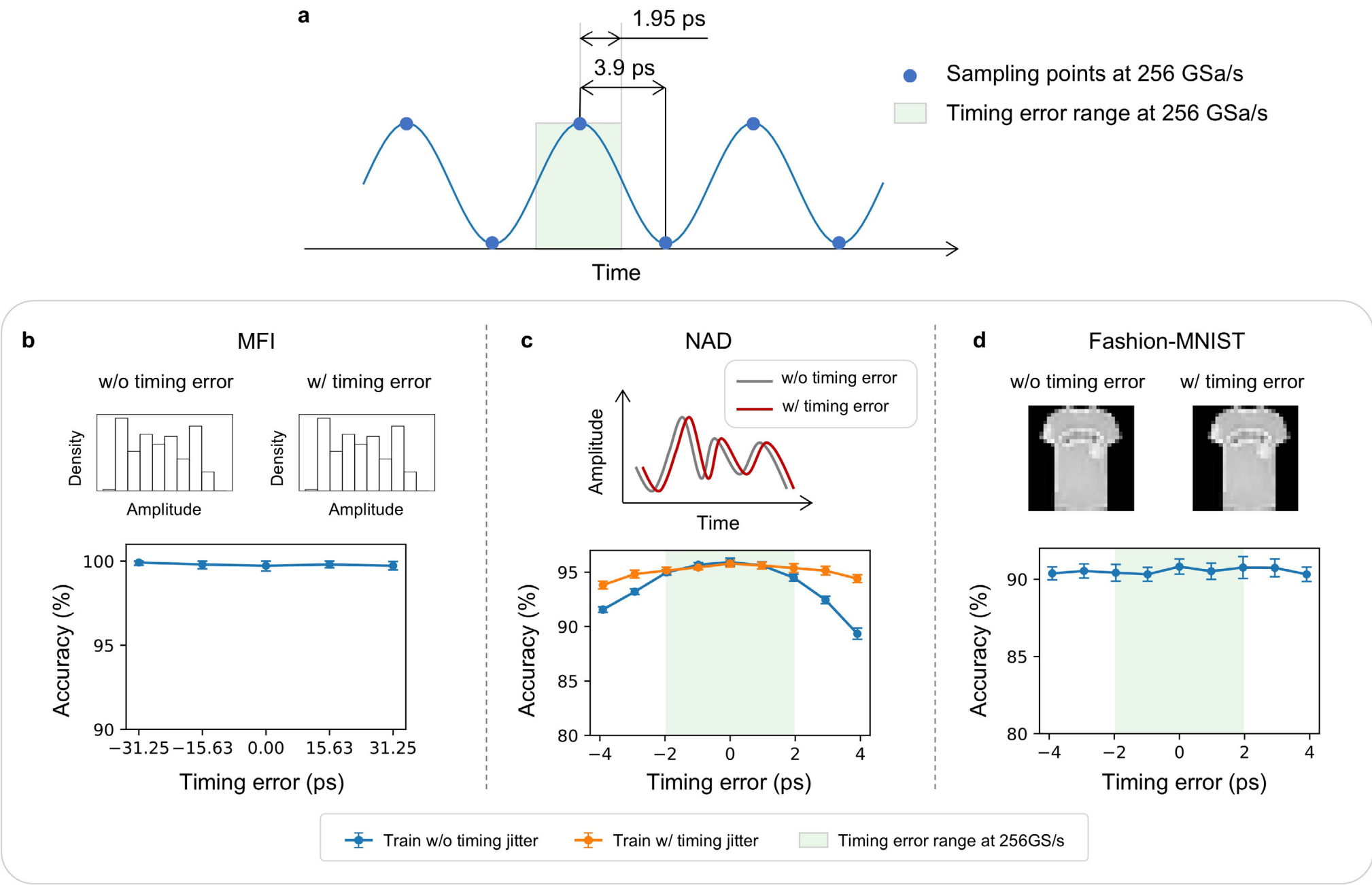


**Fig. R2.6** Classification accuracy with timing error in simulations.

Further, we would like to clarify that the observed accuracy degeneration on NAD task is not caused by discretization of impulse response $h(t)$ into the discrete kernel $h[n]$. The discrete kernel $h[n]$ is introduced solely as a mathematical representation to facilitate comparison with digital neural networks. In the physical implementation, the MRR performs convolution directly in the analog optical domain using the continuous-time impulse response $h(t)$; therefore, no explicit discretization occurs during the optical computation itself. Instead, timing jitter arises at the electrical sampling process for waveform acquisition and digitization. The timing jitter is common to high-speed signal processing systems and is not specific to the proposed MRR-based convolution architecture. Its impact can be effectively mitigated through jitter-aware training as demonstrated above.

In conclusion, timing jitter does not impose a fundamental limitation on the proposed approach and can be adequately addressed by incorporating timing jitter during neural network training.

## Revision 6:

1. In section "3 Discussion" line 459 to 461, we discuss the timing jitter tolerance.

"In addition, by incorporating noise and timing errors during training, the PNNs can work accurately and stably in the presence of noise and timing jitters in the high-speed analog computing system (see Supplementary Note 16 and 17)."

2. We provide the simulation results of timing error tolerance in Supplementary Note 16.

## <u>Comment 7</u>:

6. The definition of effective kernel size N (indices where |h[n]| > 0.1|h[0]|) requires justification. Why 0.1? The energy truncation at this threshold may introduce non-negligible approximation errors. A sensitivity analysis showing accuracy vs. kernel truncation level would strengthen the methodology.

## Response 7:

Thank you for your insightful comments regarding the definition of the effective kernel size. The concept of effective kernel size is to facilitate comparison with digital convolutional neural networks (CNNs), where the convolutional layers usually adopt finite kernel size. In the following, we will explain why we define the effective kernel size $N$ as the largest index satisfying $|h[n]| > 0.1\,|h[0]|$ and provide a sensitivity analysis showing accuracy vs. truncated kernel size. Meanwhile, we would like to clarify that the h[n] is provided solely to facilitate comparison with digital CNNs. In actual neural network training process, we do not explicitly truncate the MRR impulse response. Instead, we model the MRR convolution effect in the frequency domain, so no response truncation is applied.

Fig R2.7a,b show the impulse response $h(t)$ of the MRR and the corresponding discrete convolution kernel $h[n] = \int_{\frac{n}{B}}^{\frac{n+1}{B}} h(t)\,dt$ for the 128 Gbaud signal. The impulse response decays exponentially as time, indicating that, the $h[n]$ contributes less significant to the convolution results as $n$ increases. Therefore, to facilitate comparison with the finite kernel size in digital CNNs, we can define an effective kernel size $N$. In this work, we define $N$ as the largest index satisfying $|h[n]| > 0.1\,|h[0]|$, which corresponds to $N = 5$ for 128 Gbaud signal, as shown in Fig R2.6b. The energy outside this kernel window contributes marginally to the convolution results, which can be validated by testing the accuracy of the PNN using different truncated kernel sizes.

Here we test the accuracy of NAD tasks using different $N$ in time-domain simulations. As shown in Fig R2.7c, the accuracy remains high and nearly unchanged for $N \geq 5$, indicating that a kernel truncated at $N = 5$ accurately captures the convolution behavior of the MRR. In contrast, the accuracy decreases significantly when the $N < 5$, indicating that a kernel truncated at $N < 5$ can't capture the convolution behavior. These results indicate that the kernel coefficients within $0 \leq n \leq 5$ dominate the convolution process, while the coefficient for $n > 5$ contributes only marginally to the convolution result. Therefore, defining an effective kernel size of 5, corresponding to the truncation threshold of 0.1 as shown in Fig R2.6a, is reasonable.

**Notably, the $h[n]$ is solely a mathematical model to facilitate comparison with digital CNNs. <u>In actual neural network training process, we do not explicitly truncate the MRR impulse response</u>**. Instead, we model the MRR convolution effect in the frequency domain, as described in Method and Supplementary Note 4. The input signal is first transformed into the frequency domain using Fourier transform, filtered by the modeled MRR spectral response, and then transformed back into the time domain through inverse Fourier transform. Therefore, the full response of the MRR is naturally included in training without applying hard truncation, which maximally approximates the real-world physical system. The red dashed line in Fig. R2.7b shows the classification accuracy obtained from the frequency-domain simulation. Its performance matches that of the time-domain simulation when $N = 5$, providing additional justification for defining the truncation threshold of 0.1.

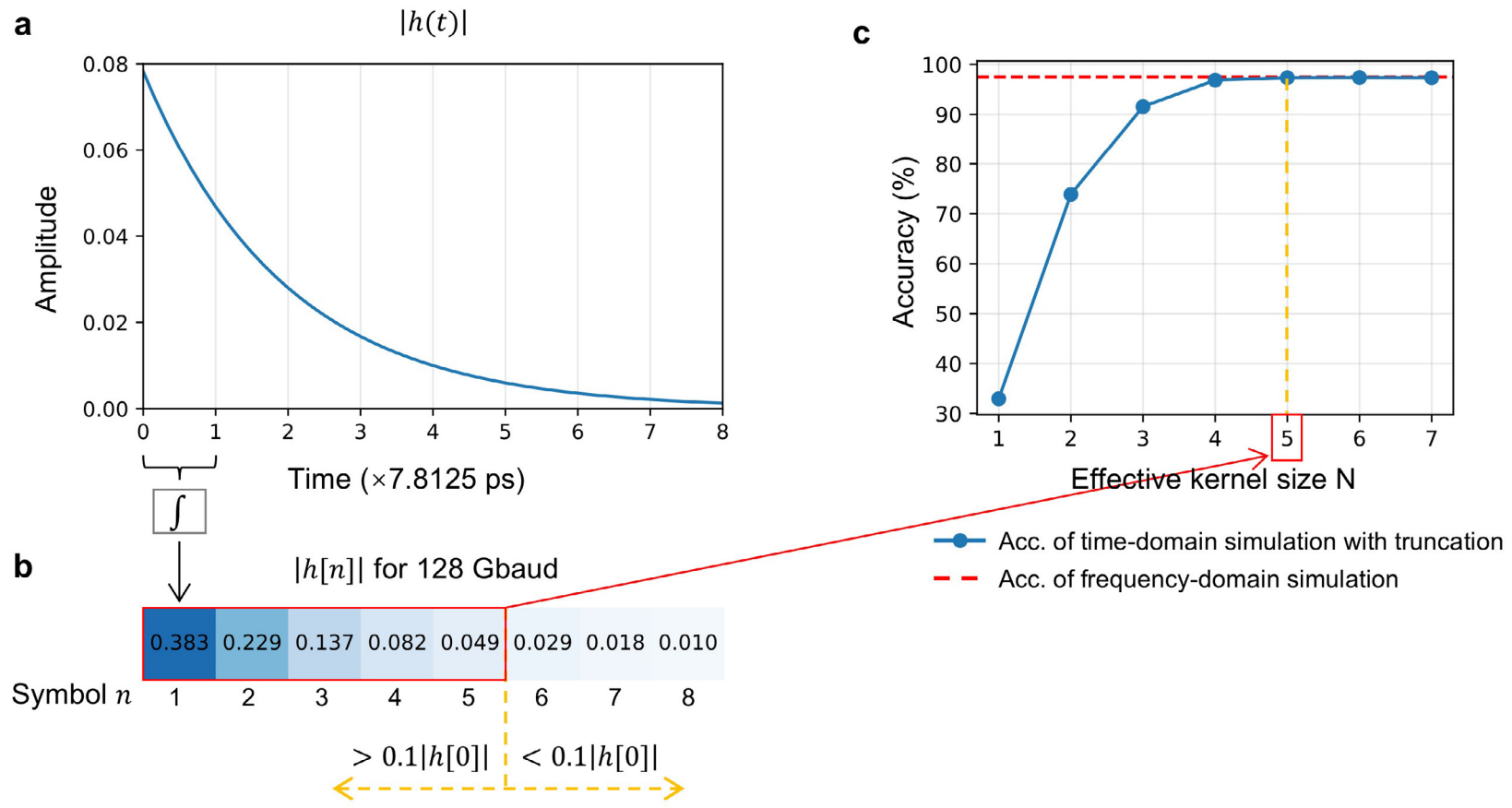


**Fig R2.7** **a** The impulse response $h(t)$ of MRR. **b** The discrete kernel $h[n]$ for 128 Gbaud signal, where truncation threshold of 0.1 corresponds to effective kernel size of 5. **c** Accuracy vs. effective kernel size for the NAD task at 128 Gbaud.

## Revision 7:

1. In section "2.1 Ultrafast convolution based on impulse response of MRR" line 147 to 155, we explain why and how we define the effective kernel size:

"Convolution in digital convolutional neural networks (CNNs) is typically implemented using discrete kernels with finite size. To enable a direct comparison with digital CNNs, the impulse response of an MRR can be represented as an equivalent discrete convolution kernel, $h[n] = \int_{\frac{n}{B}}^{\frac{n+1}{B}} h(t)\,dt$,where $B$ denotes the symbol rate. As the impulse response of a passive MRR $(at_1t_2 < 1)$ decays exponentially in time, we define the effective kernel size $N$ as the largest index satisfying $|h[n]| > 0.1\,|h[0]|$, because contributions from coefficients beyond this range have minimal impact to the convolution result (see Supplementary Note 2). Consequently, the optical convolution by MRR can be approximately described as: $y[n] = \sum_{i=0}^{N} x[n-i]h[i]$."

2. In section "2.2 Programming and training of the MRR convolution kernel", line 206 to 213, we explain that we model the MRR in the frequency domain and clarify that there is not hard truncation of the impulse response during training.

"During training, we model the MRR in the frequency domain according to Eqs. (1) and (2). The input signal is first transformed into the frequency domain through Fourier transform, filtered by the frequency response of the MRR, and then transformed back into the time domain through inverse Fourier transform. This frequency-domain model naturally accounts for the full response of the MRR without imposing an artificial truncation on the kernel length, thereby providing a more accurate approximation of the real-world physical system."

3. We provide the simulation results of accuracy vs. truncated kernel size in Supplement Note 2.

## <u>Comment 8:</u>

7. The network anomaly detection results (Fig. 3d) show accuracy increasing from 93.1% at 1 Gbaud to 96.4% at 128 Gbaud, with a subsequent drop at 512 Gbaud. The authors attribute this to larger effective kernel sizes at higher speeds extracting more temporal features. While physically plausible, alternative explanations must be ruled out: Task-specific temporal structure——Network traffic data (USTC-TFC2016) inherently contains burst patterns and temporal correlations. At 1 Gbaud, the effective kernel size (~1-2 symbols) may be insufficient to capture these correlations, effectively reducing the system to a simple scalar weight with limited feature extraction. At 128 Gbaud, the kernel spans more relevant temporal features. However, this suggests the phenomenon may be task-dependent rather than universal. Would the same trend hold for temporally uncorrelated data?

## Response 8:

Thank you for your insightful comments regarding the generality of the accuracy improvement at higher symbol rates. To our understanding, the phenomenon in the proposed architecture is **general** for both temporal correlated and uncorrelated data. The reason is, increasing the effective kernel size is like enlarging the **receptive field** of the convolution operation, allowing the network to extract broader contextual features [11]. This effect is widely observed in conventional CNNs and is not restricted to temporal sequence tasks. But the required receptive fields (kernel size) for optimal feature extraction can be different from task to task. The details are as follows.

For the NAD task, the network traffic data indeed contains temporal correlations. As the symbol rate increases, the larger effective kernel spans a broader temporal range and can therefore capture richer temporal correlations, leading to improved classification accuracy.

To further demonstrate the generality for temporally uncorrelated data, we additionally perform simulations on MNIST [12] image classification using the same network structure as that used for the NAD task. The setting is shown in Fig. R2.8a. Each image is reshaped into a 1 × 784 one-dimensional sequence, followed by an MRR convolution layer for feature extraction and a fully connected layer for classification. The relationship between classification accuracy and symbol rate is shown in Fig. R2.8b. The accuracy increases from 91.6% to 94.3% as the symbol rate increases from 1 Gbaud to 256 Gbaud, and then slightly decreases to 94.1% at 1024 Gbaud. These results indicate that the benefit of larger effective kernel sizes is not limited to explicitly temporal datasets such as network traffic signals but can also improve feature extraction for data with broader contextual structures.

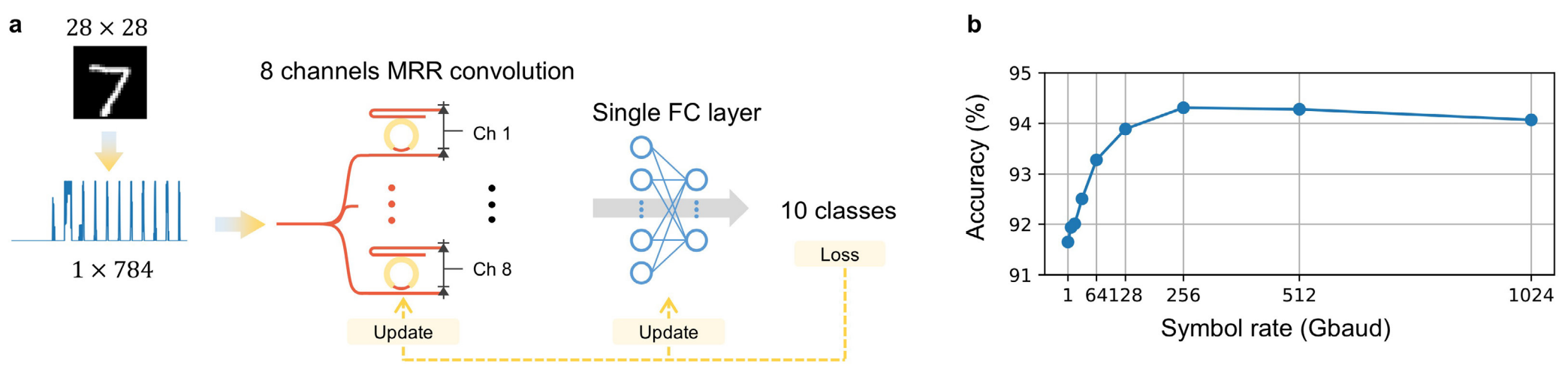


**Fig. R2.8 a** PNNs based on MRR temporal kernels for MNIST classification. **b** Classification accuracy with symbol rate.

We would like to clarify that higher symbol rate doesn't necessarily guarantee higher accuracy. Different tasks may require different receptive fields for optimal feature extraction. For

example, the best symbol rate for the NAD task is 128 Gbaud (see Fig 3d in the manuscript), while the best symbol rate for the MNIST classification task is 256 Gbaud (Fig R2.8b) using the same architecture. Consequently, the optimal effective kernel size, and correspondingly the optimal symbol rate, is task dependent.

**Revision 8:**

1. In section "2.1 Ultrafast convolution based on impulse response of MRR", line 151 to 158, we explain that increasing the symbol rate generally expand the receptive field of CNN, allowing the neural network to extract broader contextual features.

"As the impulse response of a passive MRR ($at_1t_2 < 1$) decays exponentially in time, we define the effective kernel size $N$ as the largest index satisfying $|h[n]| > 0.1\ |h[0]|$, because contributions from coefficients beyond this range have minimal impact to the convolution result (see Supplementary Note 2). Consequently, the optical convolution by MRR can be approximately described as: $y[n] = \sum_{i=0}^{N} x[n-i]h[i]$. Increasing the symbol rate will increase N, which generally expand the receptive filed of the convolutional kernel, allowing the neural network to extract broader contextual features."

2. In section "2.4 Higher speed leads to higher accuracy: ultrafast MRR convolution for network anomaly detection", we revised the manuscript to explain the benefit of the speed advantage more carefully:

Line 269 to 275:

"However, the proposed MRR convolution exhibits a unique advantage: a higher data rate can potentially improve accuracy. This is because higher data rates lead to larger effective kernel size, thereby enlarging the receptive field of the convolution operation and enabling the network to extract broader contextual features. This effect is widely observed in CNNs, although the required receptive fields for optimal feature extraction can vary from task to task."

Line 314 to 317:

"This advantage is not limited to temporal sequence tasks such as NAD. We further show that an MRR-based PNN achieves higher accuracy at higher symbol rates in an MNIST image classification task. The details are provided in Supplementary Note 18."

3. We provide simulation results of MNIST image classification at different symbol rate in Supplementary Note 18.

**Reference:**

1. Liang, Y. *et al.* High-clockrate free-space optical in-memory computing. *Light Sci Appl* **15**, 115 (2026).
2. Li, G. H. Y. et al. All-optical computing with beyond 100-GHz clock rates. Preprint at https://doi.org/10.48550/arXiv.2501.05756 (2025).
3. Cusmai, M. *et al.* A 0.92-pJ/b PAM-4 and 0.61-pJ/b PAM-6 224-Gb/s DAC-Based Transmitter in 3-nm FinFET. *IEEE Journal of Solid-State Circuits* **60**, 23–34 (2025).
4. Mostafa, A. *et al.* 7.2: A 2.2pJ/b 212.5Gb/s PAM-4 Transceiver with >46dB Reach in 5nm FinFET. in *2025 IEEE International Solid-State Circuits Conference (ISSCC)* vol. 68 138–140 (2025).
5. Bai, B. *et al.* Microcomb-based integrated photonic processing unit. *Nat Commun* **14**, 66 (2023).

6. Wu, Y. *et al.* Integrated photonic 3D tensor processing engine. *Light Sci Appl* **15**, 154 (2026).
7. Liu, S. *et al.* Calibration-free and precise programming of large-scale ring resonator circuits. *Optica, OPTICA* **12**, 1113–1121 (2025).
8. Xu, T. *et al.* Physical neural networks using sharpness-aware training. *Nat Commun* **17**, 1766 (2026).
9. Moralis-Pegios, M. *et al.* Neuromorphic Silicon Photonics and Hardware-Aware Deep Learning for High-Speed Inference. *J. Lightwave Technol., JLT* **40**, 3243–3254 (2022).
10. Moralis-Pegios, M. *et al.* Optics-informed Neural Networks: Bridging Deep Learning with Photonic Accelerators. in *2024 Optical Fiber Communications Conference and Exhibition (OFC)* 1–3 (2024).
11. Krizhevsky, A., Sutskever, I. & Hinton, G. E. ImageNet Classification with Deep Convolutional Neural Networks. in Advances in Neural Information Processing Systems vol. 25 (Curran Associates, Inc., 2012).
12. Lecun, Y., Bottou, L., Bengio, Y. & Haffner, P. Gradient-based learning applied to document recognition. *Proceedings of the IEEE* **86**, 2278–2324 (1998).

# Response to Reviewer #3

## Comment 1:

> General Comments:
>
> The manuscript introduces a photonic neural network scheme utilizing Micro-ring Resonators (MRRs) for time-domain convolution. While the 120 Gbaud data rate is technically ambitious, the physical feasibility of this architecture—especially under high-order modulation formats—requires much more rigorous justification. The core concern is the interplay between the MRR's narrow spectral linewidth and the actual occupied bandwidth of the 120 Gbaud signal. The authors must address whether the "convolution" claimed is indistinguishable from uncontrolled signal degradation and Inter-Symbol Interference (ISI).

### Response 1:

Thank you for your comments regarding the relationship between the proposed MRR temporal convolution and inter-symbol interference (ISI).

After carefully reviewing the comments, we realize that it is important to clarify that **the objective of our system is not high-speed signal communication or data transmission, but high-speed neural-network computation. The influence of the bandwidth-limited response is fundamentally different in an optical communication system and a neural-network-based computing system, as illustrated in Fig. R3.1.**

In **optical communication systems**, the goal is to transmit each symbol with minimal distortion. Accordingly, the performance is typically evaluated by measuring the bit error rate (BER) between the transmitted and received signals. In this context, inter-symbol interference caused by insufficient channel bandwidth is regarded as an unwanted impairment that must be compensated or eliminated.

**In contrast, in a neural network**, the objective is not to recover the exact input symbols, but to extract task-relevant features and generate inference outputs, such as classification labels. The performance is therefore evaluated using task-level metrics, such as classification accuracy, rather than symbol-level fidelity. In this context, interactions between neighboring symbols are not necessarily detrimental. Instead, they can serve as useful computational operations. For example, convolution is a fundamental neural-network operation that intentionally introduces local interactions between adjacent data points and is widely used in both digital [1] and optical [2] neural networks. Therefore, the local symbol interactions introduced by the bandwidth-limited response can be exploited as part of the computation rather than treated solely as transmission impairments.

Fig R3.1 illustrates the difference between the proposed PNNs and optical communication system. In optical communication system (Fig R3.1a), the target is to recover the transmitted data without error, thus any bandwidth limitation can cause ISI and needs to be avoided. However, in PNN (Fig R3.1b), taking the network anomaly detection (NAD) task as example, the target is to classify the category of the network traffic data, and the final output is only 20 symbols, the maximum of which represents the classification results. Therefore, the bandwidth-limited channel introduces local interactions among neighboring symbols, which play a role analogous to convolution operations in neural networks. These interactions allow the system to capture temporal correlations between symbols and are therefore beneficial and essential for

the task.

To be more specific, in the proposed architecture, when the signal symbol rate exceeds the MRR resonance linewidth, the MRR behaves as a linear time-invariant (LTI) filter whose output is the convolution between the input signal and the MRR impulse response. Therefore, the MRR acts as a temporal convolution kernel for neural network computation. Importantly, this response is deterministic, programmable, and incorporated into the neural network training. The resonance frequency of each MRR can be tuned to modify the corresponding temporal convolution kernel, enabling trainable photonic convolution operations. This behavior is fundamentally different from uncontrolled signal degradation in conventional communication systems.

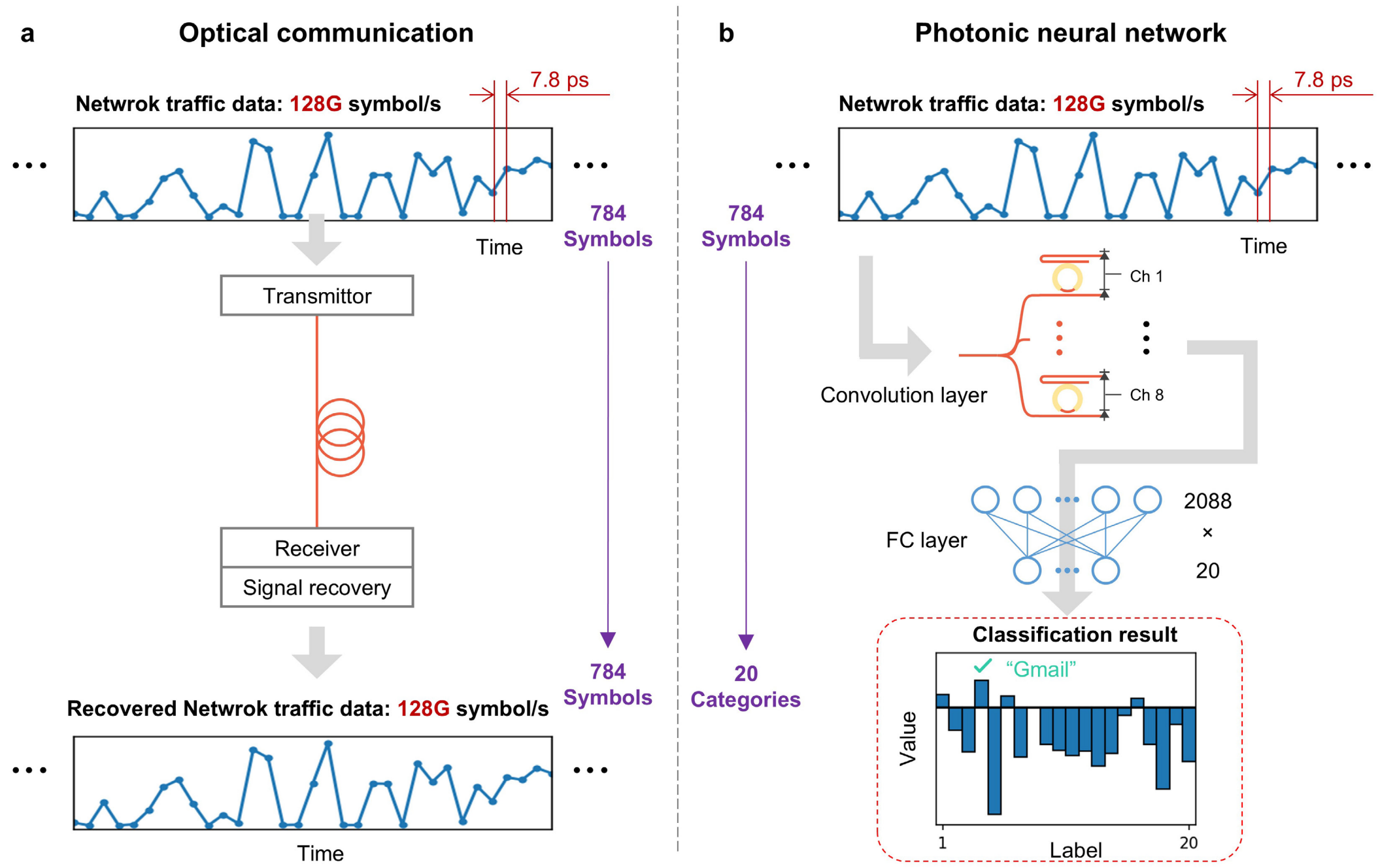


**Fig R3.1 Difference between optical communication and photonic neural network**

The effectiveness of the MRR temporal convolution kernel is experimentally demonstrated through three AI tasks: modulation format identification (MFI), network anomaly detection (NAD), and Fashion-MNIST classification. In all cases, the PNN with the proposed MRR temporal convolution achieves higher accuracy than corresponding networks without the MRR convolution layer, demonstrating that the bandwidth-limited temporal response contributes constructively to computation rather than degrading the signal.

Additionally, we would like to clarify that we do not claim distortion-free or lossless transmission of high-order modulation signals at 128 Gbaud. **As discussed above, the objective of our system is not high-speed communication, but high-speed photonic neural-network computation**. Our demonstration shows that PNN computation can be performed with **input signals at symbol rates up to 128 Gbaud**, where the programmable temporal response of bandwidth-limited MRRs is exploited to generate useful inter-symbol interactions for computation.

The input and output signals of the three demonstrated tasks are further clarified in Fig R3.2.

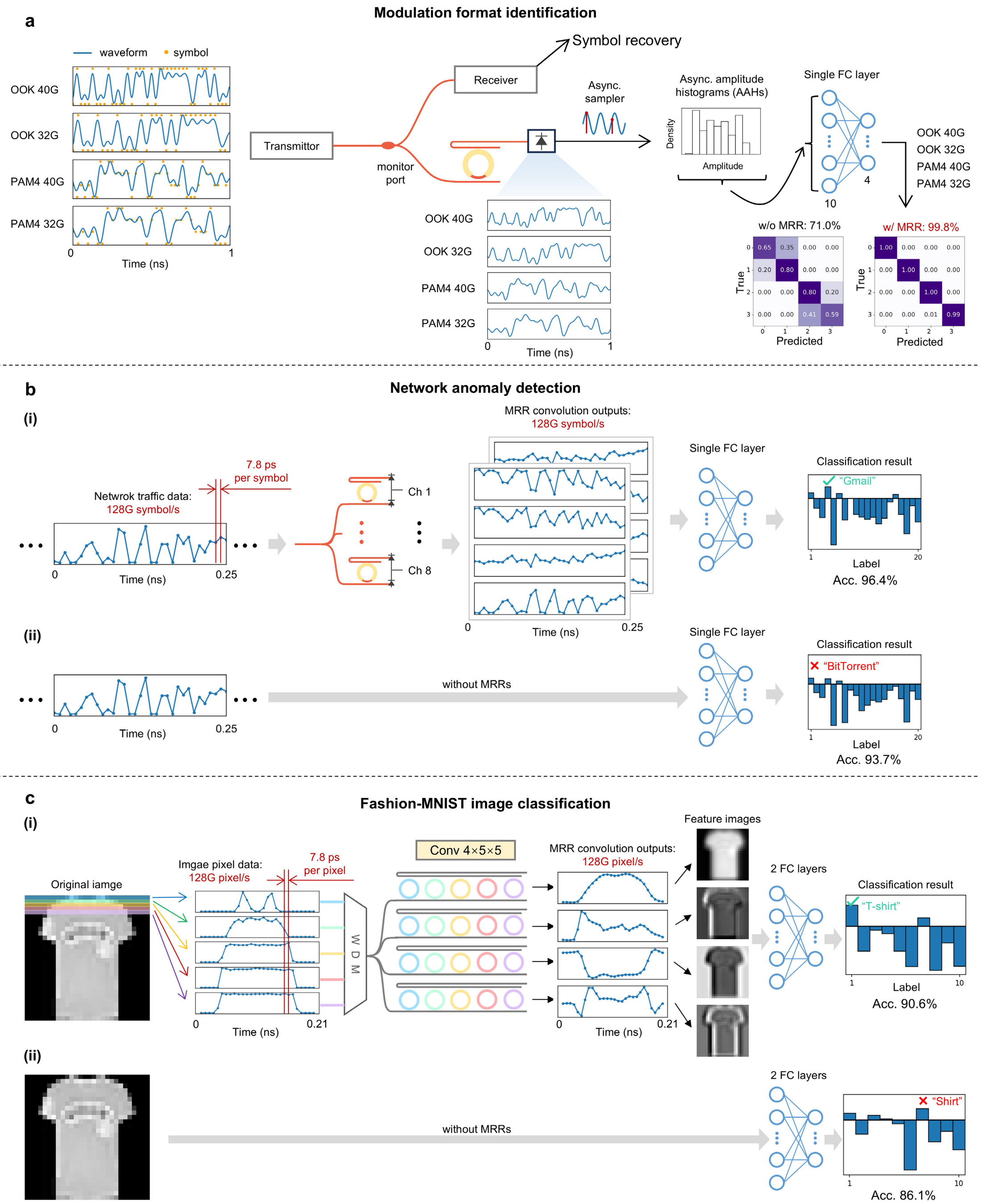


**Fig. R3.2 Data flow in PNNs.**

Fig R3.2a shows the concept of MFI in the optical network. The inputs are 32 Gbaud/40 Gbaud OOK/PAM4 signal. We use an MRR at the monitor port of the optical network for feature extraction. Though the signals after MRR look "distorted", they can be used for classifying the modulation format, achieving a classification accuracy of 99.8%. In contrast, without the MRR, though signals are "undistorted", the classification accuracy is only 71.0%.

Fig R3.2b shows the data flow of NAD. The input signal is the network traffic data modulated at the amplitude of the waveform with the speed of 128-G symbol/s. After the MRR convolution layer, the outputs are 128-Giga symbol/s signals but different from input. From the view of optical communication, they are "distorted" signals. But from the view of PNN, they are extracted features from the original signal. Then an FC layer takes them as input to generate 20

classification labels. By training the MRRs and FC layer together, the accuracy is 97.4%. In contrast, using the original signal and without the MRRs, the accuracy is only 93.7%.

Fig R3.2c shows the concept of Fashion-MNIST image classification. The input data is image pixel data modulated at the amplitude of the waveform with the speed of 128-Giga pixel/s. Using MRR works on different wavelengths, the input signals are mixed in both time and wavelength domain, corresponding to the 2D convolution. The output 128-Giga pixel/s signals can be reshaped to 2D feature images, which look different from the original image. By training the MRRs and FC layer together, the classification accuracy is 90.6%. In contrast, using the original image and without the MRRs, the accuracy is only 86.1%.

From the above illustration, we can conclude that: (1) Different from optical communication, we don't require distortion-free data transmission in PNNs. (2) The MRRs perform as effective convolution kernels and significantly increase the accuracy of PNN.

We believe the following point-by-point responses and corresponding manuscript revisions further clarify the mechanism of the proposed architecture and address all your concerns.

**Revision 1:**

1. In section "3. Discussion", line 384 to 393, we discuss the computation role of MRR temporal kernels and the difference between PNNs and optical communication system.

"By operating the system at a symbol rate exceeding the MRR resonance linewidth, we intentionally introduce temporal overlaps of neighboring symbols in the signals. In optical communication systems and conventional weight-based photonic computing systems, such overlap is known as inter-symbol inference, which is typically uncontrollable and regarded as an unwanted impairment that degrades symbol-level fidelity. However, in the proposed architecture, this effect becomes programmable and trainable computational operation induced by the MRR responses. It functions as a convolution operation in the PNNs, enabling the extraction of task-relevant features and contributing substantially to the network performance after training. Further discussion is detailed in Supplementary Note 10."

2. We provided the signals before and after MRR convolution and the corresponding classification accuracy in Supplementary Note 10.

<u>**Comment 2:**</u>

> Specific Comments:
>
> #1. High-Order Modulation and Actual Signal Bandwidth.
>
> The authors report a 120 Gbaud rate, which presumably involves high-order modulation (e.g., PAM4 or QAM) to achieve such throughput.
>
> Critical Requirement: The authors must provide the actual occupied bandwidth of the modulated signal and compare it directly with the MRR's resonance linewidth. If these two values are comparable, the filtering effect will induce severe phase and amplitude distortion. The authors should clarify how high-order symbols can be recovered with sufficient fidelity when the filter's edge-roll-off significantly clips the signal's high-frequency components.

**Response 2:**

Thank you for your comments regarding the relationship between symbol rate, signal bandwidth, and the MRR resonance linewidth.

First, we would like to clarify that we do not use high-order modulation formats to achieve 128 Gbaud speed. **128 Gbaud in our experiment is the actual symbol rate, not bit rate**. It is generated by a high-speed arbitrary waveform generator operating at 256 giga-sample/second.

Second, as we have clarified in **Response 1 that** the objective of our system is not high-speed signal communication or data transmission, but high-speed neural-network computation. **In optical communication systems**, the goal is to transmit each symbol with minimal distortion. Accordingly, the performance is typically evaluated by measuring the bit error rate (BER) between the transmitted and received signals. In this context, inter-symbol interference caused by insufficient channel bandwidth is regarded as an unwanted impairment that must be compensated or eliminated. **In contrast, in a neural network**, the objective is not to recover the exact input symbols, but to extract task-relevant features and generate inference outputs, such as classification labels. The performance is therefore evaluated using task-level metrics, such as classification accuracy, rather than symbol-level fidelity. In this context, interactions between neighboring symbols are not necessarily detrimental. Instead, they can serve as useful computational operations. Therefore, the local symbol interactions introduced by the bandwidth-limited response can be exploited as part of the computation rather than treated solely as transmission impairments.

Here we provide the details in the following section.

1. For modulation format identification (MFI), we experimentally use OOK and PAM4 signals at 32 Gbaud and 40 Gbaud, both generated by our high-speed arbitrary waveform generator, as shown in Fig R3.2a. After the MRR, the outputs are filtered signals look “distorted”. Rather than trying to recover the transmitted symbols from them, we extract the amplitude histograms from them and use an FC layer to generate four output labels, the maximum of which represents the classification results, that is, the modulation format. Using feature signals after the MRR convolution layer, the accuracy is 99.5%. In contrast, using the original signal, the accuracy is only 71.0%
2. For network anomaly detection (NAD), the network traffic data are encoded as the waveform amplitude and transmitted at 128-Giga symbol per second, as shown in Fig R3.2b. The experimental 128-Giga symbol-per- second traffic data is also generated by the high-speed arbitrary waveform generator. After the MRR convolution layer, the outputs are 128-Giga symbol/s signals look distorted from input and received by a high-speed photodetector and a high-speed real-time sampling scope at 256 Gsample/s. Then an FC layer takes them as input to generate 20 output labels, the maximum of which represents the classification results. Using feature waveforms after the MRR convolution layer, the accuracy is 96.4%. In contrast, using the original network traffic data, the accuracy is 93.7%.
3. For the Fashion-MNIST classification task, the reshaped image pixel values are encoded as the waveform amplitude at 128-Giga pixels per second, as shown in Fig R3.2c. The output 128-Giga pixel/s signals can be reshaped to 2D feature images, which looks distorted from the original image. Then FC layers take the feature images as input to generate 10 output labels, the maximum of which represents the classification results. Using the feature images after the MRR convolution layer, the accuracy is 90.6%, In contrast, using the original images, the accuracy is only 86.1%.

The reported 128-Gbaud operation refers to the symbol rate of the neural-network input stream rather than high-order communication modulation formats designed for distortion-free data recovery. This definition is consistent with prior works on high-speed PNNs [3,4]. Importantly, we don’t have to recover the input signals as in optical communication system. Instead, we used

the featured signal after the MRR convolution layer to obtain classification labels. Though the output signals look distorted from the input signal, they contain more task-relevant features and lead to higher classification accuracy. These results demonstrate that the bandwidth-limited filtering effect of the MRR is not an uncontrolled degradation mechanism, but rather a trainable and computationally useful temporal convolution process for PNNs.

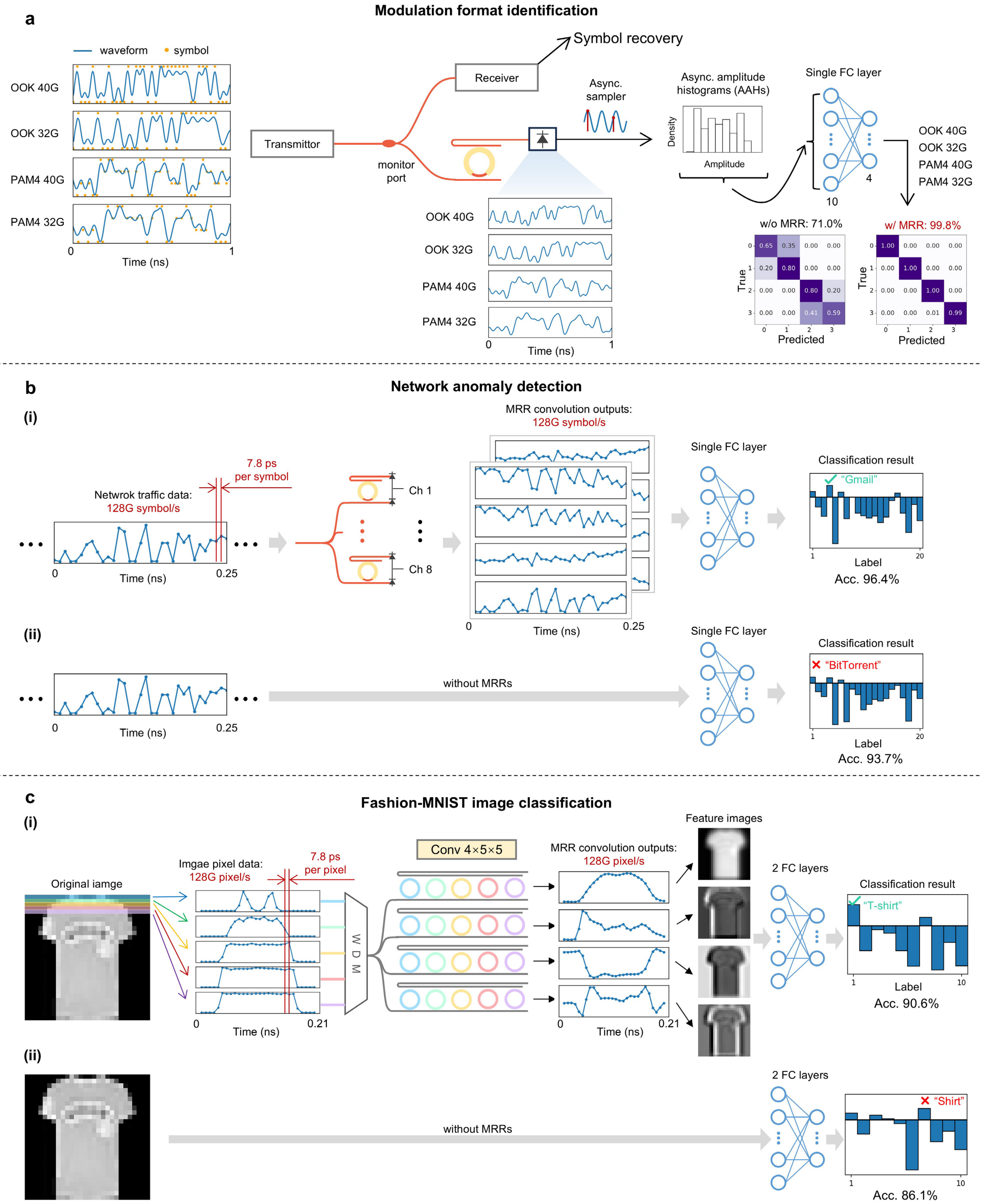


**Fig. R3.2 Data flow in PNNs.**

Following your suggestion, we additionally provide the frequency spectra of the input and filtered signals for the NAD task and compare them with the MRR resonance response, as shown in Fig. R3.3a–b. In Fig. R3.3a, the green curve represents the frequency response of the

MRR through port at zero frequency detuning. The blue and orange curves represent the power spectra of the input signal and the filtered output signal, respectively. Fig. R3.3b shows the corresponding result for the drop port. These results show that the signal spectrum is strongly reshaped by the MRR response because its bandwidth exceeds the resonance linewidth. In conventional optical communication systems, such spectral reshaping would typically be regarded as distortion or ISI because it prevents faithful recovery of the transmitted waveform. However, in the proposed PNN, this bandwidth-limited filtering effect is intentionally utilized as the convolution operation itself. In this example, at the through port, low-frequency components are suppressed while high-frequency components are preserved, corresponding to high-pass-like feature extraction. At the drop port, high-frequency components are suppressed while low-frequency components are preserved, corresponding to low-pass-like feature extraction. Consequently, the MRR functions as a programmable temporal feature extractor.

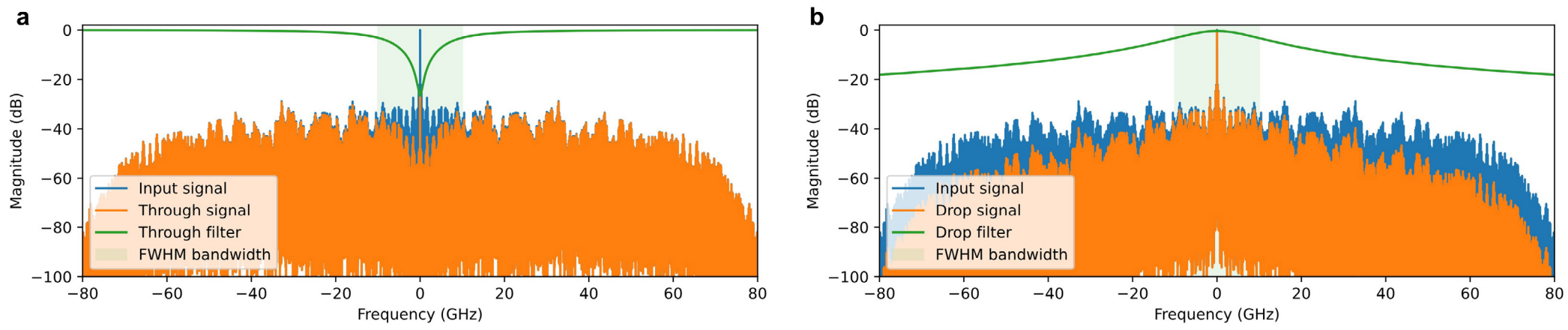


**Fig. R3.3 Comparison of signal bandwidth and MRR's resonance linewidth**

## Revision 2:

1. In section "3. Discussion", line 384 to 393, we discuss the computation role of MRR temporal kernels and the difference between PNNs and optical communication system.

"By operating the system at a symbol rate exceeding the MRR resonance linewidth, we intentionally introduce temporal overlaps of neighboring symbols in the signals. In optical communication systems and conventional weight-based photonic computing systems, such overlap is known as inter-symbol inference, which is typically uncontrollable and regarded as an unwanted impairment that degrades symbol-level fidelity. However, in the proposed architecture, this effect becomes programmable and trainable computational operation induced by the MRR responses. It functions as a convolution operation in the PNNs, enabling the extraction of task-relevant features and contributing substantially to the network performance after training. Further discussion is detailed in Supplementary Note 10."

2. We provided the signals before and after MRR convolution and the corresponding classification accuracy in Supplementary Note 10.

## Comment 3:

#2. Latency and Efficiency of the Training Phase

A major hidden bottleneck in MRR-based systems is the weight-tuning/training latency.

Operational Concern: To reach the "optimal state" for convolution, precise adjustment of the optical frequency (via thermal or carrier-injection detuning) is required. This is a time-consuming process, particularly when high-order modulation demands extreme precision to maintain a viable Bit Error Rate (BER). The authors should discuss the effective training overhead: if the time required to stabilize the resonance detuning is orders of magnitude longer than the inference time, the touted high-speed advantage is significantly compromised.

## Response 3:

Thank you for your comments regarding the training cost and resonance tuning latency in MRR-based photonic systems.

First, we would like to clarify the **distinction** between the **training/configuration stage** and the **inference stage** in artificial intelligence (AI) systems.

In AI systems, network parameters are first trained and optimized, but this training process is **typically performed only once**, after which the parameters are fixed for deployment. Therefore, the training or optimization time is not the main limiting factor in practical inference applications. For example, training a large language model may take months, but once the model is trained, user queries are answered through inference using the fixed network parameters, and the response time can be very short within a second. Similarly, in our system, the device parameters are optimized during the training/configuration stage and then kept fixed during operation. The actual AI inference is performed continuously afterward, and its speed is determined primarily by the hardware computing throughput rather than by the time required for parameter optimization or device configuration [5].

In the proposed architecture, once the MRR array is configured, the PNN can continuously perform inference with an input signal speed at 128 Gbaud, providing significantly higher computing throughput than conventional electronic hardware.

Here we provide the details of the MRR array **configuration time**, although we believe it is not a critical performance metric as we claim only high-speed inference rather than training. We use a calibration-free programming method named chip-in-the-loop (ChiL) [6], which is proposed in our previous work. This method features low time complexity and high programming precision in the presence of fabrication variations, thermal crosstalk, and temperature drift. Using ChiL, we can program the $8 \times 8$ MRR array simultaneously to the target detuning states with average wavelength error of 0.001 nm in only 5 optimization iterations, as shown in Fig R3.4. The total configuration time can be less than 20 ms using Field Programmable Gate Array (FPGA) [7].

After training, the system is deployed for inference and does not need to be changed. As shown in Fig R3.4. In our experiment, we continuously performed the NAD task for more than 5 hours at the input signal speed of 128 Gbaud and didn't observe accuracy degeneration.

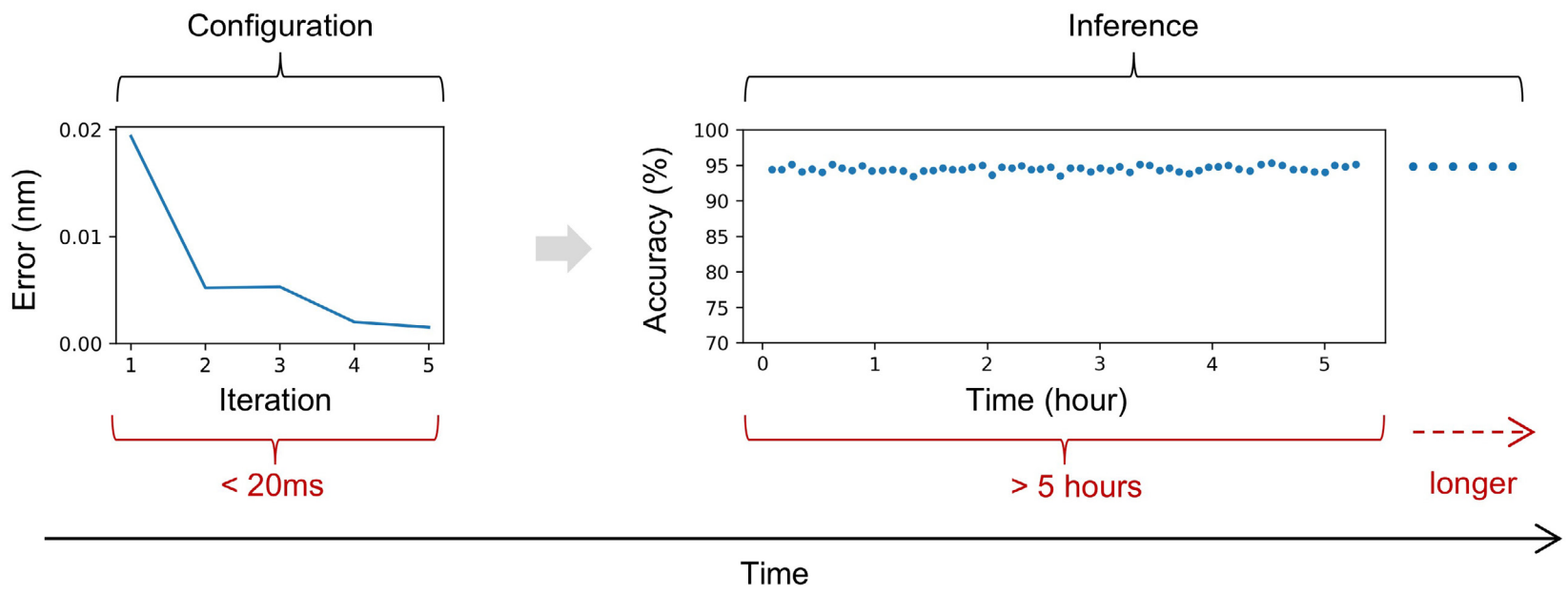


**Fig. R3.4 Configuration time and inference time in PNN**

## Revision 3:

We discuss the latency and efficiency of MRR configuration in Supplementary Note 14.

"Importantly, our method only uses 5 iterations to obtain this programming accuracy, as shown in Fig. S14c. The total configuration time can be less than 20 ms using Field Programmable Gate Array (FPGA)[12]. After that, the applied currents are fixed during inference stage. Therefore, the training or optimization time is not a limiting factor in high-speed inference applications."

## Comment 4:

#3. Determinism vs. Stochastic Distortion

In this scheme, "convolution" appears to rely on the temporal broadening of symbols. However, fabricated MRRs suffer from inherent process variations and thermal crosstalk. The authors must explain how they ensure this distortion remains deterministic and reproducible. Without a mechanism to compensate for individual device differences, the symbol overlapping becomes unpredictable, making it nearly impossible to transfer a trained model to different hardware instances or even to maintain stability during long-term operation.

## Response 4:

Thank you for your insightful comments regarding the determinism and reproducibility of the proposed PNN in the presence of fabrication variations and thermal crosstalk. We agree that these non-idealities exist in practical MRR systems and require clearer clarification.

In this work, we combine two methods to ensure robust and reproducible operation across different hardware instances and during long-term operation.

The first method is chip-in-the-loop (ChiL)[6], which is a calibration-free programming method proposed in our previous work for configuring large-scale MRR arrays under fabrication variations, thermal crosstalk, and environmental fluctuations. Using ChiL, we can accurately program the 8×8 MRR array. The frequency detuning errors exhibited a standard deviation of 0.138 GHz and a maximum value of 0.430 GHz, as shown in Fig. R3.5a.

The second method is sharpness-aware training (SAT)[8], which is proposed in our previous work to improve the robustness of PNNs against hardware imperfections. With SAT, the trained neural network maintains stable inference accuracy in the presence of resonance detuning errors.

As shown in Fig. R3.5b, the classification accuracy does not show noticeable degradation within ±1 GHz resonance frequency offset, which is more than 2× the maximum offset in the experiment. This result indicates that the trained model is robust against residual programming errors.

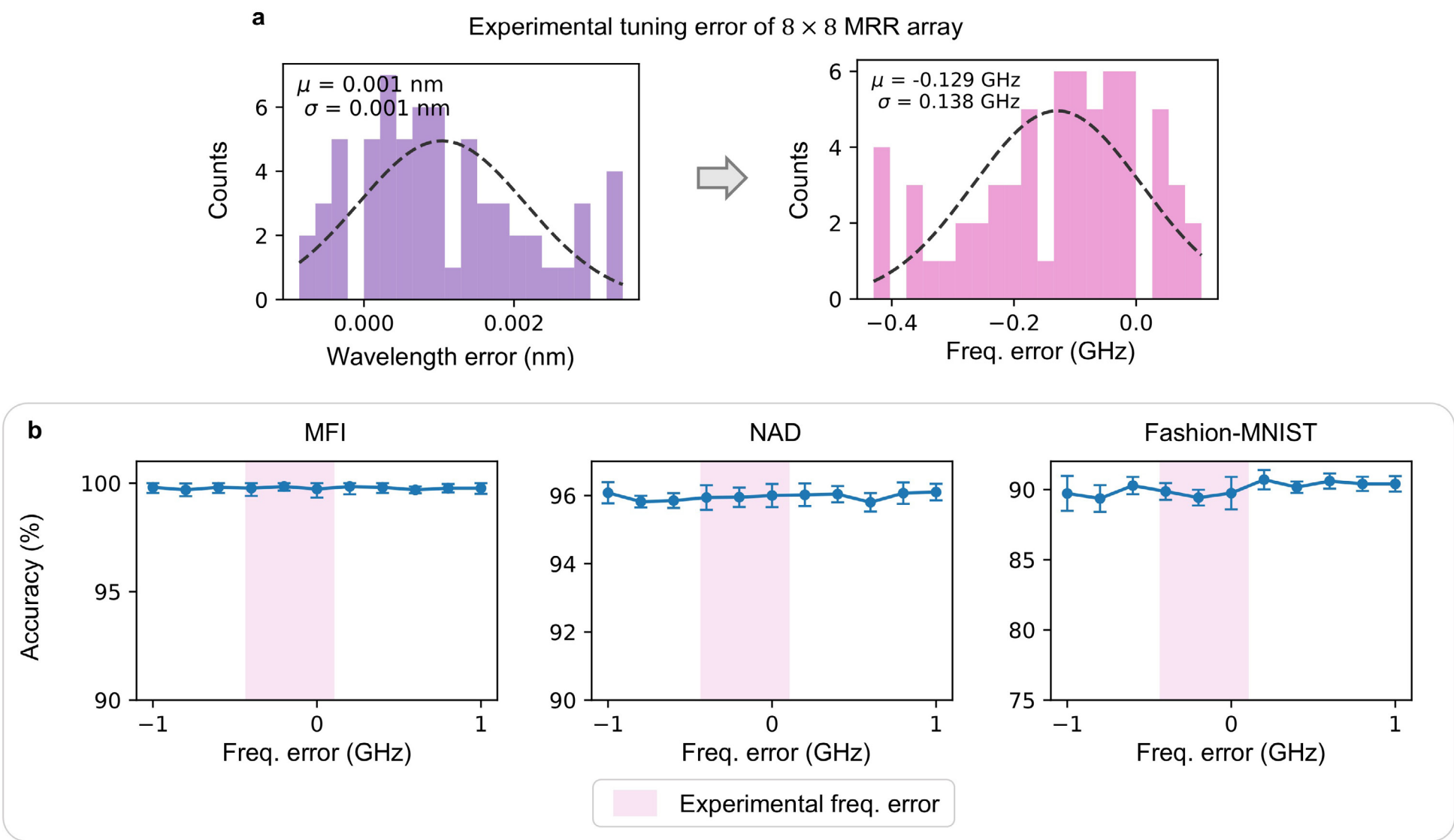


**Fig. R3.5 a** Experimental tuning error of 8 × 8 MRR array **b** Simulation results of classification accuracy vs. frequency tuning error.

In addition, SAT also improves robustness against fabrication-induced device variations. We extract the statistical distributions of the measured full-width-at-half-maximum (FWHM) bandwidth and extinction ratio of the 8×8 MRR array, as shown in Fig. R3.6a. Based on these, we evaluate the accuracy of PNNs with standard deviation of fabrication variations up to 2×. As shown in Fig. R3.6b, the accuracy remains largely unchanged across all three tasks. These results demonstrate that the trained model can be reliably transferred across different hardware instances.

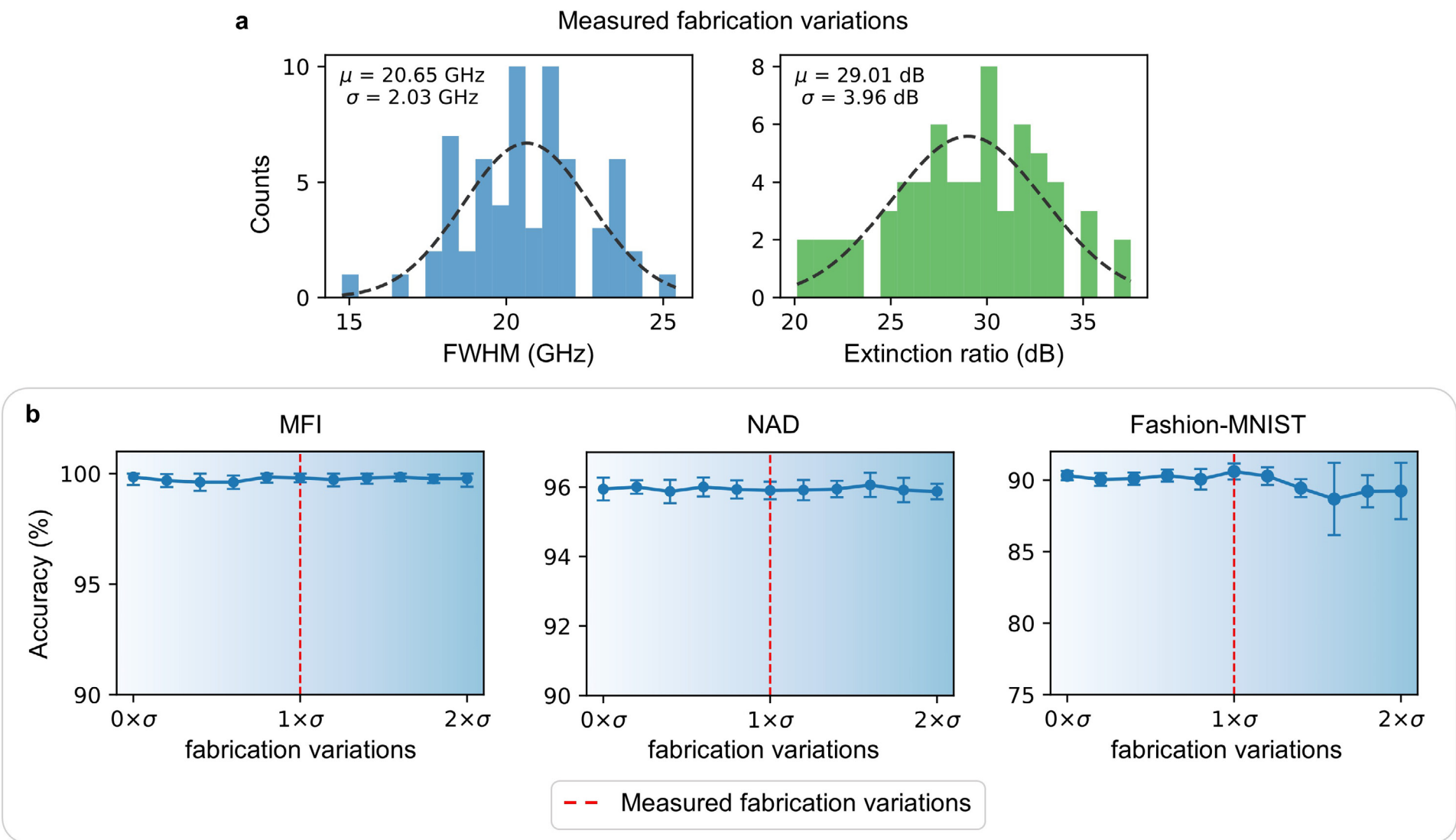


**Fig. R3.6 a** Measured fabrication variations of the 8 × 8 MRR array. **b** Simulation results of classification accuracy vs. fabrication variation factor.

To further address your concern on long-term operational stability, we additionally performed

continuous experiments on the NAD task over 5 hours of operation. The classification accuracy remains stable at around 95% without noticeable degradation, as shown in Fig. R3.7.

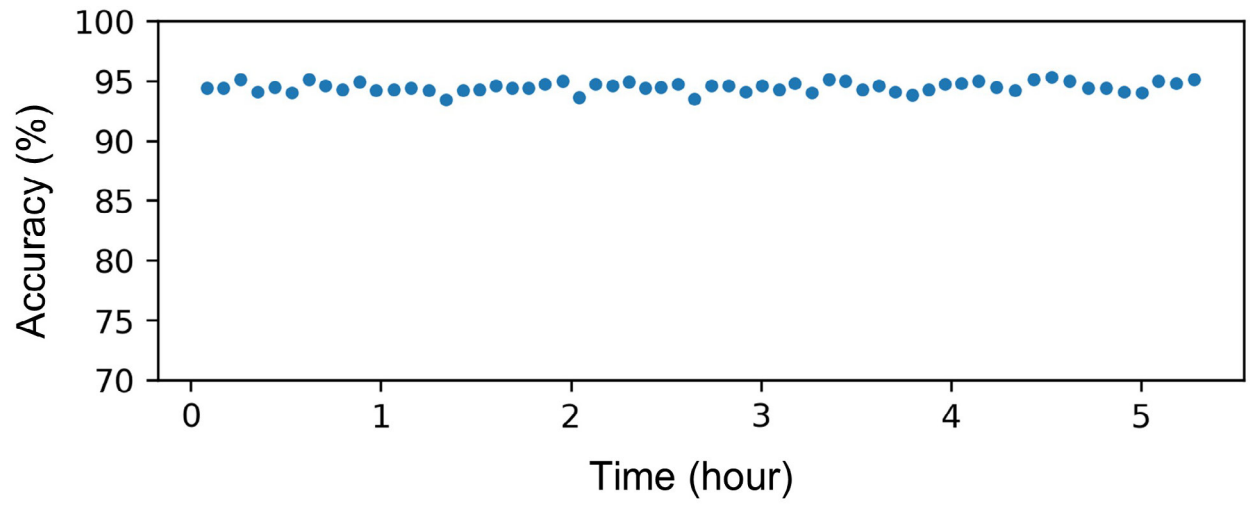


**Fig. R3.7** Long-term accuracy of the NAD task in experiment.

Therefore, although fabrication variations and thermal crosstalk exist in practical MRR systems, the proposed combination of ChiL programming and SAT enables deterministic, reproducible, and robust PNN operation across different devices and over long-term operations.

## Revision 4:

1. In section "2.2 Programming and training of the MRR convolution kernel" line 195 to 202, we mention using the chip-in-the-loop method to program the MRR array precisely in the presence of system imperfections:

"Accurate programming of large-scale MRR arrays is challenged by fabrication non-uniformity, thermal crosstalk, and environmental fluctuations. The characterizations of fabrication variations and thermal crosstalk coefficient are provided in Supplementary Note 12 and 13, respectively. To address these challenges, we employ the chip-in-the-loop approach for on-chip configuration. This method enables efficient and accurate programming of large-scale MRR arrays to the desired detuning positions while inherently accounting for fabrication imperfections and thermal crosstalk effects. (see Supplementary Note 14 for details)."

2. In section "3 Discussion", line 449 to 459, we discuss the robustness of the proposed PNN against fabrication variations and detuning errors.

"Photonic integrated circuits face practical challenges arising from fabrication variations and thermal crosstalk effects. Nevertheless, by employing the chip-in-the-loop method, all MRRs can be programmed to their target resonance detuning states with high precision, achieving a residual frequency offset of about 0.1 GHz (see Supplementary Note 14). Discrepancies may still exist between the actual MRR impulse responses and their digital models due to fabrication-induced variations in FWHM bandwidth and residual tuning errors. To address this problem, we employ sharpness-aware training to improve the robustness of PNNs against these imperfections. For the three tasks demonstrated, the PNNs maintain their accuracy under fabrication variations and detuning errors that are twice as large as those observed experimentally (see Supplementary Note 15)"

3. We provide analysis of robustness against detuning errors and fabrication variations in Supplementary Note 15.

4. In section "3. Discussion" line 462 to 466, we discuss the long-term operation accuracy and provide the experiment results in Fig S19.

"Stability is another important metric in analog computing systems. To evaluate long-term operational accuracy and stability, we continuously performed the NAD task over a period of 5 hours. The classification accuracy remained stable at approximately 95% throughout the

experiment, without any noticeable performance degradation (see Fig. S19)."

## Comment 5:

#4. Noise Analysis and SINAD in Low-Precision Regimes

The manuscript estimates a 5-bit precision, but lacks a formal SINAD (Signal-to-Noise and Distortion Ratio) analysis. High-speed operation combined with the sensitivity of high-order modulation makes the system extremely vulnerable to:

Thermal-induced resonance drift during long-term running; Accumulated noise from optical amplification (ASE) and high-speed electronic backends.

The authors should provide experimental or simulation data on the long-term stability of the SINAD, particularly how the system maintains its claimed accuracy under continuous operational heating.

## Response 5:

Thank you for your insightful comments regarding resonance drift, noise, and the stability of the proposed PNN under long-term operation.

Regarding resonance drift and long-term stability, as we present in Response 4, Fig. R3.5b shows that the PNN maintains stable accuracy within ±1 GHz resonance frequency offset, demonstrating its tolerance against resonance drift caused by thermal fluctuations. In addition, Fig. R3.7 presents long-term experimental measurements over 5 hours of continuous operation, where the classification accuracy remains stable without noticeable degradation.

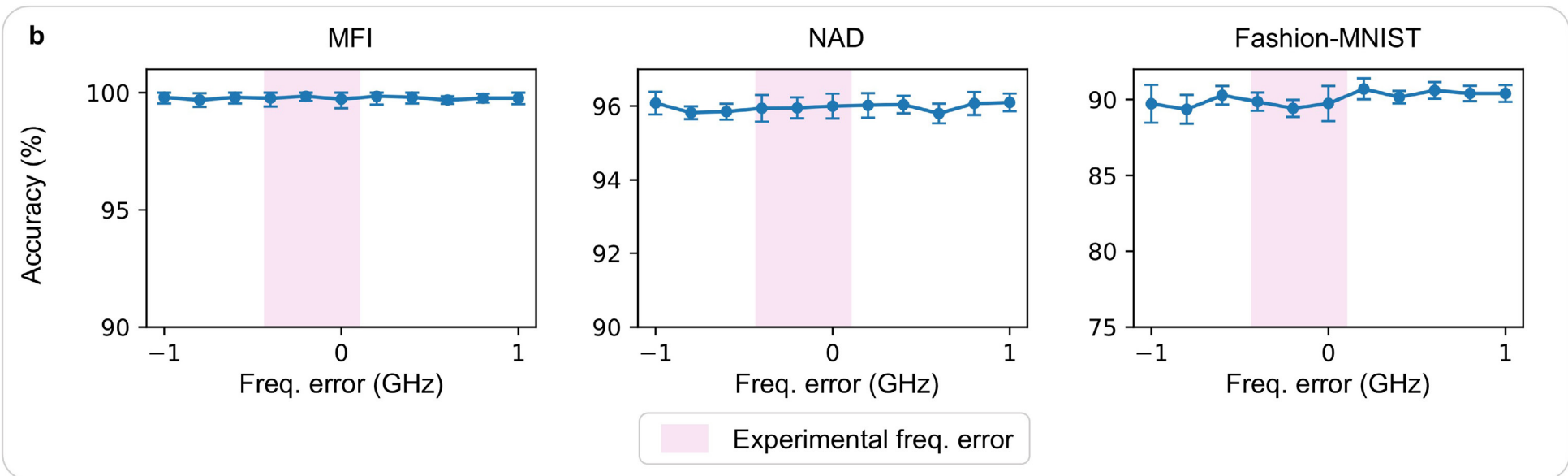


**Fig. R3.5 b** Simulation results of classification accuracy vs. frequency tuning error.

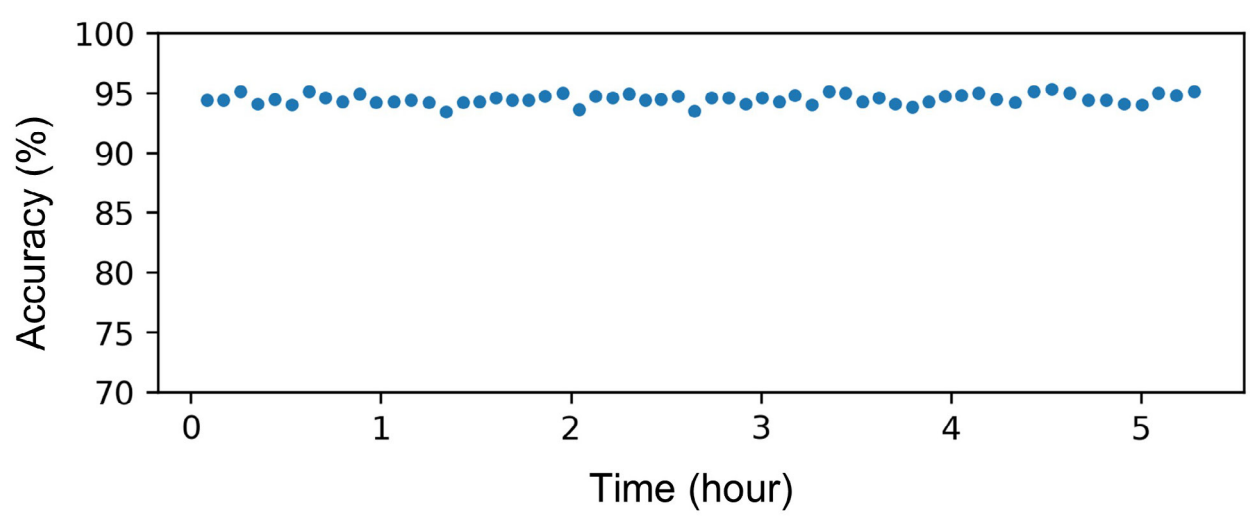


**Fig. R3.7** Long-term accuracy of the NAD task in experiment.

To further address your concern on the tolerance of the proposed PNN against noise and distortion, we additionally perform simulations under different SNR and computing precision

for all three demonstrated tasks. The SNR is defined as:

$$\text{SNR (dB)} = 10\log_{10}\frac{<\hat{y}^2>}{<(y-\hat{y})^2>} \qquad (R3-1)$$

where $\hat{y}$ represents simulated computing results, $y$ represents experimental computing results with noise and distortion, and $<\cdot>$ represents the average operation. The computing precision is defined as

$$\text{Precision (bit)} = \log_2\frac{\max(\hat{y})-\min(y)}{\sqrt{<(y-\hat{y})^2>}} \qquad (R3-2)$$

The simulation results are shown in Fig. R3.8. For the MFI task, the classification accuracy remains above 99% at SNR of 20 dB and computing precision of about 4.4 bits. For the NAD task, the accuracy remains above 95% at SNR of 14 dB and computing precision of about 4.4 bits. For the Fashion-MNIST classification task, the accuracy remains above 89% at SNR of 13 dB and computing precision of about 4.2 bits. These results indicate that the proposed PNN can maintain stable inference performance under practical noisy operating conditions.

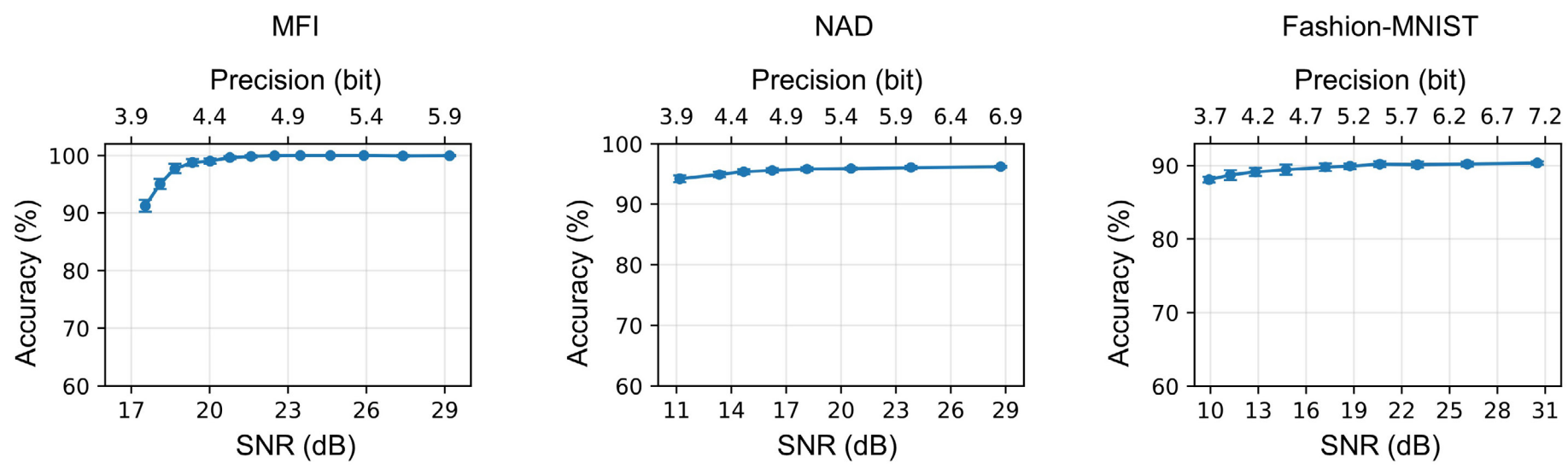


**Fig. R3.8** Simulation results of classification accuracy vs. SNR and computing precision

The robustness against resonance drift, distortion, and accumulated noise mainly benefits from the training strategy adopted in this work. We combine sharpness-aware training (SAT) [8] and noise-injection training [9] to improve the tolerance of the PNN against hardware imperfections and stochastic noise. SAT improves robustness against device variations and resonance perturbations, while noise-injection during training improves tolerance against noise introduced by optical amplification, photodetection, and high-speed electronic backends. As a result, the trained neural network preserves stable accuracy even in the presence of resonance drift and noisy long-term operation.

## Revision 5:

1. In section "3 Discussion", line 459 to 461, we discuss the robustness of the proposed PNN against signal-to-noise ratio.

"In addition, by incorporating noise and timing errors during training, the PNNs can work accurately and stably in the presence of noise and timing jitter in the high-speed analog computing system (see Supplementary Note 16 and 17)."

2. We provide simulation results of accuracy with SNR and computing precision in Supplementary Note 16.

## Comment 6:

#5. Practical Benchmarking for Convolutional Advantages

The MNIST task is insufficient to demonstrate the utility of 120 Gbaud time-domain convolution. The community requires evidence that this scheme can handle tasks where temporal correlation is essential, such as high-order nonlinear impairment compensation in fiber-optic links or complex time-series forecasting. The authors need to demonstrate that this hardware can outperform conventional digital or weighting-based photonic processors in tasks that specifically demand high-speed temporal integration.

## Response 6:

Thank you for your insightful comments regarding the applicability of the proposed MRR temporal convolution architecture to tasks that require strong temporal correlation and high-speed temporal integration. To further address your concerns, we additionally demonstrate two representative tasks where temporal correlation is essential.

The first task is fiber-optic communication signal equalization. We use the proposed MRR temporal convolution for equalizing 120 Gbaud PAM4 signals after 80-km C-band optical fiber transmission in intensity-modulation direct-detection (IMDD) system. The simulation setting is shown in Fig. R3.9a. The optical fiber is modeled as standard single-mode fiber (SSMF) with chromatic dispersion coefficient $D = 17\ \mathrm{ps/nm/km}$ and nonlinear coefficient $\gamma = 1.3\ \mathrm{W^{-1}km^{-1}}$. During transmission, the signal experiences chromatic dispersion and nonlinear impairments of self-phase modulation, which introduce severe temporal distortion and inter-symbol interference, making temporal equalization essential for signal recovery. The results in Fig. R3.9b show that without equalization, the bit-error-rate (BER) is $4.63 \times 10^{-1}$. Using a conventional 1500-tap feed-forward equalizer (FFE), the BER is reduced to $9.50 \times 10^{-2}$, which is significantly higher than the hard-decision forward-error-correction (HD-FEC) threshold. A nonlinear digital convolutional neural network (CNN) provides a similar BER of $9.21 \times 10^{-2}$. In contrast, by using two MRR temporal convolution kernels before the photodetectors, the BER is reduced below the HD-FEC threshold to $8.09 \times 10^{-4}$ with only $2 \times 161$-tap FFE, which is below the HD-FEC threshold. The eye diagrams before and after equalization are shown in Fig. R3.9d. Without equalization, the eye diagram is nearly closed due to ISI, whereas MRR equalization significantly opens the eye, indicating substantially improved signal quality. What's more, the proposed MRR-based equalizer requires significantly fewer digital parameters compared with conventional FFE and digital CNN approaches, as shown in Fig. R3.9c. These results demonstrate that the proposed temporal convolution architecture can handle high-order nonlinear impairment compensation tasks, outperforming conventional digital signal processing methods.

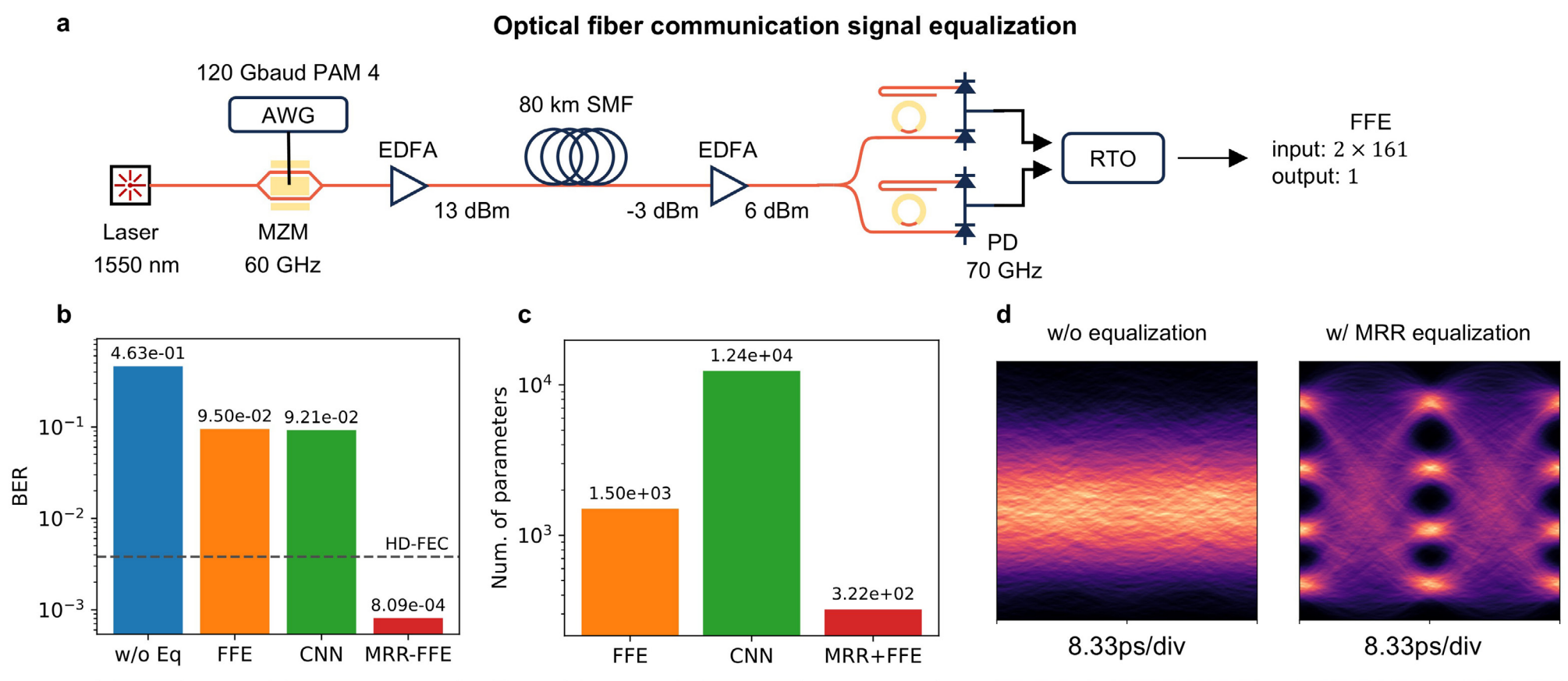


**Fig. R3.9** MRR temporal convolution kernel for optical fiber communication signal equalization

The second task is nonlinear time-series prediction using the Santa Fe Laser dataset, which is generated from a chaotic system with unknown dynamics and widely used for nonlinear sequence prediction [10,11]. We use the data from t-7 step to t step to predict the data of t+1 step. The network architecture is shown in Fig. R3.10a, consisting of an 8-channel MRR temporal convolution layer and a FC layer for prediction. The simulation results are shown in Fig. R3.10b. When operating at 128 Gbaud, each MRR functions as a temporal convolution kernel for extracting time-domain features from the sequence, and the proposed MRR-based convolution neural network achieves a normalized mean-square-error (NMSE) of 0.012. In contrast, when the symbol rate is 1 Gbaud, MRRs work as scalar weights, resulting in NMSE of 0.069, which is close to the NMSE (0.079) of using the FC layer only. These results indicate that the proposed temporal convolution architecture provides substantially stronger temporal feature extraction capability than conventional weighting-based photonic processors.

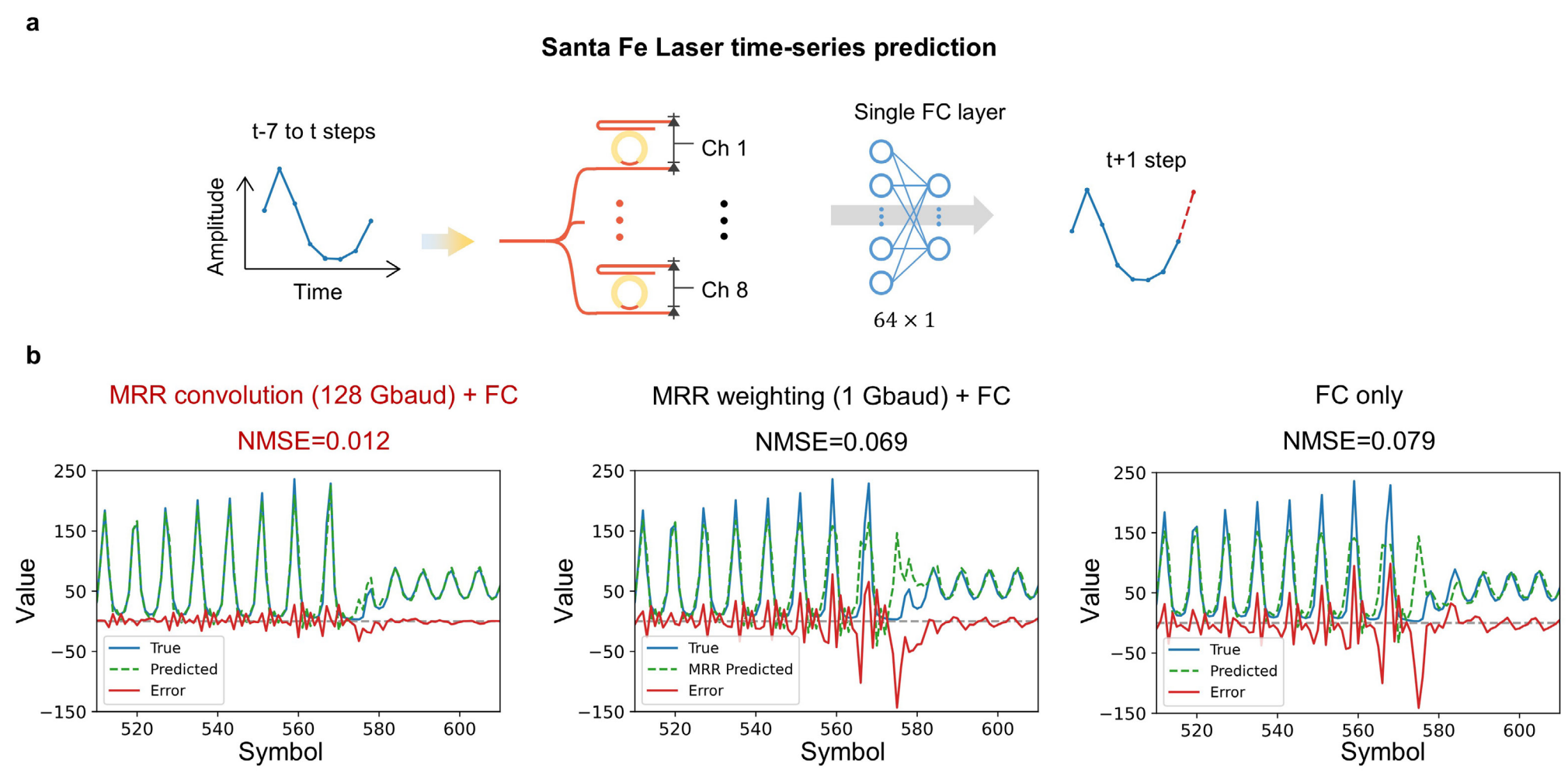


**Fig R3.10** MRR temporal convolution kernel for Santa Fe Laser nonlinear time-series prediction

Therefore, the proposed architecture can effectively address tasks that fundamentally rely on temporal correlation and high-speed temporal integration, including optical communication equalization and nonlinear time-series prediction.

## Revision 6:

1. In section "3 Discussion" line 468 to 490, we discuss the applications of the proposed MRR kernels for optical communication signal processing and nonlinear time-series prediction.

"The proposed MRR temporal convolution kernels serve as a versatile computational primitive for photonic computing. Beyond the classification tasks, we further investigate their applicability to optical communication signal processing and nonlinear time-series prediction through numerical simulations.

For optical communication signal processing, we demonstrate using an MRR-based photonic equalizer for 120 Gbaud PAM4 signal across 80-km single-mode fiber transmission at C-band. Using 2 MRR temporal kernels and $2 \times 161$-tap feed-forward equalizer (FFE), the bit error rate (BER) is $8.09 \times 10^{-4}$, far below the hard-decision forward-error-correction (HD-FEC) threshold of $3.8 \times 10^{-3}$. For comparison, a digital convolutional neural network containing over 38× trainable parameters achieves a BER of $9.21 \times 10^{-2}$, substantially above the HD-FEC limit. The superior performance of the MRR-based equalizer arises from its ability to perform complex-valued convolution directly on complex optical fields prior to photodetection, thereby preserving phase information that is inaccessible to conventional intensity-domain DSP approaches. More details are provided in Supplementary Note 20.

We further evaluate the capability of the MRR temporal kernel for nonlinear time-series prediction. For predicting Santa Fe Laser data, PNN based on the MRR temporal kernels achieves a normalized mean-square error (NMSE) of 0.012 when operating at 128 Gbaud. In contrast, when operating at 1 Gbaud, MRRs degenerate to scalar weighting elements and result in NMSE of 0.069. More details are provided in Supplementary Note 21. These results highlight the strong temporal feature-extraction capability of the proposed MRR kernels and demonstrate their effectiveness beyond classification tasks."

2. We provide the simulation setting and results of optical communication signal equalization and Santa Fe Laser time-series prediction in Supplementary Note 20 and Supplementary Note 21.

**Reference:**


1. Krizhevsky, A., Sutskever, I. & Hinton, G. E. ImageNet Classification with Deep Convolutional Neural Networks. in *Advances in Neural Information Processing Systems* vol. 25 (Curran Associates, Inc., 2012).
2. Feldmann, J. *et al.* Parallel convolutional processing using an integrated photonic tensor core. *Nature* **589**, 52–58 (2021).
3. Lin, Z. *et al.* 120 GOPS Photonic tensor core in thin-film lithium niobate for inference and in situ training. *Nat Commun* **15**, 9081 (2024).
4. Zhang, X. *et al.* Unlocking High-Speed and Energy-Efficiency: Integrated Convolution Processing on Thin-Film Lithium Niobate. *Laser & Photonics Reviews* **19**, 2401583 (2025).
5. McMahon, P.L. The physics of optical computing. *Nat Rev Phys* **5**, 717–734 (2023)
6. Liu, S. *et al.* Calibration-free and precise programming of large-scale ring resonator circuits. *Optica, OPTICA* **12**, 1113–1121 (2025).
7. Zhang, W. *et al.* A system-on-chip microwave photonic processor solves dynamic RF interference in real time with picosecond latency. *Light Sci Appl* **13**, 14 (2024).
8. Xu, T. *et al.* Physical neural networks using sharpness-aware training. *Nat Commun* **17**, 1766 (2026).
9. Mourgias-Alexandris, G. *et al.* Noise-resilient and high-speed deep learning with coherent silicon photonics. *Nat Commun* **13**, 5572 (2022).
10. Weigend, A. S. & Gershenfeld, N. A. Results of the time series prediction competition at the Santa Fe Institute. in *IEEE International Conference on Neural Networks* 1786–1793 vol.3

(1993). doi:10.1109/ICNN.1993.298828.
11. Wang, D., Nie, Y., Hu, G., Tsang, H. K. & Huang, C. Ultrafast silicon photonic reservoir computing engine delivering over 200 TOPS. *Nat Commun* **15**, 10841 (2024).

Dear reviewers,

Thank you for taking the time to review our manuscript NCOMMS-26-024440A. We deeply appreciate your valuable comments and constructive suggestions. We have carefully revised the manuscript accordingly and highlighted the modifications in blue.

Detailed point-by-point responses and revisions are provided below:

## Response to Reviewer #1

**Comment 1:**

All my previous comments are addressed. I have no further comments and would like to recommend this article for publication.

**Response 1:**

Thank you again for your valuable comments and support.

## Response to Reviewer #2

**Comment 1:**

The authors have addressed most of my concerns. However, I still suggest the authors change the phrase 'beyond 100 GHz clock rate' in the title to be 'beyond 100 Gbaud' to avoid ambiguity since they actually demonstrated a computing speed of 128 Gbaud and using GHz to represent speed is not very standardized. By the way, some words 'Gbaud' were incorrectly written as 'Gbuad'.

**Response 1:**

Thank you for your thoughtful comments. We had changed the title to "Microrings as programmable temporal kernels enabling photonic AI beyond 100 Gbaud" according to your suggestions. We have also corrected the typo mistake of "Gbaud".

## Response to Reviewer #3

**Comment 1:**

All my concerns have been revised. Accept as is.

**Response 1:**

Thank you again for your valuable comments and support.
