# Supplementary material for: Microrings as programmable temporal kernels enabling photonic AI beyond 100 Gbaud
Source: Nat Commun. 2026 Sep 25;17:11107. doi: 10.1038/s41467-026-77446-8 (PMC13696697; doi:10.1038/s41467-026-77446-8)
Supplement: Supplementary file 2 [file 41467_2026_77446_MOESM2_ESM.pdf]

Supplementary Information for:

Microrings as programmable temporal kernels enabling photonic AI beyond 100 Gbaud

Shaojie Liu[1], Tengji Xu[1], Benshan Wang[1], Jiayong Peng[1], Qiarong Xiao[1], Li Fan[1], Hongwei Chen[2,3], and Chaoran Huang[1*]

1. Department of Electronic Engineering, The Chinese University of Hong Kong, Shatin, Hong Kong SAR, China
2. Department of Electronic Engineering, Tsinghua University, Beijing, China
3. Beijing National Research Center for Information Science and Technology (BNRist), Beijing, China.

*Corresponding author E-mail: crhuang@ee.cuhk.edu.hk

Contents

Supplementary Note 1. MRR characterization 2
Supplementary Note 2. Discrete MRR convolution model 2
Supplementary Note 3. Experimental setup and system bandwidth characterization .. 4
Supplementary Note 4. Training of the photonic neural network 4
Supplementary Note 5. Computing precision 6
Supplementary Note 6. Extend the MRR convolution engine to higher dimensions...... 6
Supplementary Note 7. Single-chip integrated computing system design 7
Supplementary Note 8. Energy efficiency estimation 8
Supplementary Note 9. Comparison of PNNs with digital neural networks 9
Supplementary Note 10. Data flow in PNNs versus optical communication systems..... 10
Supplementary Note 11. High-speed advantage for the temporally uncorrelated task ..11
Supplementary Note 12. Fabrication variations characterization 12
Supplementary Note 13. Thermal crosstalk characterization 12
Supplementary Note 14. Programming of MRR array 13
Supplementary Note 15. Robustness against fabrication variations and tuning error .. 15
Supplementary Note 16. Noise and computing precision tolerance 15
Supplementary Note 17. Timing error tolerance 16
Supplementary Note 18. Long-term operation accuracy and stability 17
Supplementary Note 19. MRRs for image classification without FC layers 17
Supplementary Note 20. MRRs for Optical communication signal equalization 18
Supplementary Note 21. MRRs for Nonlinear time-series prediction 19

Supplementary Note 1. MRR characterization

The spectrum of an MRR is enlarged and shown in Supplementary Figure 1a. The frequency axis is normalized to the resonance frequency. From the measured spectrum, the full width at half maximum (FWHM) bandwidth is approximately 20 GHz, the extinction ratio is about 27 dB, and the Q factor is approximately 10^4. By fitting the resonance curve, we extract the self-coupling coefficients $t_1 = t_2 = 0.96$ and the round-trip loss factor $a = 0.996$.

The tuning efficiency of the MRR is characterized by applying different currents to the thermo-optic phase shifter while performing a spectral sweep at each current, as shown in Supplementary Figure 1b. The resistance of the phase shifter is measured to be approximately 200 Ω. The resonance frequency shift as a function of the applied electrical power is shown in Supplementary Figure 1c, from which a tuning efficiency of 6.95 GHz/mW is obtained.

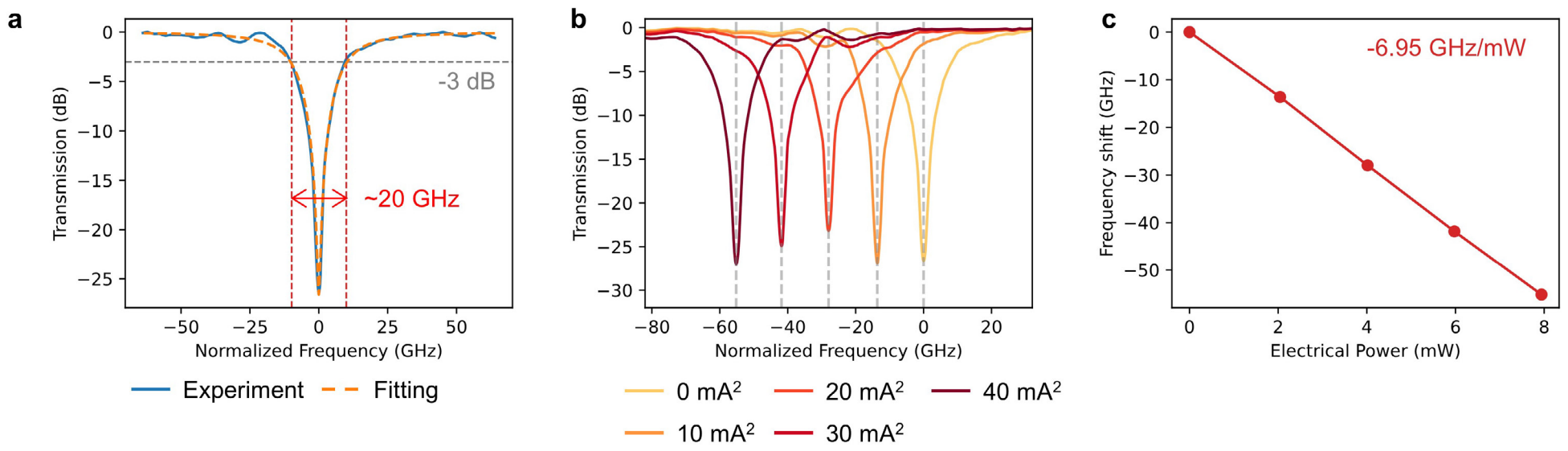

Supplementary Figure 1 MRR characterization. a Through port transmission spectrum of an MRR. **b** Transmission spectrum of the MRR under different drive currents. **c** Resonance frequency shift versus electrical power.

Supplementary Note 2. Discrete MRR convolution model

The continuous-time impulse response of the through ($h_T(t)$) and drop ($h_D(t)$) ports of the MRR is described in Eqs. (3)-(4) in the manuscript. In neural networks, convolution is typically defined in discrete time. For a signal sampled at a symbol rate B, the discrete convolution kernel $h[n]$ of the MRR can be approximated as Eq. (S1):

$$h[n] = \int_{\frac{n-1}{B}}^{\frac{n}{B}} h(t)\,\mathrm{d}t\,, n = 1,2,\dots,N \tag{S1}$$

The continuous-time impulse response $h(t)$ and discrete-time convolution kernel $h[n]$ of the drop port of the MRR from numerical simulations are shown in Supplementary Figure 2. The simulation parameters are extracted from the characterization results described in Supplementary Note 1. We define the effective kernel size N as the largest index satisfying $|h[n]| > 0.1\,|h[1]|$. As shown in Supplementary Figure 2, the effective kernel size is 5 for a 128 Gbaud signal and 3 for a 64 Gbaud signal.

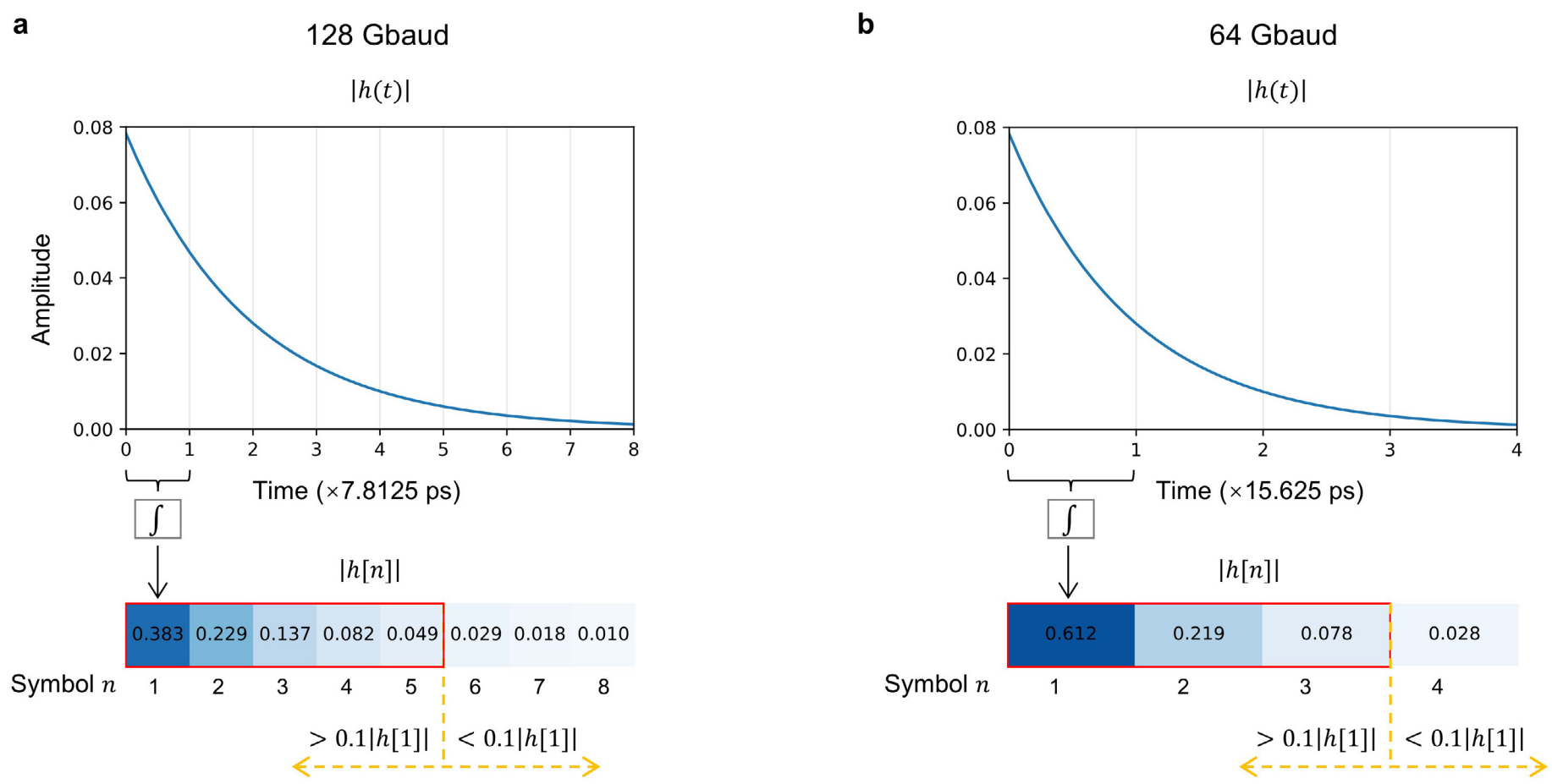

Supplementary Figure 2 Impulse response and convolution kernel of the MRR (drop port).

The energy outside the effective kernel size contributes marginally to the convolution results, which can be validated by testing the accuracy of the PNN using different truncated kernel sizes. We test the accuracy of NAD tasks at 128 Gbaud using different N in time-domain simulations. As shown in Supplementary Figure 3, accuracy remains high and nearly unchanged for $N \geq 5$, indicating that a kernel truncated at $N = 5$ accurately captures the convolution behavior of the MRR. In contrast, the accuracy decreases significantly when $N < 5$, indicating that a kernel truncated at $N < 5$ cannot capture the convolution behavior. These results indicate that the kernel coefficients within $1 \leq n \leq 5$ dominate the convolution process, while the coefficients for $n > 5$ contribute only marginally to the convolution result. Therefore, defining an effective kernel size of 5, corresponding to the truncation threshold of 0.1, is reasonable.

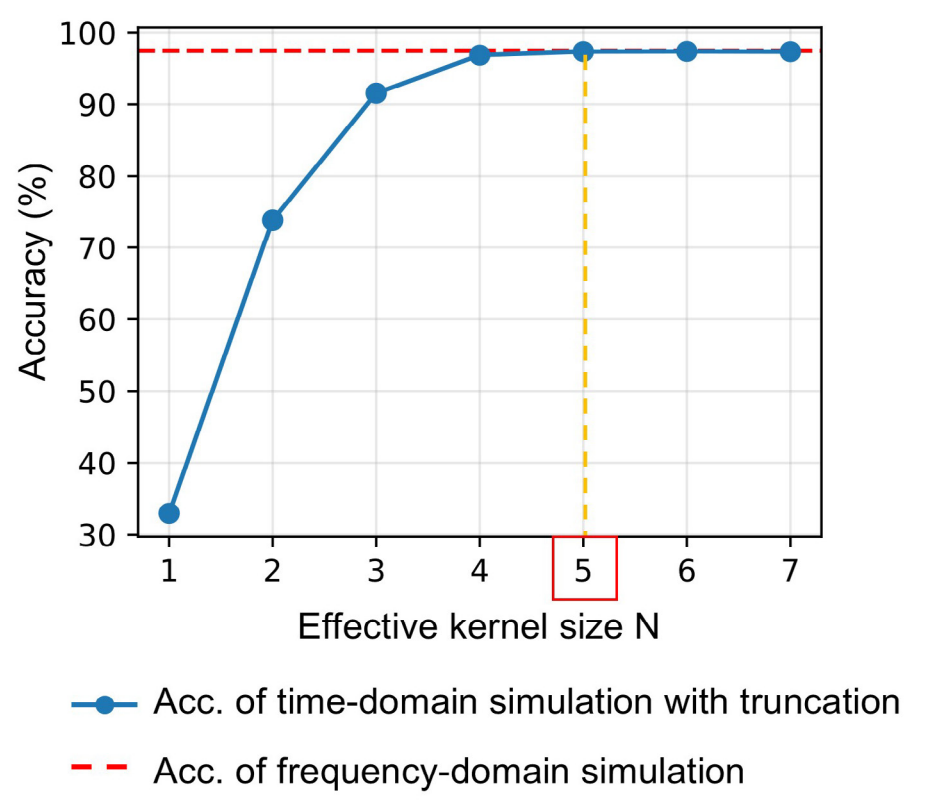

Supplementary Figure 3 Accuracy vs. effective kernel size for the NAD task at 128 Gbaud.

Notably, $h[n]$ is solely a mathematical model to facilitate comparison with digital CNNs. In the actual neural-network training process, we do not explicitly truncate the MRR impulse response. Instead, we model the MRR convolution effect in the frequency domain, as described in Supplementary Note 4. Therefore, the full response of the MRR is naturally included in training without applying hard truncation, thereby closely approximating the real-world physical system. The red dashed line in Supplementary Figure 3 shows the classification accuracy obtained from the frequency-domain simulation. Its performance matches that of the time-domain simulation when $N = 5$, providing additional justification for defining the truncation threshold of 0.1.

Supplementary Note 3. Experimental setup and system bandwidth characterization

The experimental setup used in this work is shown in Supplementary Figure 4a, and a photograph of the packaged chip is presented in Supplementary Figure 4b. A multi-tone signal is used to characterize the bandwidth of the entire signal link (Supplementary Figure 4c), showing a 3-dB bandwidth of approximately 38 GHz.

In the experiments on modulation format identification (MFI) and network anomaly detection (NAD), the laser wavelength is fixed at 1550 nm, and the MRRs with a radius of 15 μm are used. For the Fashion-MNIST image classification experiment, wavelengths ranging from 1550 nm to 1553.2 nm with a spacing of 0.8 nm are employed, together with MRRs whose radii range from 15 μm to 15.04 μm with a spacing of 0.01 μm.

Due to the limited number of high-speed instruments, experiments at different wavelengths are performed sequentially and the results are digitally summed to emulate two-dimensional (2D) image convolution. Since optical signals at different wavelengths are mutually incoherent, this approach introduces negligible differences compared with fully parallel optical convolution and is sufficient for proof-of-concept demonstrations.

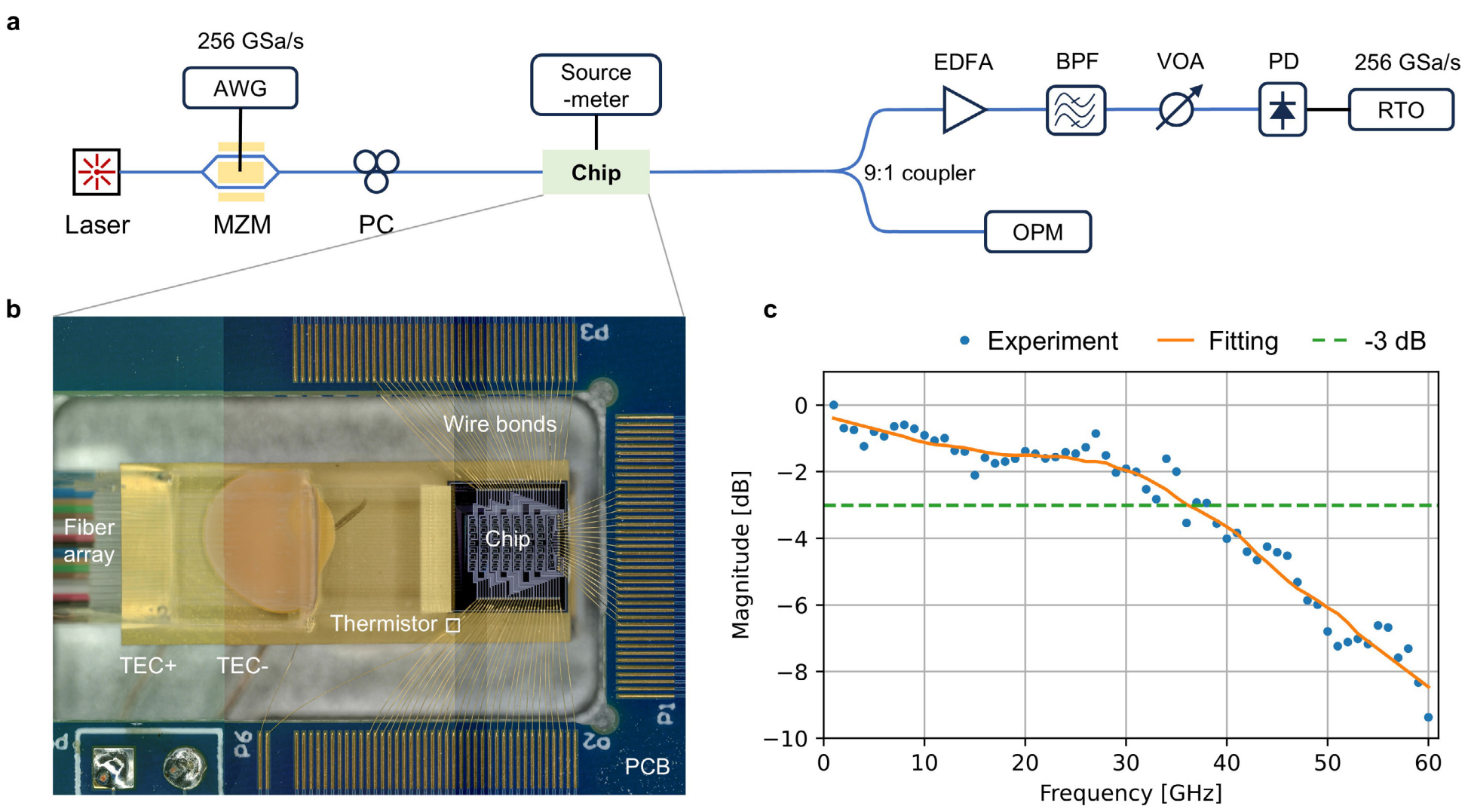

Supplementary Figure 4 Experimental setup and system bandwidth characterization. a Experimental setup. **b** Photo of the packaged chip. **c** System bandwidth characterization. AWG: arbitrary waveform generator. MZM: Mach–Zehnder modulator. PC: polarization controller. EDFA: erbium-doped fiber amplifier. BPF: band-pass-filter. VOA: variable optical attenuator. PD: photodetector. OSC: real-time oscilloscope. OPM: optical power meter.

Supplementary Note 4. Training of the photonic neural network

In this work, the photonic neural network (PNN) is trained in silico using the PyTorch framework[1]. The concept of training and inference is illustrated in Supplementary Figure 5. The trainable parameters in the physical system are the resonance frequency shifts of the MRRs.

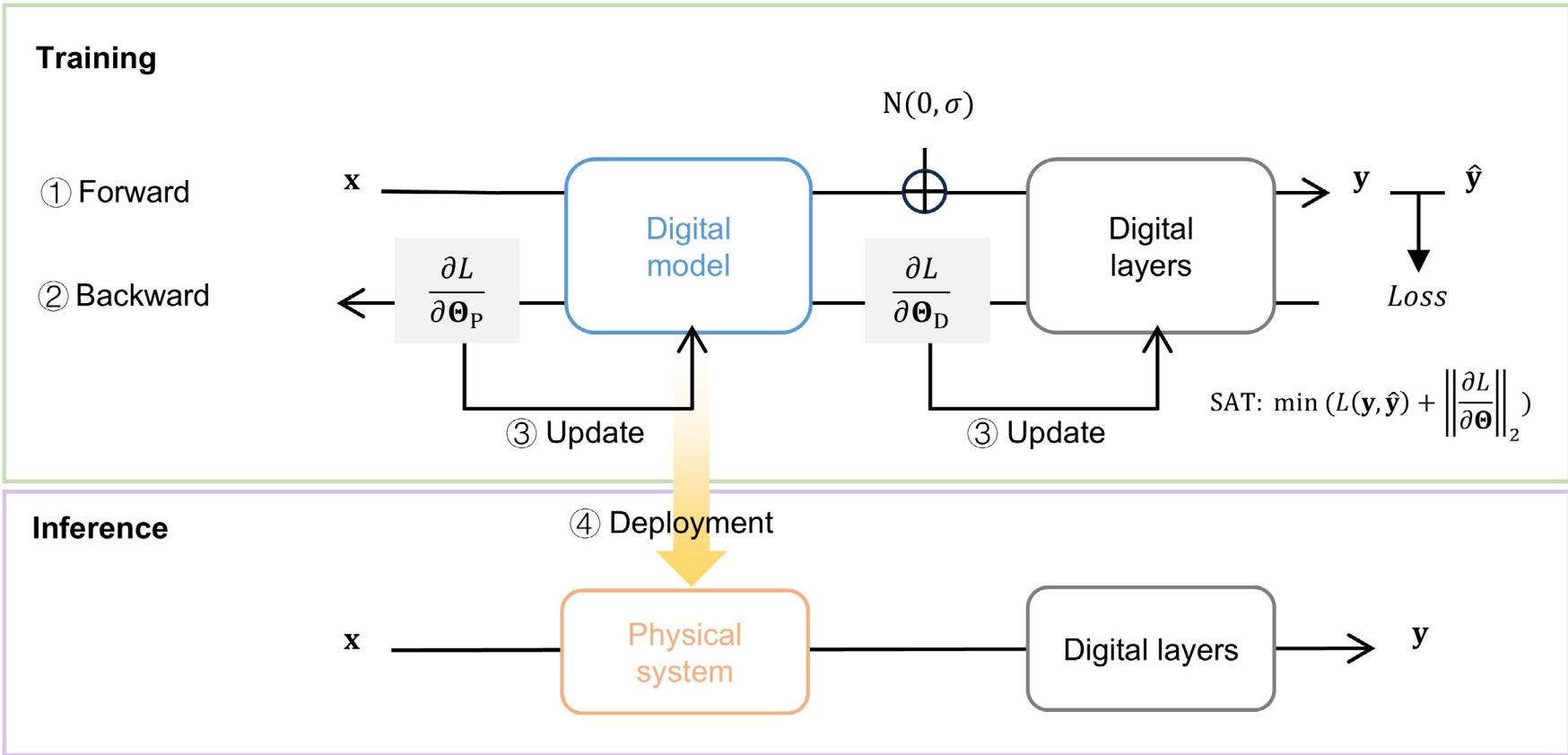

Supplementary Figure 5 Training of the photonic neural network.

During training, a differentiable digital model of the physical system is constructed. The model incorporates the responses of the electro-optic modulator, MRRs, photodetectors, and system bandwidth limitations. By using bandwidth-aware training[2], it is not required to use pre-emphasis, equalization, or partial-response signaling to compensate for the ISI during transmission, which would introduce additional digital signal processing (DSP) overhead during both training and inference. Instead, the bandwidth limitation is treated as an intrinsic part of the analog photonic computing system. Here we perform simulations to compare the performance of the NAD task with and without bandwidth-aware training. As shown in Supplementary Figure 6, with bandwidth-aware training, the inference accuracy remains above 95% when the system bandwidth varies from 30 GHz to 40 GHz. In contrast, without bandwidth-aware training, the inference accuracy decreases to below 90% when the bandwidth is lower than 40 GHz.

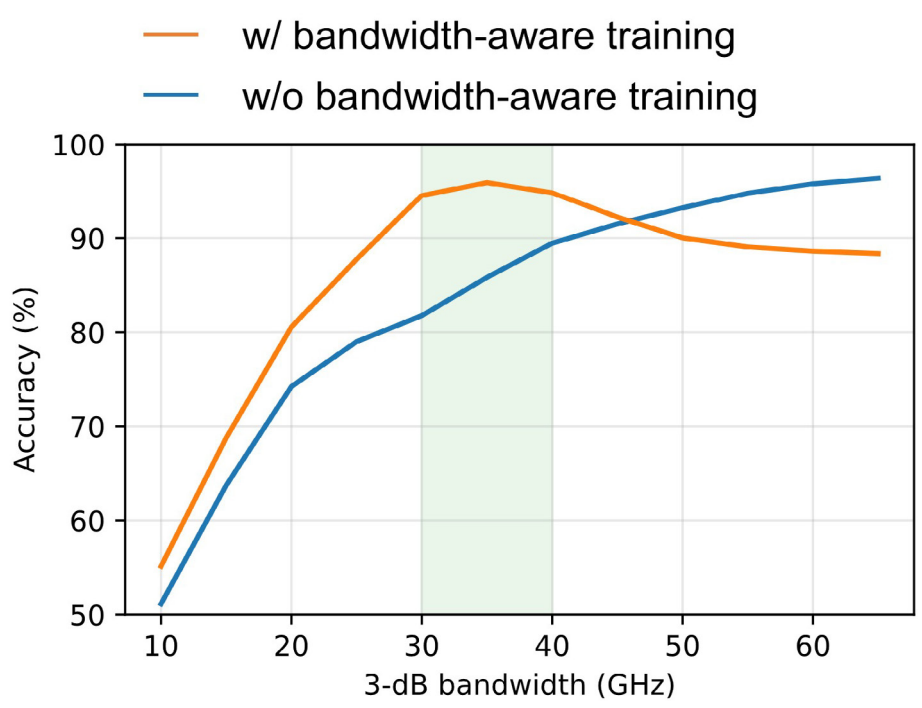

Supplementary Figure 6 Classification accuracy of NAD with and without bandwidth-aware training

Noise injection[3–5] is also applied during training to enhance the robustness of the PNN against noise in a high-speed analog computing system. In addition, sharpness-aware training[6] (SAT) is employed to further improve robustness against fabrication variations and thermal crosstalk. The pseudocode is provided below.

Pseudocode

Input: Training set $\mathbf{x}$;
Output: Trained parameters $\mathbf{\Theta}$;
1. Initialize control parameters $\mathbf{\Theta}$, $t = 0$;
2. While $t <$ iters:
    Upsample to sample rate: $\mathbf{x}_{\mathrm{up}} = \mathrm{Upsample}(\mathbf{x})$;
    E-O response of the modulator: $\mathbf{x}_{\mathrm{o}} = \mathrm{EO}(\mathbf{x}_{\mathrm{up}})$;
    MRR and system low-pass-filter: $\mathbf{x}_{\mathrm{filter}} = \mathrm{ifft}(\mathrm{fft}(\mathbf{x}_{\mathrm{o}}) * H_{\mathrm{MRR}}(f) * H_{\mathrm{LPF}}(f))$;
    Photodetection: $\mathbf{x}_{\mathrm{PD}} = |\mathbf{x}_{\mathrm{filter}}|^2$;
    Noise injection: $\mathbf{x}_{\mathrm{noisy}} = \mathbf{x}_{\mathrm{PD}} + \mathrm{N}(0, \sigma)$;
    Downsample to baud rate: $\mathbf{x}_{\mathrm{down}} = \mathrm{Downsample}(\mathbf{x}_{\mathrm{noisy}})$;
    Digital layers: $\mathbf{y} = \mathrm{DLs}(\mathbf{x}_{\mathrm{down}})$;
    Loss calculation: $L = \mathrm{Loss}(\mathbf{y}, \mathbf{y}_{\mathrm{target}}, \mathbf{\Theta})$;
    Compute gradient: $\nabla_{\mathbf{\Theta}} L(\mathbf{\Theta})$;
    Update parameters by SAT: $\mathbf{\Theta}_{\mathrm{SAT}} = \mathbf{\Theta} + \alpha \frac{\nabla_{\mathbf{\Theta}} L(\mathbf{\Theta})}{\|\nabla_{\mathbf{\Theta}} L(\mathbf{\Theta})\|^2}$;
    Compute gradient: $\nabla_{\mathbf{\Theta}} L(\mathbf{\Theta}_{\mathrm{SAT}})$;
    Update parameters: $\mathbf{\Theta} = \mathbf{\Theta} - \eta \nabla_{\mathbf{\Theta}} L(\mathbf{\Theta}_{\mathrm{SAT}})$;
    $t = t + 1$;
End
Return $\mathbf{\Theta}$

Supplementary Note 5. Computing precision

A comparison between the experimental and ideal results for 100 samples in the NAD task is shown in Supplementary Figure 7. The normalized root-mean-square error (NRMSE) is defined as Eq. (S2):

$$\mathrm{NRMSE} = \frac{1}{\max(\hat{\mathbf{y}}) - \min(\hat{\mathbf{y}})} \sqrt{\frac{1}{N} \sum_{N} (\mathbf{y} - \hat{\mathbf{y}})^2} \tag{S2}$$

where $\mathbf{y}$ and $\hat{\mathbf{y}}$ denote the experimental and simulation computing results, respectively. From Eq. (S2), the NRMSE values for the through and drop ports are calculated to be 0.03 and 0.02, respectively. The number of bits of computing precision is defined as: $\log_2 \frac{1}{\mathrm{NRMSE}}$. Based on an NRMSE of 0.03, the computing accuracy corresponds to approximately 5 bits.

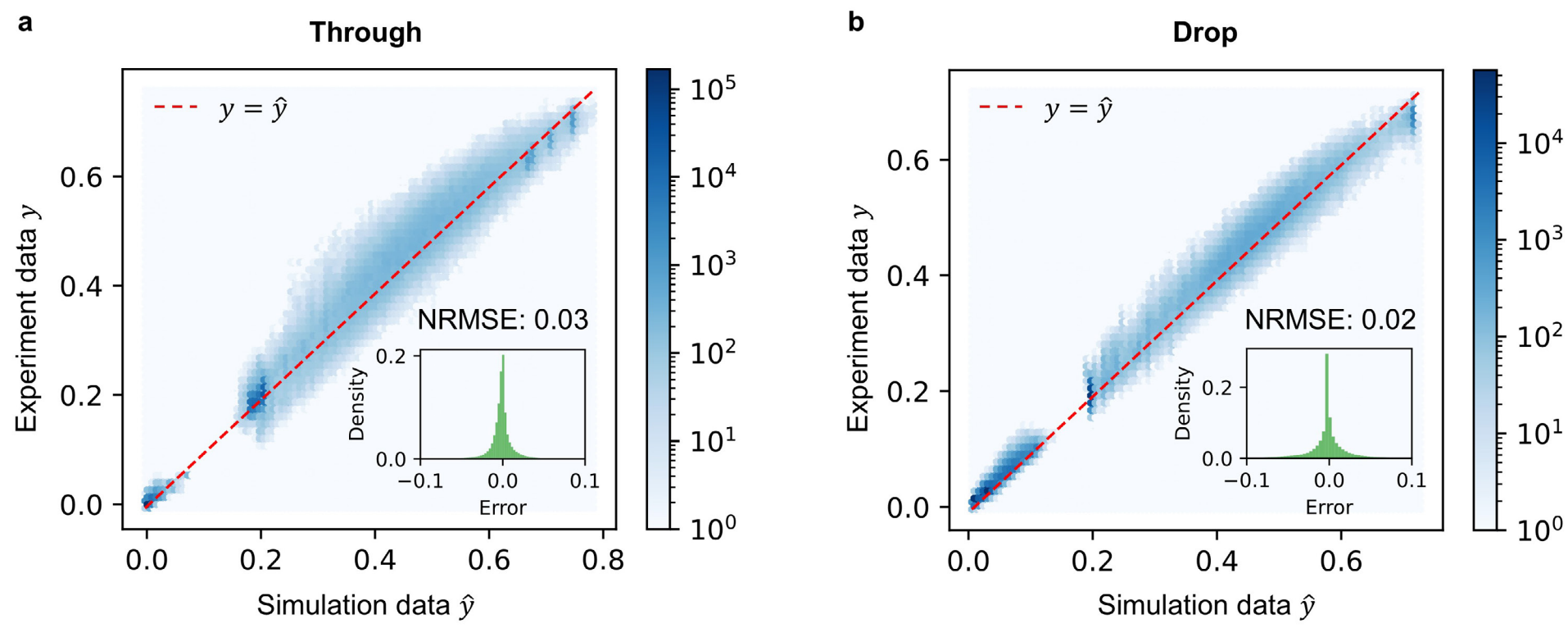

Supplementary Figure 7 Comparison of the experimental and simulation results of 100 samples in the NAD task.

Supplementary Note 6. Extend the MRR convolution engine to higher dimensions

We have demonstrated 1D and 2D convolution using the MRR convolution engine in the manuscript. The architecture can be extended to higher dimensions, as illustrated in Supplementary Figure 8. The 3D convolution can process 3D input data such as RGB images.

One dimension is mapped onto the time domain, while the other two dimensions are mapped onto the wavelength domain. For a multi-channel 3D convolution denoted as $C_{\mathrm{out}} \times C_{\mathrm{in}} \times N \times N$, where C_{out} represents the number of output channels, C_{in} represents the number of input channels, and N represents the kernel size, the conventional method requires $C_{\mathrm{out}} \times C_{\mathrm{in}} \times N \times N$ MRRs. However, our proposed method requires only $C_{\mathrm{out}} \times C_{\mathrm{in}} \times N$ MRRs.

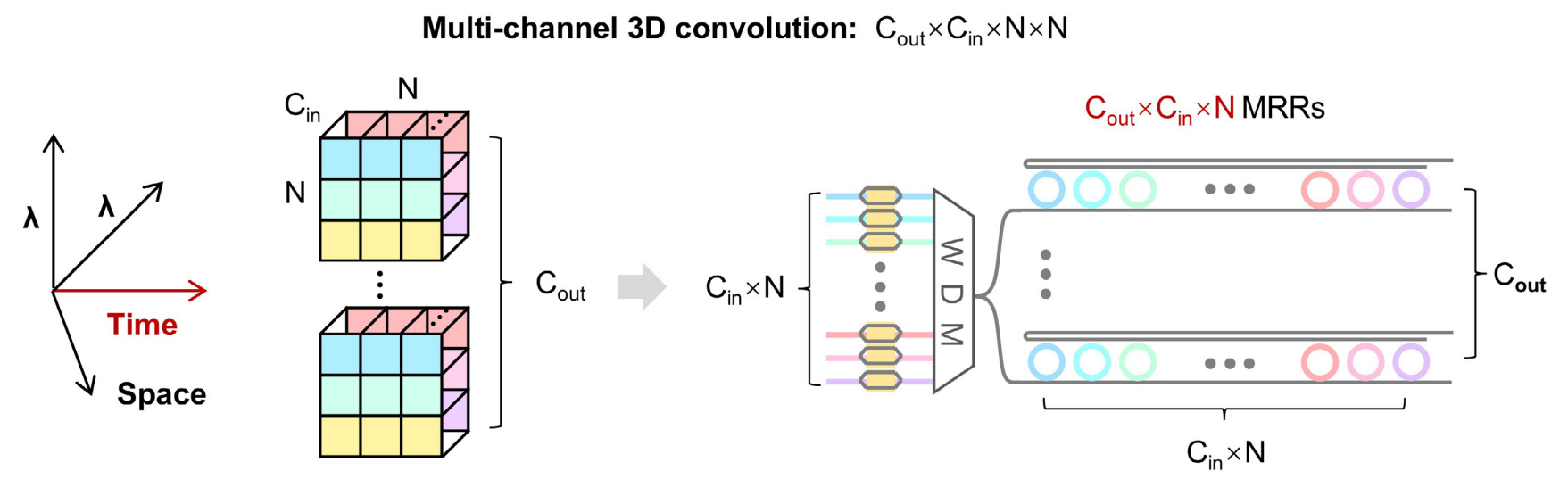

Supplementary Figure 8 Concept of multi-channel 3D convolution.

Supplementary Note 7. Single-chip integrated computing system design

In the Fashion-MNIST classification task, we define the convolution results of each channel as $y_{\mathrm{out}} = w_{\mathrm{T}} y_{\mathrm{T}} - w_{\mathrm{D}} y_{\mathrm{D}}$, where y_{T} and y_{D} are the signals from the through and drop ports, and w_{T} and w_{D} are trainable weights. This can be implemented optically by inserting a Mach–Zehnder interferometer (MZI) before the balance photodetector (BPD) to independently control the amplitudes of the through and drop ports.

Using commercially available silicon photonic fabrication platform, Mach–Zehnder modulators (MZMs), wavelength-division multiplexer (MUX), MRRs, MZIs, and BPDs can be integrated on a single chip, as illustrated in Supplementary Figure 9a. The wavelength-division multiplexer can be implemented using an arrayed waveguide grating (AWG). Alternatively, the MZMs and AWG can be replaced by cascaded microring modulators (MRMs) when using an optical frequency comb source, as shown in Supplementary Figure 9b.

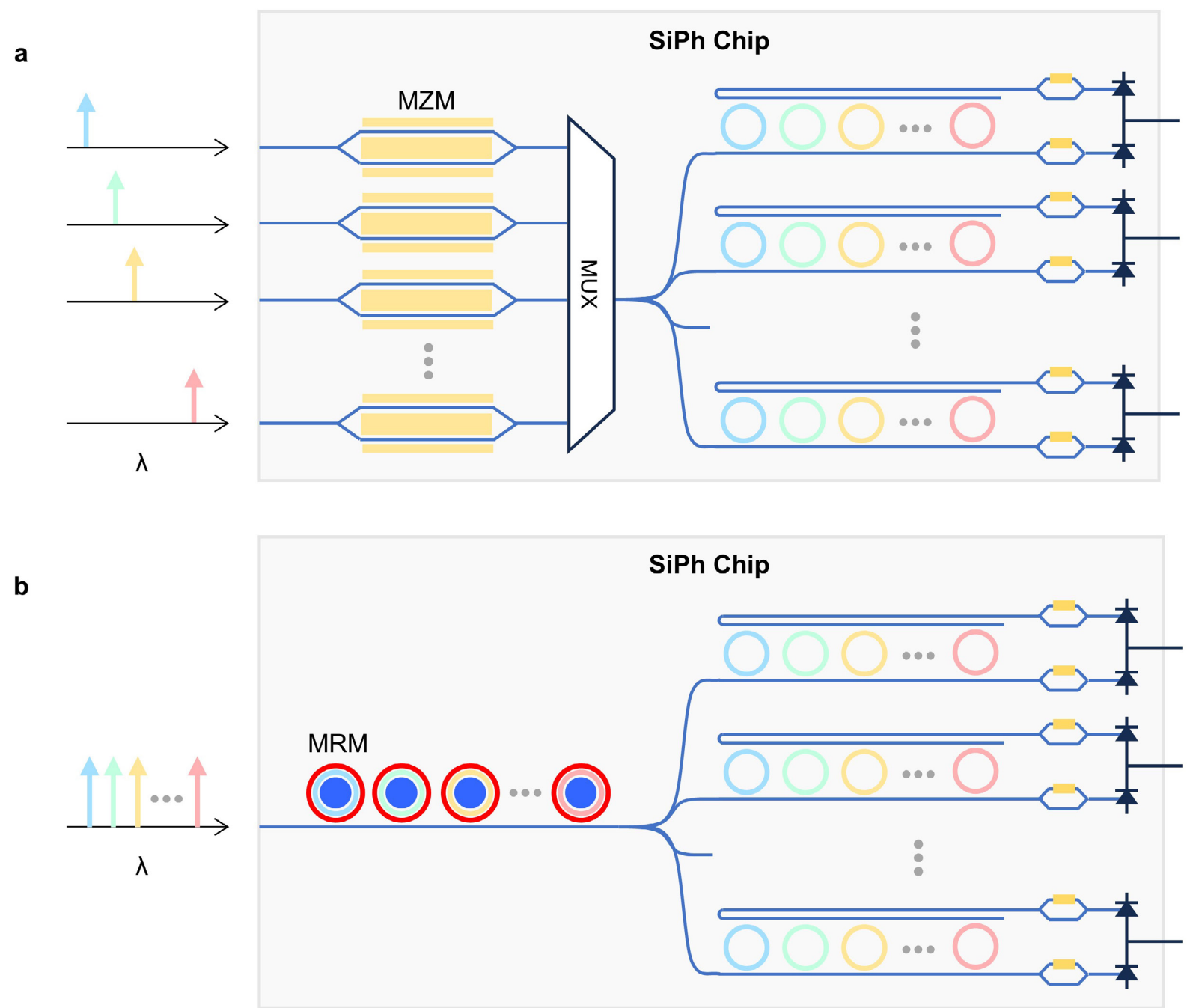

Supplementary Figure 9 Single-chip integrated computing system design. a Integration of MZMs, MUX, MRRs,

MZIs, and BPDs on a single chip. **b** Integration of MRMs, MRRs, MZIs, and BPDs on a single chip.

Supplementary Note 8. Energy efficiency estimation

Energy efficiency is defined as computing throughput divided by power consumption. We estimated the power consumption of MRRs, lasers, MZMs, PDs, DACs, and ADCs, shown in Supplementary Table 1. The total power consumption is given by Eq. (S3).

$$P_{\mathrm{total}} = n_{\mathrm{MRR}} P_{\mathrm{MRR}} + n_{\mathrm{laser}} P_{\mathrm{laser}} + n_{\mathrm{IM}} P_{\mathrm{IM}} + n_{\mathrm{PD}} P_{\mathrm{PD}} + n_{\mathrm{DAC}} P_{\mathrm{DAC}} + n_{\mathrm{ADC}} P_{\mathrm{ADC}} \quad \text{(S3)}$$

In Eq. (S3), P_{MRR} denotes the average power consumption of an MRR. The thermal tuning efficiency of the MRR is approximately 7 GHz/mW. In the photonic neural network (PNN), the maximum frequency tuning range of each MRR is set to 64 GHz, corresponding to a maximum power consumption of about 9.14 mW and an average power consumption of $P_{\mathrm{MRR}} = 4.6$ mW. The laser power consumption P_{laser} is estimated by assuming an optimized optical output power of 3 mW and a wall-plug efficiency of 0.3, yielding $P_{\mathrm{laser}} = 10$ mW. The electro-optic modulator power consumption P_{IM} is calculated for a thin-film lithium niobate (TFLN) intensity modulator driven in the quasi-linear regime with a peak-to-peak voltage of $V_{\mathrm{pp}} = 1.3$ V ($V_\pi = 2.6$ V). The resulting power consumption is $P_{\mathrm{IM}} = \frac{V_{\mathrm{pp}}^2}{2R} = 16.9$ mW, where $R = 50\,\Omega$ is the load impedance. The power consumption of the photodetector is estimated as $P_{\mathrm{PD}} = \gamma V_{\mathrm{bias}} P_{\mathrm{re}}$, where $\gamma = 0.6\ A/W$ is the responsivity, $V_{\mathrm{bias}} = 3$ V is the bias voltage, and $P_{\mathrm{re}} \approx 2$ mW is the received optical power, resulting in $P_{\mathrm{PD}} = 3.6$ mW. The power consumption of the DAC (P_{DAC}) is estimated by Ref. [7], which reports a 224-Gb/s PAM4 transmitter with an energy efficiency of 0.92 pJ/bit, corresponding to 1.84 pJ per conversion. At the 128-Gbaud symbol rate, the estimated DAC power consumption is therefore: $P_{\mathrm{DAC}} = 128 \times 10^9 \times 1.84 \times 10^{-12} \times 10^3 = 235.5$ mW. The power consumption of the ADC (P_{ADC}) is estimated by Ref. [8], which reports a 212.5-Gb/s PAM4 receiver with an energy efficiency of 1.1 pJ/bit, corresponding to 2.2 pJ per conversion. Therefore, at 128 Gbaud, the estimated ADC power consumption is: $P_{ADC} = 128 \times 10^9 \times 2.2 \times 10^{-12} \times 10^3 = 281.6$ mW.

In the Fashion-MNIST classification task, the system employs 32 MRRs, 8 lasers, 8 intensity modulators, 8 photodetectors, 8 DACs, and 8 ADCs, leading to total power consumption of $P_{\mathrm{total}} = 4528$ mW. With a computing throughput of 120.832 TOPS, the resulting energy efficiency is approximately 26.7 TOPS/W. With further optimizations, the average MRR power consumption can be reduced to $P_{\mathrm{MRR}} = 0.186$ mW using N-doped heaters[7], and the modulator driving voltage can be lowered to $V_{\mathrm{pp}} = 0.5$ V[8], corresponding to $P_{\mathrm{IM}} = 2.5$mW. With these improvements, the power consumption of the computing system with 16 × 16 MRRs operating at 128 Gbaud is projected to be 8578.8 mW. With the projected computing throughput of 1310.72 TOPS, the resulting energy efficiency is approximately 152.8 TOPS/W.

Supplementary Table 1 Power consumption estimation

	**This work**			**Projected**		
**Device**	**Power (mW)**	**Number**	**Total power (mW)**	**Power (mW)**	**Number**	**Total power (mW)**
**Laser**	10	8	80	10	16	160
**MZM**	16.9	8	135.2	2.5	16	40
**PD**	3.6	8	28.8	3.6	16	57.6
**DAC**	235.5	8	1884	235.5	16	3768
**ADC**	281.6	8	2252.8	281.6	16	4505.6
**MRR**	4.6	32	147.2	0.186	256	47.6
**Total**			**4528**			**8578.8**

Supplementary Note 9. Comparison of PNNs with digital neural networks

We compare the proposed PNNs with several digital neural network baselines, as shown in Supplementary Figure 10. For each task, we compare four different network architectures:

1. Proposed architecture (MRR-NN, blue): convolution operations are implemented by MRR temporal kernels on the PIC, while the fully connected (FC) layers remain digital.
2. FC-only digital network (FC, orange): all convolutional layers are removed, leaving only digital FC layers.
3. Parameter-matched digital CNN (PM-CNN, green): convolution operations are implemented digitally, with the same number of convolutional parameters as the MRRs.
4. Larger digital CNN (L-CNN, red): convolution operations are implemented digitally with increased model capacity; the convolutional layers contain N-times more parameters, where N is the kernel size.

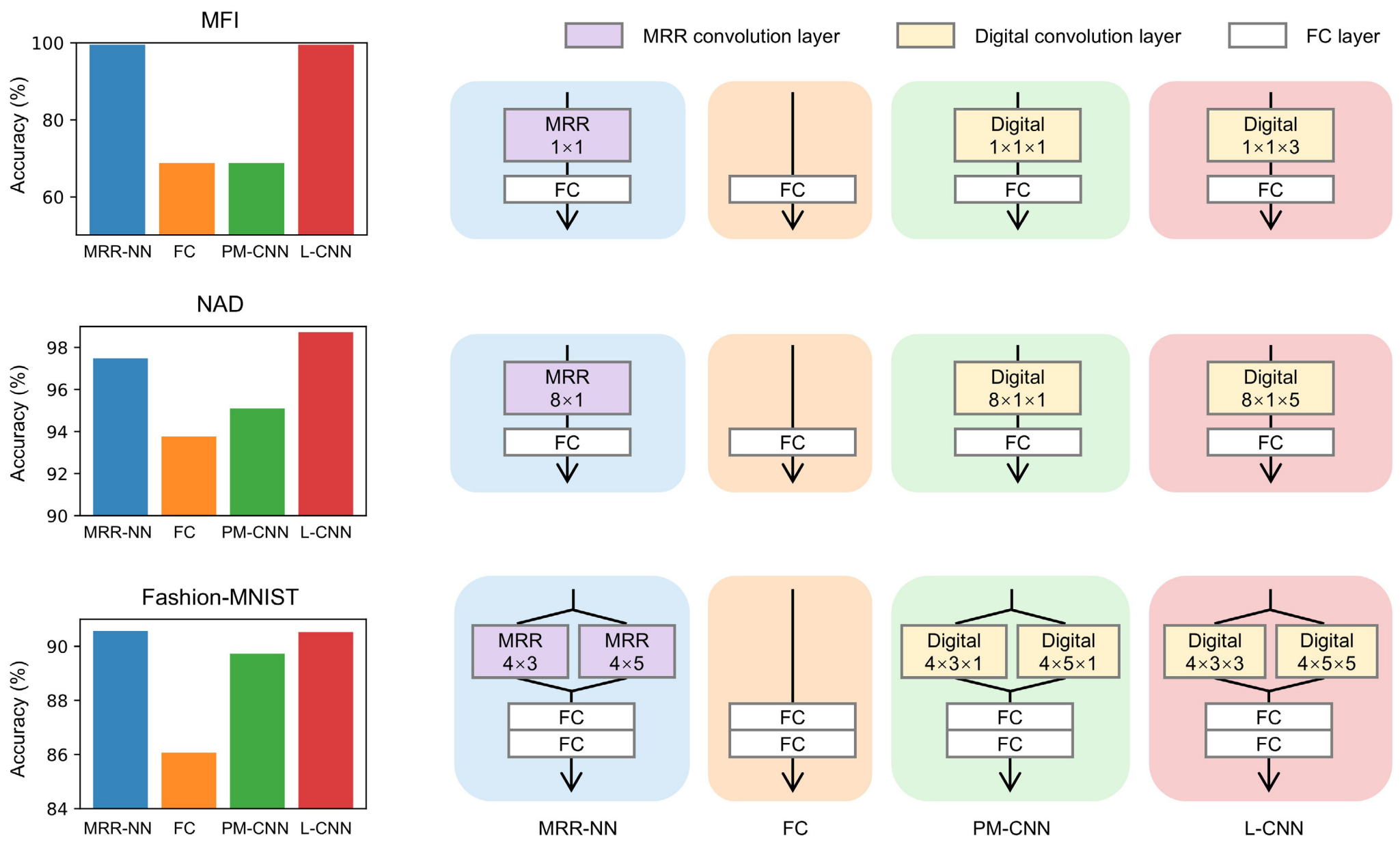

Supplementary Figure 10 Comparison of the proposed PNNs based on MRR temporal convolution kernels (MRR-NN) and several digital neural networks (FC, PM-CNN, L-CNN).

The comparison results show that the MRR-based convolution layers contribute substantially to the network performance: PNNs with photonic convolution layers (blue) consistently outperform both the FC-only networks (orange) and the digital convolution networks with the same parameter count (green) across all three tasks. Moreover, for the MFI and Fashion-MNIST classification tasks, the PNNs achieve accuracy comparable to that of significantly larger digital convolution networks (red), whose convolution layers contain N-times more parameters, where N is the kernel size. This result indicates that the MRR-based convolution layers implemented on the PIC can perform convolution operations with high parameter efficiency.

These comparisons confirm that the MRR-based temporal convolution kernel performs the core convolution operations effectively and contributes significantly to the neural network performance.

Supplementary Note 10. Data flow in PNNs versus optical communication systems

To explain why the photonic computing system can operate at 128 Gbaud, which is much higher than both the system bandwidth and MRR resonance linewidth, we clarify the fundamental difference between PNNs and optical communication systems.

In optical communication systems, the goal is to transmit each symbol with minimal distortion. Accordingly, the performance is typically evaluated by measuring the bit error rate (BER) between the transmitted and received signals. In this context, inter-symbol interference caused by insufficient channel bandwidth is regarded as an unwanted impairment that must be compensated or eliminated.

In contrast, in a neural network, the objective is not to recover the exact input symbols, but to extract task-relevant features and generate inference outputs, such as classification labels. The performance is therefore evaluated using task-level metrics, such as classification accuracy, rather than symbol-level fidelity. In this context, interactions between neighboring symbols are not necessarily detrimental. Instead, they can serve as useful computational operations. Therefore, the local symbol interactions introduced by the bandwidth-limited response can be exploited as part of the computation rather than treated solely as transmission impairments.

The data flow in the PNNs is illustrated in Supplementary Figure 11. Although the output signals of the MRRs may appear "distorted" from the perspective of optical communications, they contain task-relevant features that can improve classification accuracy.

For MFI, we experimentally use OOK and PAM4 signals at 32 Gbaud and 40 Gbaud, as shown in Supplementary Figure 11a. After passing through the MRR, the output waveforms are filtered signals that may appear "distorted". Rather than recovering the transmitted symbols, we extract amplitude histograms from these waveforms and use an FC layer to generate four output labels. The label with the maximum output value represents the classification result, namely the modulation format. Using the feature signals after the MRR convolution layer, the classification accuracy reaches 99.5%. In contrast, when using the original signals without MRR-based feature extraction, the accuracy is only 71.0%.

For NAD, the network traffic data are encoded as the waveform amplitude and transmitted at a symbol rate of 128 Gbaud, as shown in Supplementary Figure 11b. After the MRR convolution layer, the output waveforms are 128-Giga symbol/s feature signals that differ from the input waveforms and may appear distorted. An FC layer then takes these feature waveforms as input and generates 20 output labels. The label with the maximum output value represents the classification result. Using the feature waveforms after the MRR convolution layer, the classification accuracy reaches 96.4%. In contrast, using the original network traffic data directly gives an accuracy of 93.7%.

For the Fashion-MNIST classification task, the reshaped image pixel values are encoded as the waveform amplitude and transmitted at a rate of 128-Giga pixel/s, as shown in Supplementary Figure 11c. The output 128-Giga pixel/s signals can be reshaped into two-dimensional feature images, which differ from the original images and may appear distorted. The FC layers then take these feature images as input and generate 10 output labels. The label with the maximum output value represents the classification result. Using the feature images after the MRR convolution layer, the classification accuracy reaches 90.6%. In contrast, using the original images directly gives an accuracy of only 86.1%.

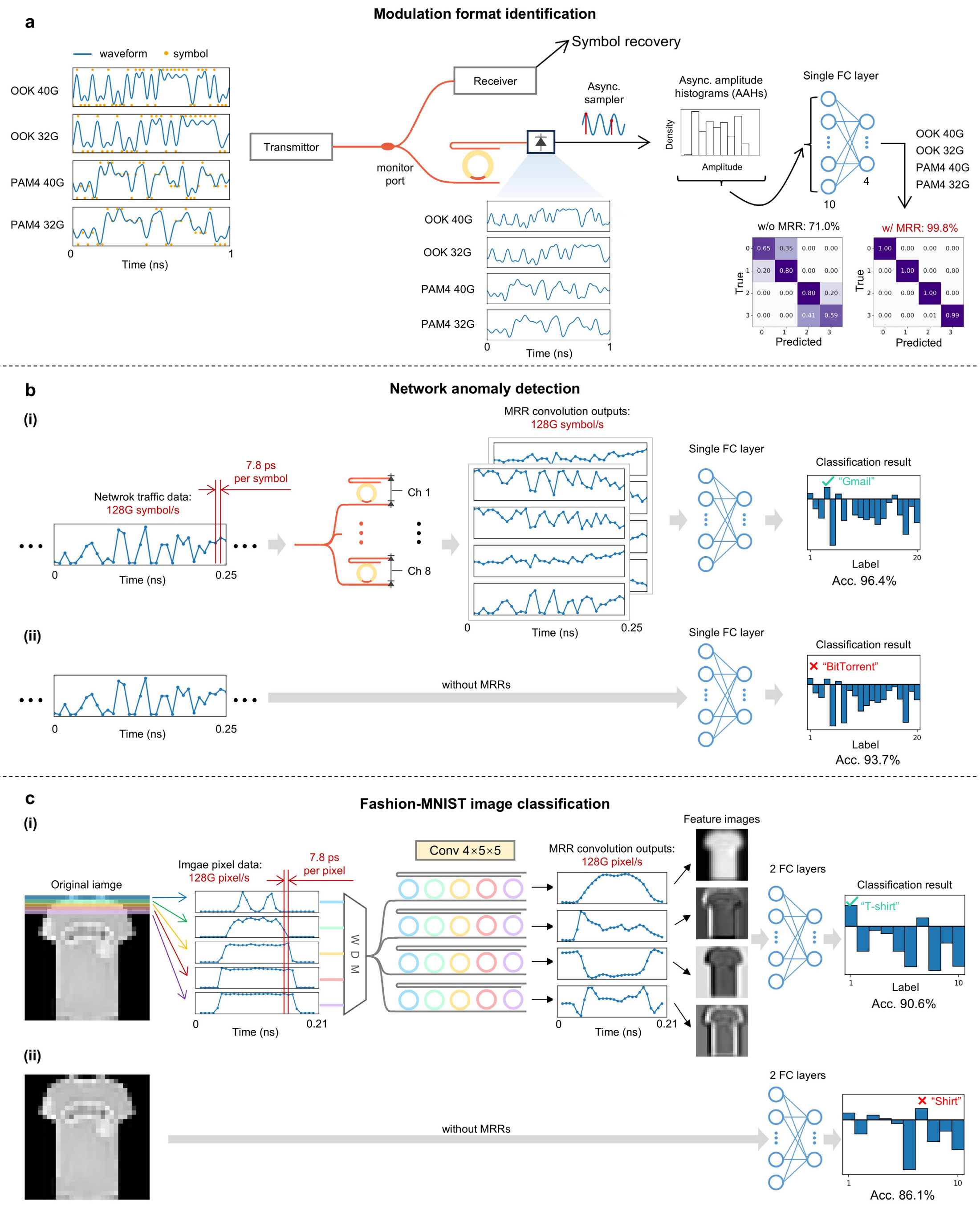

Supplementary Figure 11 Data flow in PNNs.

Supplementary Note 11. High-speed advantage for the temporally uncorrelated task

In the proposed MRR-based convolution, higher data rates lead to larger effective kernel size, thereby enlarging the receptive field of the convolution operation and enabling the network to extract broader contextual features. This advantage is not limited to temporal sequence tasks such as NAD. We further show that an MRR-based PNN achieves higher accuracy at higher symbol rates in an MNIST[9] image classification task.

The setting is shown in Supplementary Figure 12a. Each image is reshaped into a 1 × 784 one-dimensional sequence, followed by an MRR convolution layer for feature extraction and a fully

connected layer for classification. The relationship between classification accuracy and symbol rate is shown in Supplementary Figure 12b. The accuracy increases from 91.6% to 94.3% as the symbol rate increases from 1 Gbaud to 256 Gbaud, and then slightly decreases to 94.1% at 1024 Gbaud. These results indicate that the benefit of larger effective kernel sizes is not limited to explicitly temporal datasets such as network traffic signals but can also improve feature extraction for data with broader contextual structures.

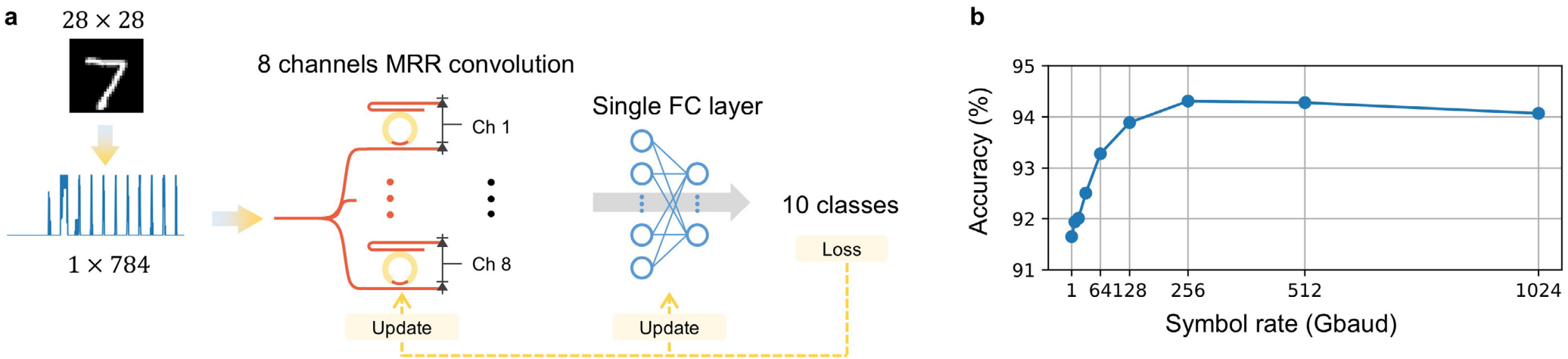

Supplementary Figure 12 High-speed advantage for the temporally uncorrelated task. a PNNs based on MRR temporal kernels for MNIST classification**. b** Classification accuracy with symbol rate.

Supplementary Note 12. Fabrication variations characterization

To characterize the fabrication variations of the MRR chip, we measured the through port transmission spectra of the 8×8 MRR array, as shown in Supplementary Figure 13a. We then extracted the statistical distributions of the measured FWHM and extinction ratio, as shown in Supplementary Figure 13b. In addition, we fit the parameters of self-coupling efficiency (t_1, t_2) and round-trip attenuation factor (a_{rt}) in Eq. (1) of the manuscript according to the spectra of 64 MRRs. Here we assume that $t = t_1 = t_2$, because both gaps between the MRR and through/drop bus waveguides are designed as 100 nm. The statistical distributions of the fitted parameters are shown in Supplementary Figure 13c.

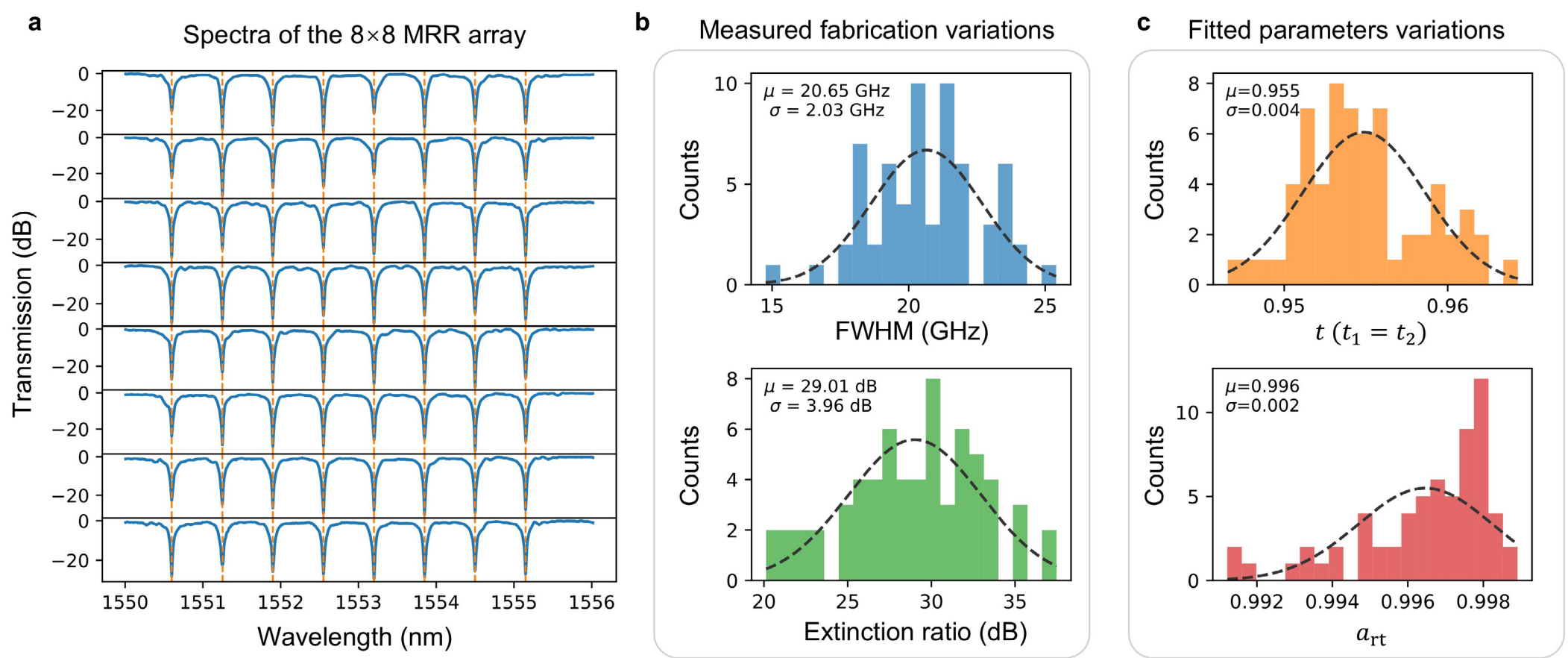

Supplementary Figure 13 Fabrication variation characterization. a Spectra of the 8×8 MRR array **b** statistical distributions of the measured FWHM and extinction ratio **c** Fitted parameters of self-coupling efficiency $(\boldsymbol{t_1}, \boldsymbol{t_2})$ and round-trip attenuation factor $(\boldsymbol{a}_{\mathbf{rt}})$

Supplementary Note 13. Thermal crosstalk characterization

We experimentally characterize the thermal crosstalk effect of the 8×8 MRR array. The results are shown in Supplementary Figure 14. We define the thermal crosstalk coefficient $(c_{i,j})$ between MRR $[m, n]$ and MRR $[p, q]$ as the ratio between the resonance wavelength shift induced in MRR $[m, n]$ and MRR $[p, q]$ when electrical power is applied only to MRR $[p, q]$, where $i = | m - p |$ and $j = | n - q |$ represent the row and column distances between two

MRRs.

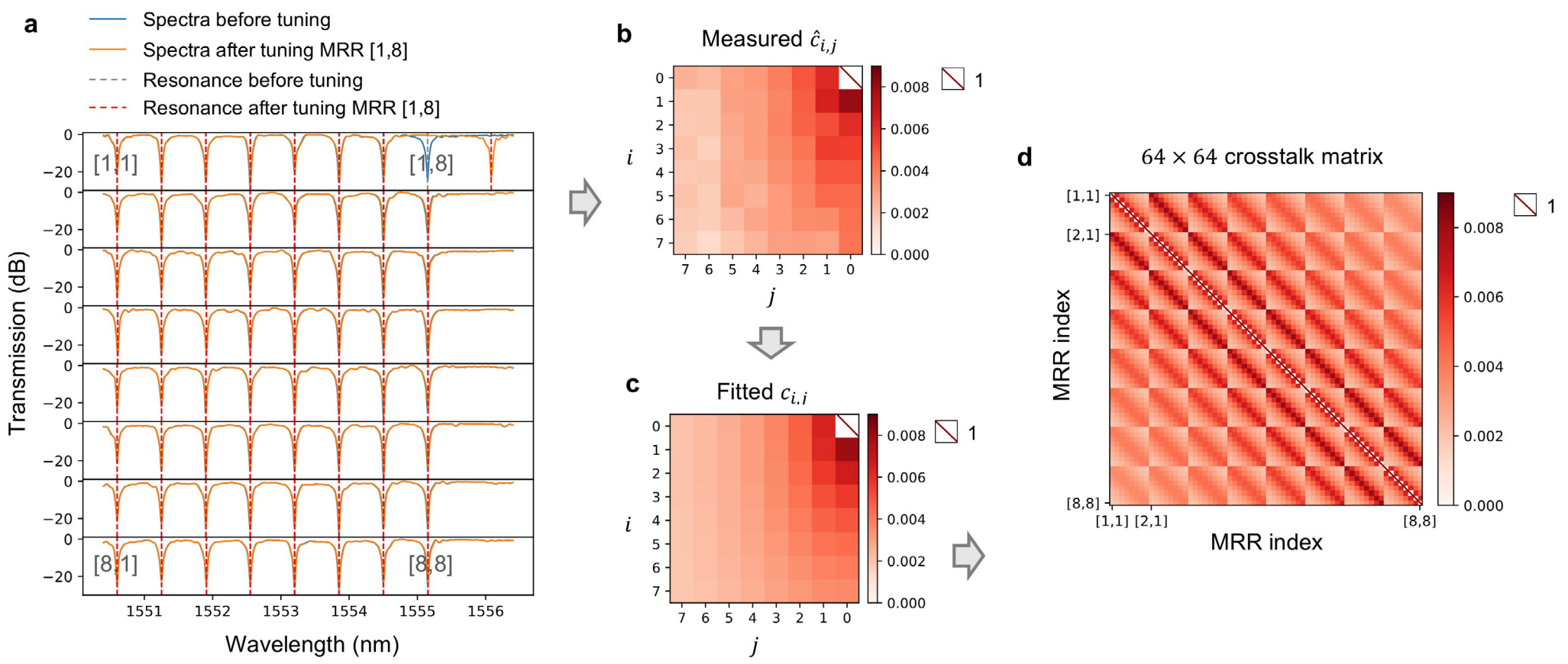

Supplementary Figure 14 Thermal crosstalk of the $\mathbf{8 \times 8}$ MRR array. a. Spectra of the $\mathbf{8 \times 8}$ MRR array before and after thermally tuning MRR [1,8]. **b.** Experimentally measured thermal crosstalk coefficient $\hat{\boldsymbol{c}}_{\boldsymbol{i,j}}$. **c.** Fitted thermal crosstalk coefficient $\boldsymbol{c}_{\boldsymbol{i,j}}$. **d.** Thermal crosstalk matrix of the $\mathbf{8 \times 8}$ MRR array

The procedure of characterizing $c_{i,j}$ and thermal crosstalk matrix $\mathbf{M}$ is as follows:

1. A picked MRR (MRR [1,8] in our experiment) in the 8×8 array is thermally tuned, and the transmission spectra before and after tuning are measured, as shown in Supplementary Figure 14a. The gray and red dashed lines indicate the resonance positions before and after thermal tuning, respectively.
2. The experimentally measured $\hat{c}_{i,j}$ values are calculated for $i \in [0,7]$ and $j \in [0,7]$, as shown in Supplementary Figure 14b.
3. Generally, $c_{i,j}$ decreases with row (i) and column (j) distance due to heat conduction. To eliminate the effect of experimental measurement error, a mathematical model is used to fit $\hat{c}_{i,j}$ while ensuring the monotonic-decrease property, as described in Eq. (S4) and shown in Supplementary Figure 14c. The asymmetric coefficients associated with i and j originate from the asymmetric physical spacing and deep-trench structures along the row and column directions of the fabricated chip.

$$c_{i,j} = \begin{cases} \dfrac{1}{14.60\sqrt{3.06i^2 + 15.12j^2 + 99.55}}, & (i,j) \neq (0,0) \\ 1, & (i,j) = (0,0) \end{cases} \tag{S4}$$

4. Based on Eq. (S4), the 64×64 thermal crosstalk matrix of the 8×8 MRR array is constructed, as shown in Supplementary Figure 14d. The element of the matrix is the thermal crosstalk coefficient between any two MRRs.

Supplementary Note 14. Programming of MRR array

Accurate programming of large-scale MRR arrays is challenged by fabrication non-uniformity, thermal crosstalk, and environmental fluctuations. To address these challenges, we employ the chip-in-the-loop[10] (ChiL) approach for on-chip configuration, as shown in Supplementary Figure 15a. This method enables efficient and accurate programming of large-scale MRR arrays to the desired detuning positions while inherently accounting for fabrication imperfections and thermal crosstalk effects without characterizing them.

ChiL is an interactive optimization method without using conventional look-up tables or modeling the thermal crosstalk matrix coefficients. The procedure of each iteration is as follows:

1. Measure the resonance wavelengths λ_{exp} of all MRRs.

2. Compare the λ_{exp} with the target resonance wavelength λ and calculate loss.
3. Calculate the gradient by Eq. (S5) - (S6):

$$\frac{\widetilde{\partial L}}{\partial \mathbf{p}} = (\boldsymbol{\lambda}_{\text{exp}} - \boldsymbol{\lambda}) \odot \frac{1}{C(\mathbf{f}')} \tag{S5}$$

$$\mathbf{f}' = \frac{\boldsymbol{\lambda}_{\text{exp},k} - \boldsymbol{\lambda}_{\text{exp},k-1}}{\mathbf{p}_k - \mathbf{p}_{k-1}} \tag{S6}$$

where p is the squares of currents applied to the thermal phase shifters, f' is the estimated gradient from the previous iteration, $C(f')$ represents the clipping function to constrain the gradient range.

4. Update the currents according to Eq. (S7):

$$\mathbf{p} = \mathbf{p} - \eta \frac{\widetilde{\partial L}}{\partial \mathbf{p}} \tag{S7}$$

where η is the learning rate.

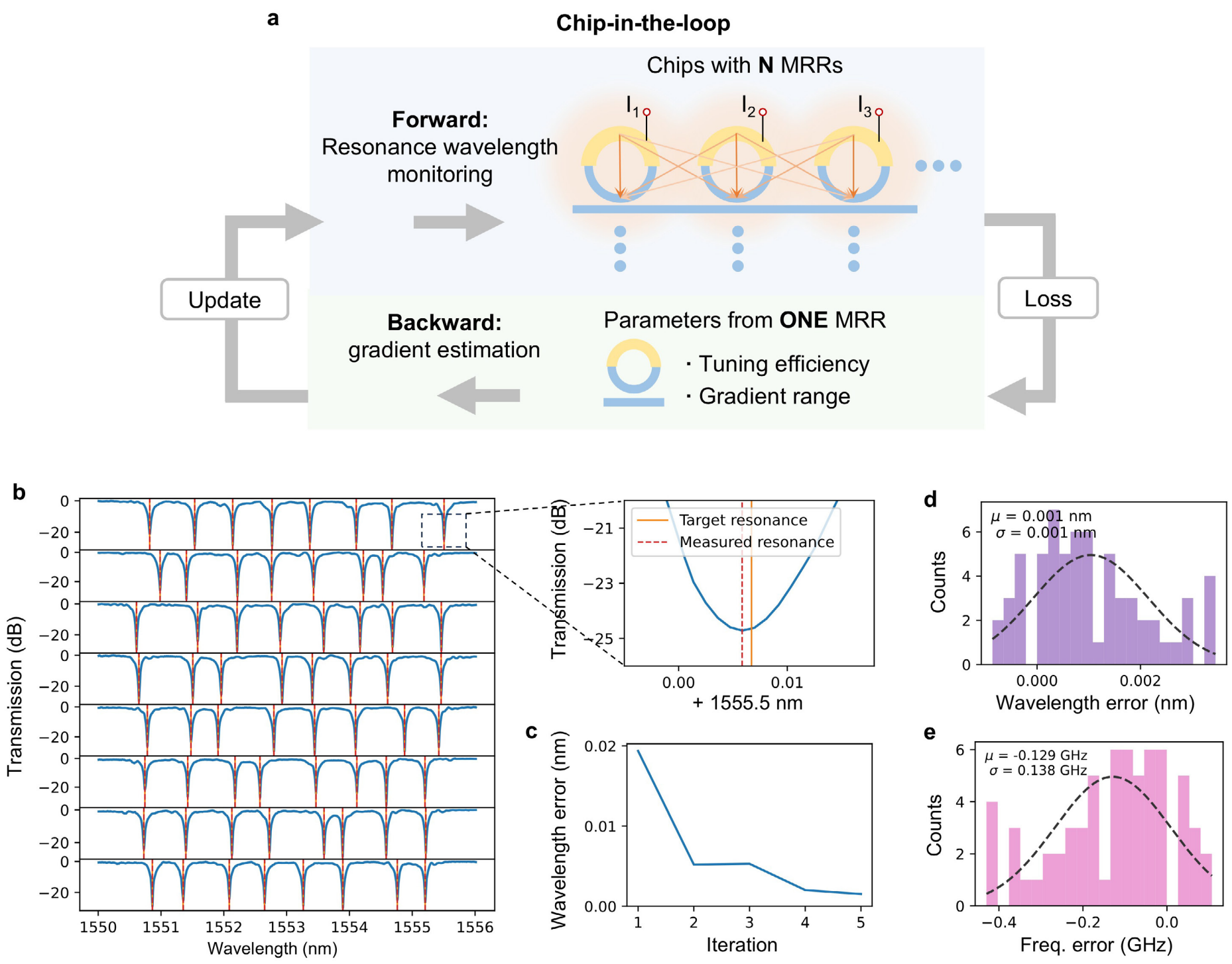

Supplementary Figure 15 Programming of MRR array. **a** Concept of Programming the MRR array using ChiL. **b** Spectra of the **8 × 8** MRR array when tuned to randomly generated target resonance wavelengths. **c** Average absolute value of wavelength error during iterations. **d** Statistical distribution of wavelength detuning error. **e** Statistical distribution of frequency detuning error.

To evaluate the programming accuracy, we program the 8×8 MRR array with randomly generated target resonance wavelengths, as shown in Supplementary Figure 15b. The orange solid lines represent the target resonance wavelengths, and the red dashed lines represent the measured resonance wavelengths in the experiment. The average resonance tuning error is only about 0.001 nm, as shown in Supplementary Figure 15d. Importantly, our method requires only five iterations to obtain this programming accuracy, as shown in Supplementary Figure 15c. The total configuration time can be less than 20 ms using a field-programmable gate array (FPGA)[11]. After that, the applied currents are fixed during inference. Therefore, the training or

optimization time is not a limiting factor in high-speed inference applications. Finally, we calculate the frequency detuning error by $\mathrm{Error}_{\mathrm{freq}} = \frac{c}{\lambda_{\mathrm{exp}}} - \frac{c}{\lambda}$, where c is the speed of light. The standard deviation of frequency detuning error is about 0.138 GHz, as shown in Supplementary Figure 15e.

Supplementary Note 15. Robustness against fabrication variations and tuning error

Though we can program the MRRs to the target resonance positions with small tuning error, fabrication variations and residual tuning errors may induce discrepancies between the actual MRR impulse response and the digital MRR model. To address this problem, we use sharpness-aware training to improve the robustness of PNNs against these imperfections.

To evaluate the robustness against fabrication variations, we introduce Gaussian variations of the self-coupling coefficients (t_1, t_2) and the round-trip attenuation factor (a_{rt}) with different standard deviations (σ) in the testing model. The resulting accuracy is shown in Supplementary Figure 16a. The accuracy of all three tasks demonstrated in the manuscript remains largely unchanged even when scaling them by 2×, showing that the trained PNNs are robust against fabrication variations and can be reliably transferred across different hardware instances.

To evaluate the robustness against tuning error, we introduce different frequency tuning offsets in the testing model. The resulting accuracy is shown in Supplementary Figure 16b. The accuracy of all three tasks demonstrated in the manuscript remains largely unchanged within ±1 GHz resonance frequency offset, which is more than 2× the maximum offset in the experiment. This result indicates that the trained model is robust against tuning errors.

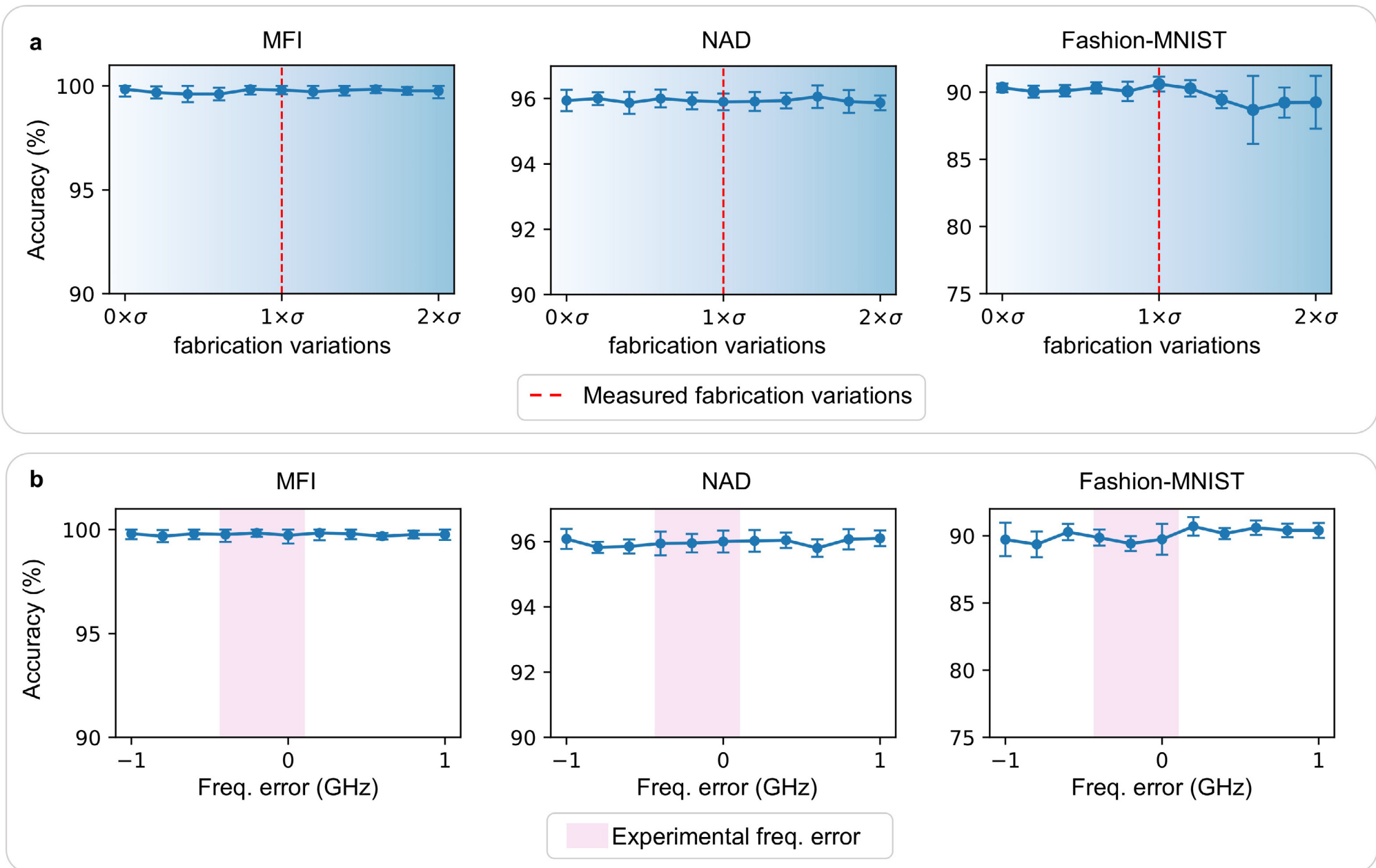

Supplementary Figure 16 Robustness against fabrication variations and tuning error. a Classification accuracy vs. fabrication variations. **b** Classification accuracy vs. frequency tuning error. Error bars indicate the standard deviations.

Supplementary Note 16. Noise and computing precision tolerance

We use noise injection during training to increase the tolerance of PNNs against noise and

distortion in the high-speed computing system. To evaluate the tolerance against noise, we introduce Gaussian noise in the testing model. The accuracy with different signal-to-noise ratios (SNRs) and corresponding computing precision (defined in Supplementary Note 5) is shown in Supplementary Figure 17. Here, the SNR is defined as: $\text{SNR (dB)} = 10\log_{10}\frac{<\hat{y}^2>}{<(y-\hat{y})^2>}$, where $\hat{y}$ and y represent the simulated and experimental noisy computing results, $<\cdot>$ denotes average operation.

For the MFI task, the classification accuracy remains above 99% at SNR of 20 dB and computing precision of about 4.4 bits. For the NAD task, the accuracy remains above 95% at SNR of 14 dB and computing precision of about 4.4 bits. For the Fashion-MNIST classification task, the accuracy remains above 89% at SNR of 13 dB and computing precision of about 4.2 bits. These results indicate that the proposed PNN can maintain stable inference performance under practical noisy operating conditions.

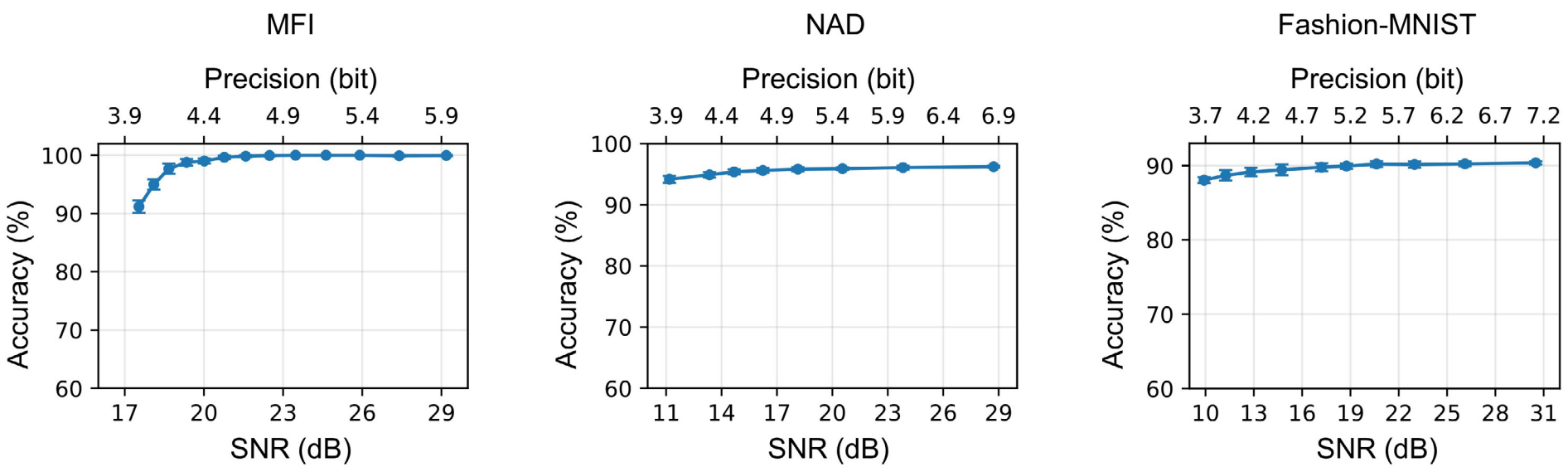

Supplementary Figure 17 Classification accuracy vs. SNR and computing precision. Error bars indicate the standard deviations.

Supplementary Note 17. Timing error tolerance

We conduct simulations to test the classification accuracy with different timing errors in the high-speed computing system, as shown in Supplementary Figure 18.

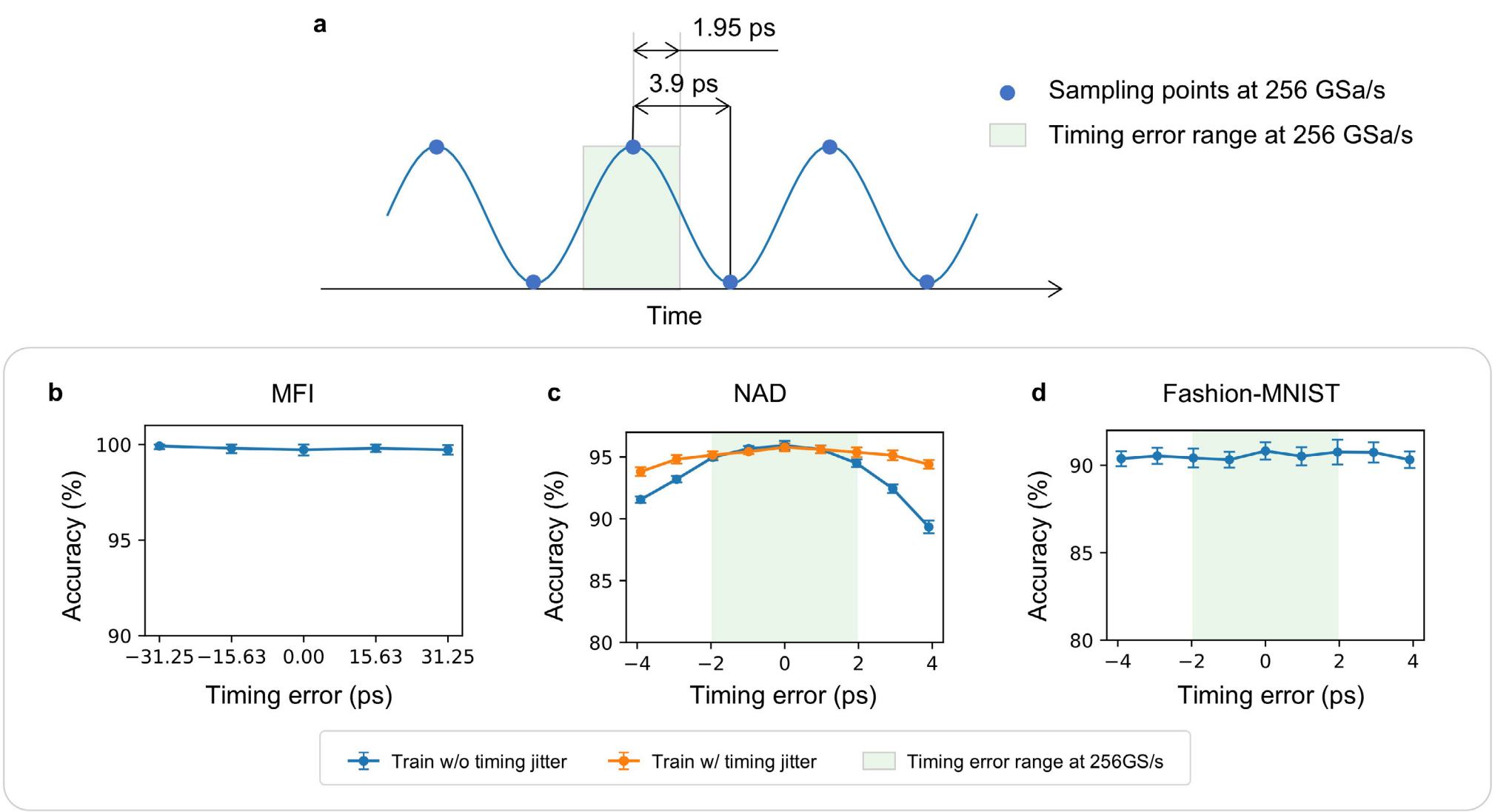

Supplementary Figure 18 Classification accuracy vs. timing error. Error bars indicate the standard deviations.

For the MFI task, we use asynchronous amplitude histograms (AAHs) as classification features. Since AAHs represent the statistical distribution of waveform amplitudes rather than precise temporal samples, they are inherently insensitive to timing errors. As shown in Supplementary

Figure 18b, the classification accuracy remains nearly unchanged over a timing error range of ±1 symbol period for the target 32 Gbaud signal.

For the NAD and Fashion-MNIST tasks, the symbol rate reaches 128 Gbaud. The experimental sampling rate is 256 GSa/s, corresponding to a sampling interval of 3.91 ps. Therefore, the maximum temporal mismatch between an ideal sampling instant and the nearest acquired sample is ±1.95 ps (half a sampling interval), as shown in Supplementary Figure 18a. For Fashion-MNIST classification, the accuracy is largely unchanged within twice the maximum timing error, as shown in Supplementary Figure 18d. For the NAD task, the accuracy remains above 94% within the maximum timing error range and drops below 90% at twice the maximum timing error. After incorporating timing jitter during training, the PNN becomes significantly more robust against its effect. As shown in Supplementary Figure 18c, the accuracy remains above 95% within the maximum timing error range and over 94% even at twice the maximum timing error.

Supplementary Note 18. Long-term operation accuracy and stability

To evaluate long-term operational accuracy and stability, we continuously performed the NAD task over a period of 5 hours. The classification accuracy remained stable at approximately 95% throughout the experiment, without any noticeable performance degradation, as shown in Supplementary Figure 19

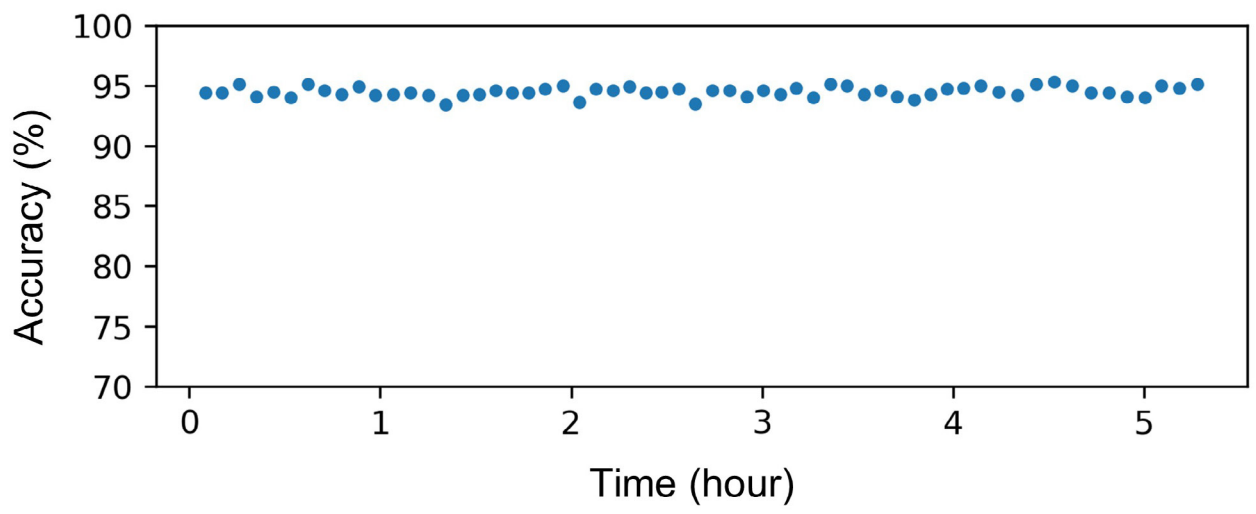

Supplementary Figure 19 Long-term accuracy of the NAD task in experiment

Supplementary Note 19. MRRs for image classification without FC layers

The proposed MRR temporal convolution can itself directly accomplish certain tasks without any additional FC layers. To clarify this point, we provide a proof-of-concept fully photonic classification example.

As shown in Supplementary Figure 20, a 4 × 3 MRR array is used to classify four 3 × 3 binary images corresponding to the letters "C", "U", "H", and "K". In this scheme, the pixel values along the vertical dimension are encoded onto 3 different optical wavelengths, while the horizontal dimension is encoded temporally. The 3 × 3 image requires a temporal effective kernel size of 3, therefore the data rate is set as 64 Gbaud according to the impulse response of the MRR on our chip. The MRR array functions as a 4 × 3 × 3 temporal convolution kernel and generates 4 outputs, where each output channel corresponds to one target class.

After training, the classification result can be directly obtained from the photocurrents of the four photodetectors, without requiring any digital FC layers or digital post-processing. The detector corresponding to the correct class produces the highest response for each input image, demonstrating that the MRR-based temporal convolution alone can perform complete classification functionality on-chip.

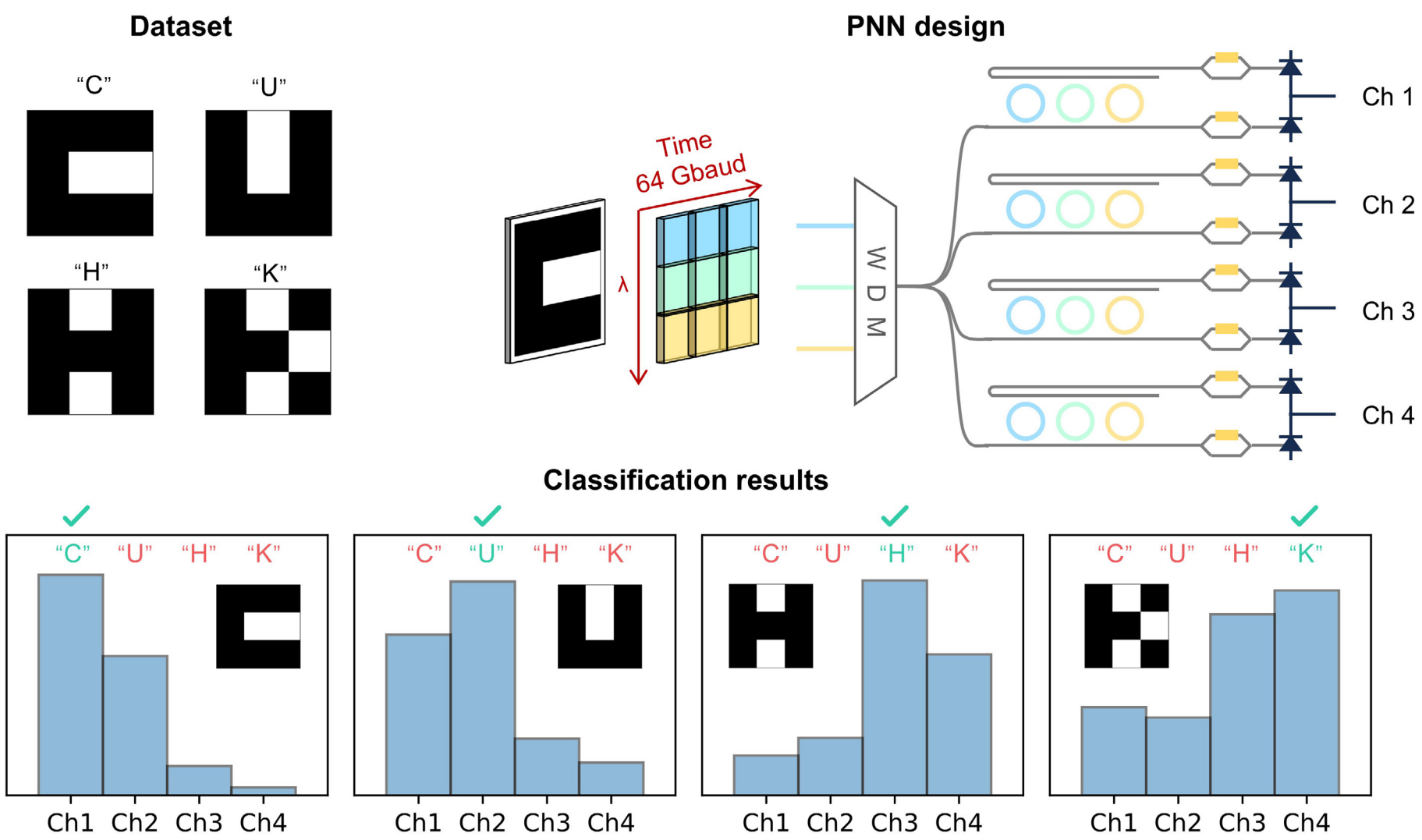

Supplementary Figure 20 An MRR temporal kernel array for image classification without any additional digital layers.

Supplementary Note 20. MRRs for Optical communication signal equalization

The MRR temporal kernel can be used for optical fiber communication signal equalization. Here we demonstrate the equalization of 120 Gbaud PAM4 signals after 80-km C-band single-mode optical fiber transmission in the intensity-modulation direct-detection (IMDD) system. The simulation setting is shown in Supplementary Figure 21a. The optical fiber has a chromatic-dispersion coefficient of $D = 17$ ps/nm/km and a nonlinear coefficient of $\gamma = 1.3\ \mathrm{W^{-1}km^{-1}}$. During transmission, the signal experiences chromatic dispersion and nonlinear impairments of self-phase modulation, which introduce severe temporal distortion and inter-symbol interference, making temporal feature extraction essential for signal recovery. The results in Supplementary Figure 21b show that without equalization, the bit error rate (BER) is 4.63×10^{-1}. Using a conventional 1500-tap feed-forward equalizer (FFE), the BER is reduced to 9.50×10^{-2}, which is significantly higher than the hard-decision forward-error-correction (HD-FEC) threshold of 3.8×10^{-3}. A nonlinear digital convolutional neural network (CNN) provides a similar BER of 9.21×10^{-2}. In contrast, by using two MRR temporal convolution kernels before the photodetectors, the BER is reduced below the HD-FEC threshold to 8.09×10^{-4} with only 2×161-tap FFEs, thereby meeting the HD-FEC threshold. The eye diagrams before and after equalization are shown in Supplementary Figure 21d. Without equalization, the eye diagram is nearly closed due to ISI, whereas MRR equalization significantly opens the eye, indicating substantially improved signal quality. In addition, the proposed MRR-based equalizer requires significantly fewer digital parameters compared with conventional FFE and digital CNN approaches, as shown in Supplementary Figure 21c. These results demonstrate that the proposed temporal convolution architecture can handle high-order nonlinear impairment compensation tasks, outperforming conventional digital signal processing methods.

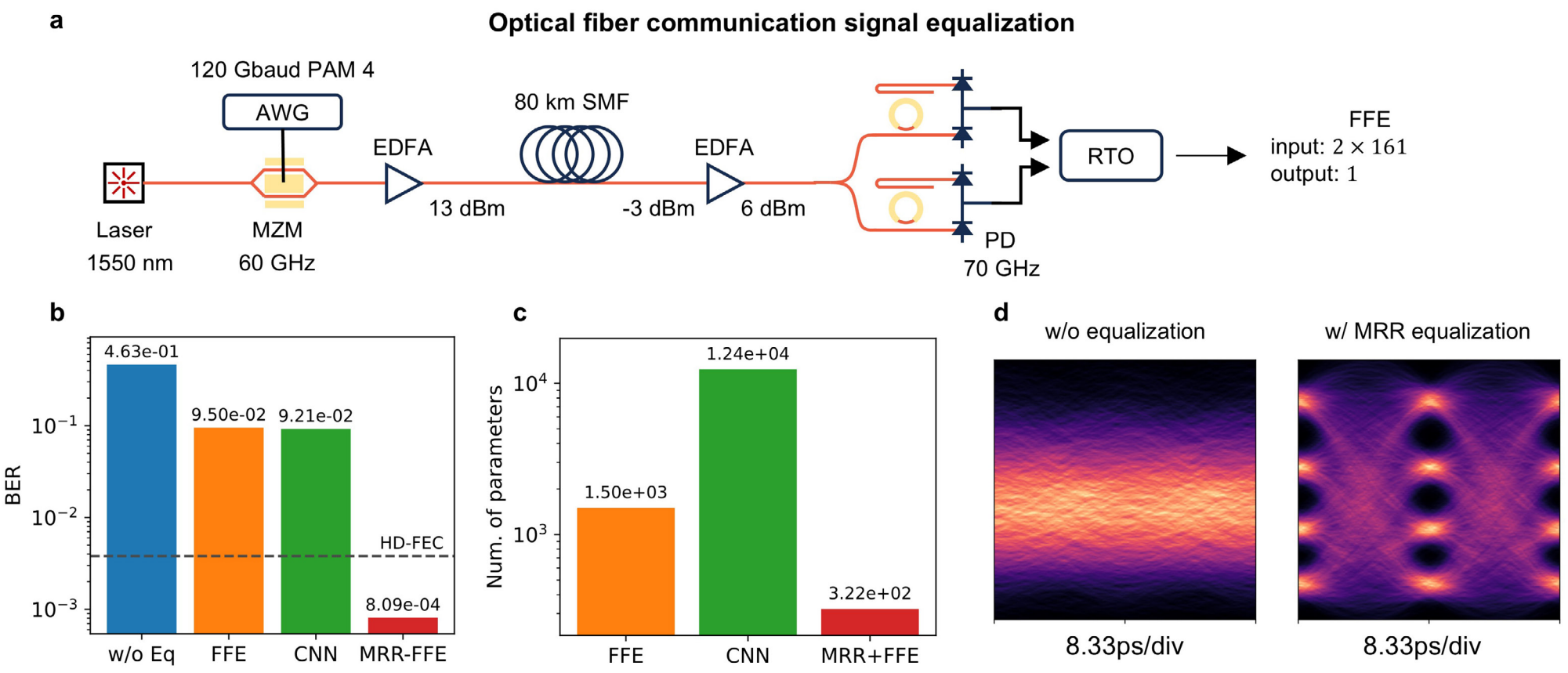

Supplementary Figure 21 MRR temporal convolution kernel for optical fiber communication signal equalization

Supplementary Note 21. MRRs for Nonlinear time-series prediction

The MRR temporal kernel can be used for nonlinear time-series prediction. Here we demonstrate the prediction of Santa Fe Laser data, which is generated from a chaotic system with unknown dynamics and widely used for nonlinear sequence prediction[12]. The simulation setting is shown in Supplementary Figure 22a. We use the data from t-7 step to t step to predict the data of t+1 step. The neural network consists of an 8-channel MRR temporal convolution layer for feature extraction and a FC layer for prediction. The simulation results are shown in Supplementary Figure 22b. When operating at 128 Gbaud, each MRR functions as a temporal convolution kernel for extracting time-domain features from the sequence, and the proposed MRR-based convolutional neural network achieves a normalized mean-square error (NMSE) of 0.012. In contrast, when the symbol rate is 1 Gbaud, MRRs work as scalar weights, resulting in NMSE of 0.069, which is close to the NMSE (0.079) of using the FC layer only. These results indicate that the proposed temporal convolution architecture provides substantially stronger temporal feature extraction capability than conventional weighting-based photonic processors.

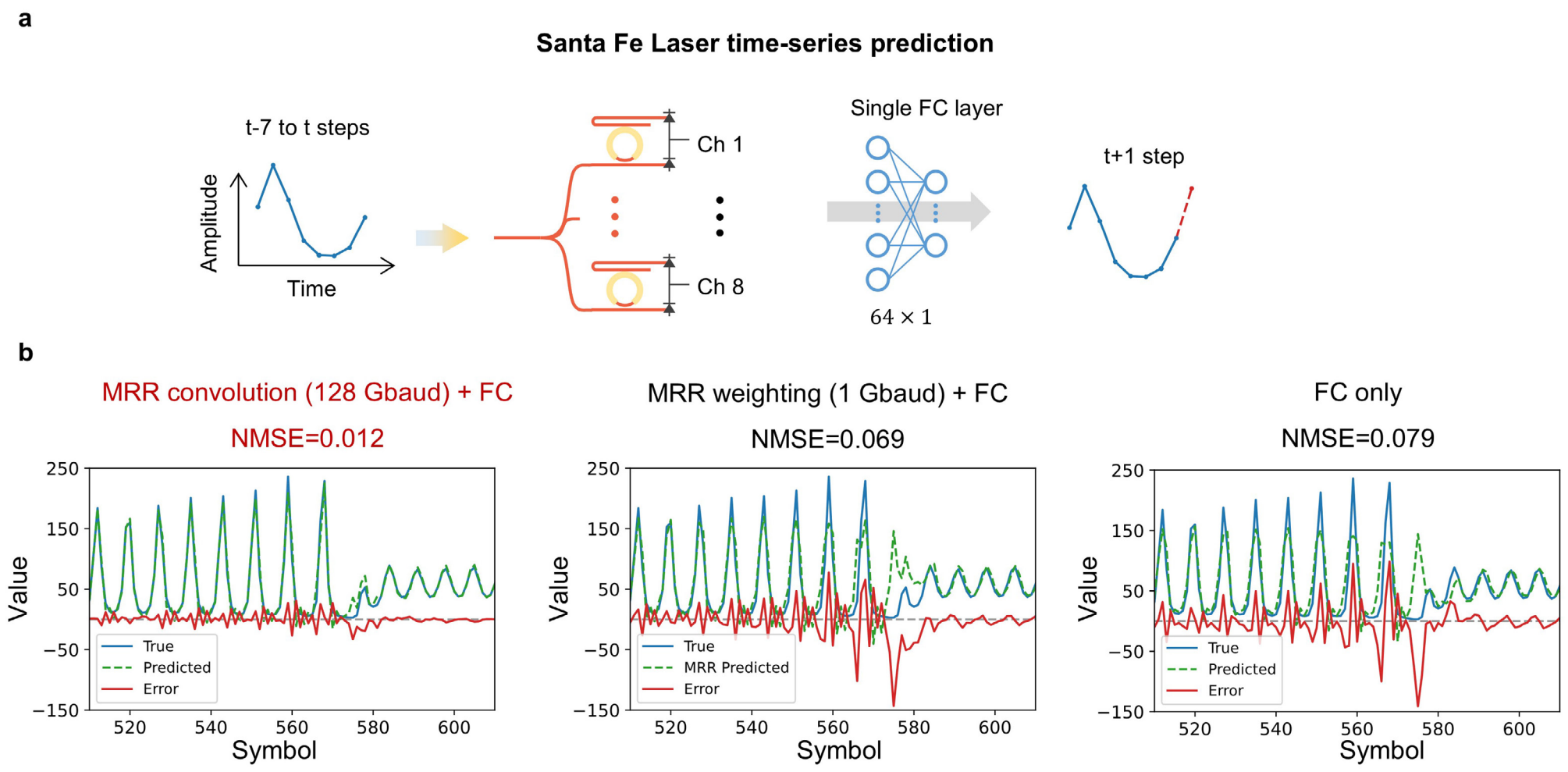

Supplementary Figure 22 MRR temporal convolution kernel for Santa Fe Laser nonlinear time-series prediction

Reference

1. Paszke, A. *et al.* PyTorch: An Imperative Style, High-Performance Deep Learning Library. in *Advances in Neural Information Processing Systems* vol. 32 (Curran Associates, Inc., 2019).
2. Moralis-Pegios, M. *et al.* Neuromorphic Silicon Photonics and Hardware-Aware Deep Learning for High-Speed Inference. *J. Lightwave Technol., JLT* **40**, 3243–3254 (2022).
3. Kariyappa, S. *et al.* Noise-Resilient DNN: Tolerating Noise in PCM-Based AI Accelerators via Noise-Aware Training. *IEEE Transactions on Electron Devices* **68**, 4356–4362 (2021).
4. Mourgias-Alexandris, G. *et al.* Noise-resilient and high-speed deep learning with coherent silicon photonics. *Nat Commun* **13**, 5572 (2022).
5. Wu, C. *et al.* Harnessing optoelectronic noises in a photonic generative network. *Science Advances* **8**, eabm2956 (2022).
6. Xu, T. *et al.* Physical neural networks using sharpness-aware training. *Nat Commun* **17**, 1766 (2026).
7. Zhang, W., Wang, Y., Lederman, J. C., Shastri, B. J. & Prucnal, P. R. Compact, reconfigurable, and scalable photonic neurons by modulation-and-weighting microring resonators. *eLight* **6**, 6 (2026).
8. Feng, H. *et al.* Integrated lithium niobate microwave photonic processing engine. *Nature* **627**, 80–87 (2024).
9. LeCun, Y., Bottou, L., Bengio, Y. & Haffner, P. Gradient-based learning applied to document recognition. *Proceedings of the IEEE* **86**, 2278–2324 (1998).
10. Liu, S. *et al.* Calibration-free and precise programming of large-scale ring resonator circuits. *Optica, OPTICA* **12**, 1113–1121 (2025).
11. Zhang, W. *et al.* A system-on-chip microwave photonic processor solves dynamic RF interference in real time with picosecond latency. *Light Sci Appl* **13**, 14 (2024).
12. Weigend, A. S. & Gershenfeld, N. A. Results of the time series prediction competition at the Santa Fe Institute. in *IEEE International Conference on Neural Networks* 1786–1793 vol.3 (1993). doi:10.1109/ICNN.1993.298828.
